# Reversible Recognition-Based Boronic Acid Probes for Glucose Detection in Live Cells and Zebrafish

**DOI:** 10.1021/jacs.2c13694

**Published:** 2023-04-06

**Authors:** Kai Wang, Ruixiao Zhang, Xiujie Zhao, Yan Ma, Lijuan Ren, Youxiao Ren, Gaofei Chen, Dingming Ye, Jinfang Wu, Xinyuan Hu, Yuanqiang Guo, Rimo Xi, Meng Meng, Qingqiang Yao, Ping Li, Qixin Chen, Tony D. James

**Affiliations:** † Institute of Materia Medica, Science and Technology Innovation Center, Shandong First Medical University & Shandong Academy of Medical Sciences, Jinan, Shandong 250062, People’s Republic of China; ‡ State Key Laboratory of Medicinal Chemical Biology, College of Pharmacy and KLMDASR of Tianjin, Nankai University, Tongyan Road, Haihe Education Park, Tianjin 300350, People’s Republic of China; § College of Chemistry, Chemical Engineering and Materials Science, Key Laboratory of Molecular and Nano Probes, Ministry of Education, Collaborative Innovation Center of Functionalized Probes for Chemical Imaging in Universities of Shandong, Institutes of Biomedical Sciences, Shandong Normal University, Jinan 250014, People’s Republic of China; ∥ Department of Chemistry, University of Bath, Bath BA2 7AY, U.K.; ⊥ School of Chemistry and Chemical Engineering, Henan Normal University, Xinxiang 453007, People’s Republic of China

## Abstract

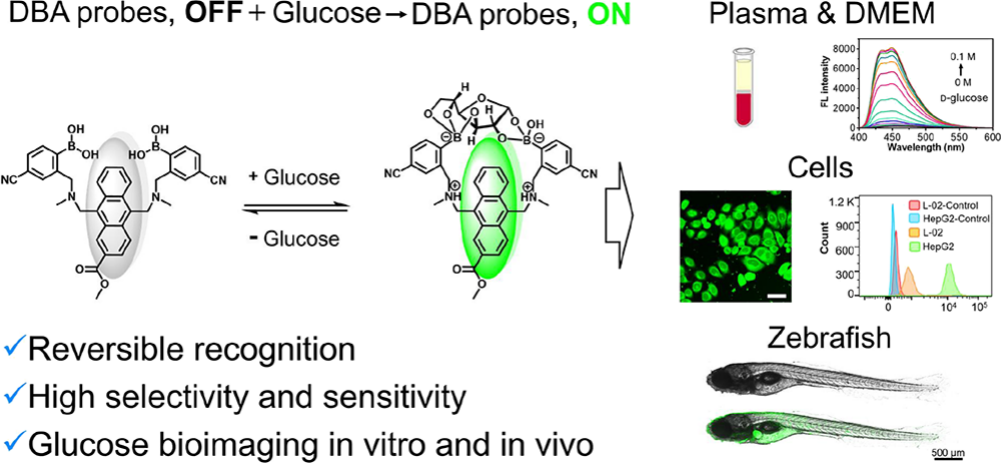

Glucose, a critical source of energy, directly determines the homeostasis of the human body. However, due to the lack of robust imaging probes, the mechanism underlying the changes of glucose homeostasis in the human body remains unclear. Herein, diboronic acid probes with good biocompatibility and high sensitivity were synthesized based on an *ortho*-aminomethylphenylboronic acid probe, phenyl(di)boronic acid (PDBA). Significantly, by introducing the water-solubilizing group −CN directly opposite the boronic acid group and −COOCH_3_ or −COOH groups to the β site of the anthracene in PDBA, we obtained the water-soluble probe **Mc-CDBA** with sensitive response (*F*/*F*_0_ = 47.8, detection limit (LOD) = 1.37 μM) and **Ca-CDBA** with the highest affinity for glucose (*K*_a_ = 4.5 × 10^3^ M^–1^). On this basis, **Mc-CDBA** was used to identify glucose heterogeneity between normal and tumor cells. Finally, **Mc-CDBA** and **Ca-CDBA** were used for imaging glucose in zebrafish. Our research provides a new strategy for designing efficient boronic acid glucose probes and powerful new tools for the evaluation of glucose-related diseases.

## Introduction

Glucose plays a vital role in the regulation of functional stability of the human body by maintaining energy homeostasis. Variations of glucose levels are often related to the occurrence and development of diseases such as cancer, Alzheimer’s disease, diabetes, autoimmune diseases, etc.^1−4^ Presently, the commonly used blood glucose detection methods are based on glucose oxidase. However, the activity of glucose oxidase can be easily affected.^5,6^ Meanwhile, small-molecule fluorescent dyes have been proposed to detect glucose due to the advantages of simple synthesis, stable structure, and in situ measurement ability.^7−10^ In 1925, Warburg’s group found that tumor cells require more glucose than normal cells.^11−13^ Therefore, a series of glucose tracking probes for tumor diagnosis, such as ^18^F-FDG^14^ and 2-NBDG,^15^ were designed. However, these probes can only indirectly reflect cellular glucose levels by changes of glucose uptake but cannot directly monitor intracellular glucose changes. In addition, the incomplete metabolism of tracer probes in living cells leads to metabolic stress and cell death (i.e. ^18^F-FDG). Therefore, it is important to design small molecule-based probes to directly detect glucose levels in situ for the evaluation of glucose metabolism-related diseases.

Phenylboronic acid can reversibly bind with 1, 2 or 1, 3-dihydroxyl compounds to form borate esters and has been widely explored in the detection of saccharides and other diols,^16−18^ yet none of the reported probes are capable of the real-time direct monitoring of glucose concentrations in vivo. In 1995, Shinkai’s group synthesized phenyl(di)boronic acid (PDBA), a fluorescent probe selective for glucose.^19^ The *ortho*-aminomethylphenylboronic acid unit of PDBA can recognize glucose under physiological conditions because the *ortho*-position of the phenylboronic acid incorporates an aminomethyl, and the p*K*_a_ of PDBA was reduced to 4.8. However, the poor water-solubility of PDBA requires the addition of an organic solvent (33% MeOH), which limits its application in biological systems. In addition, the emission wavelength of PDBA is short (λ_ex/em_ = 370/423 nm), and thus the probe would cause cell damage during glucose imaging in biological systems. Moreover, intracellular glucose concentrations often vary within the micromolar range, which requires that the probe should have enhanced sensitivity to monitor changes of glucose levels, therefore, the precise imaging of glucose in cells and in vivo remains a challenge.

**Figure 1:**
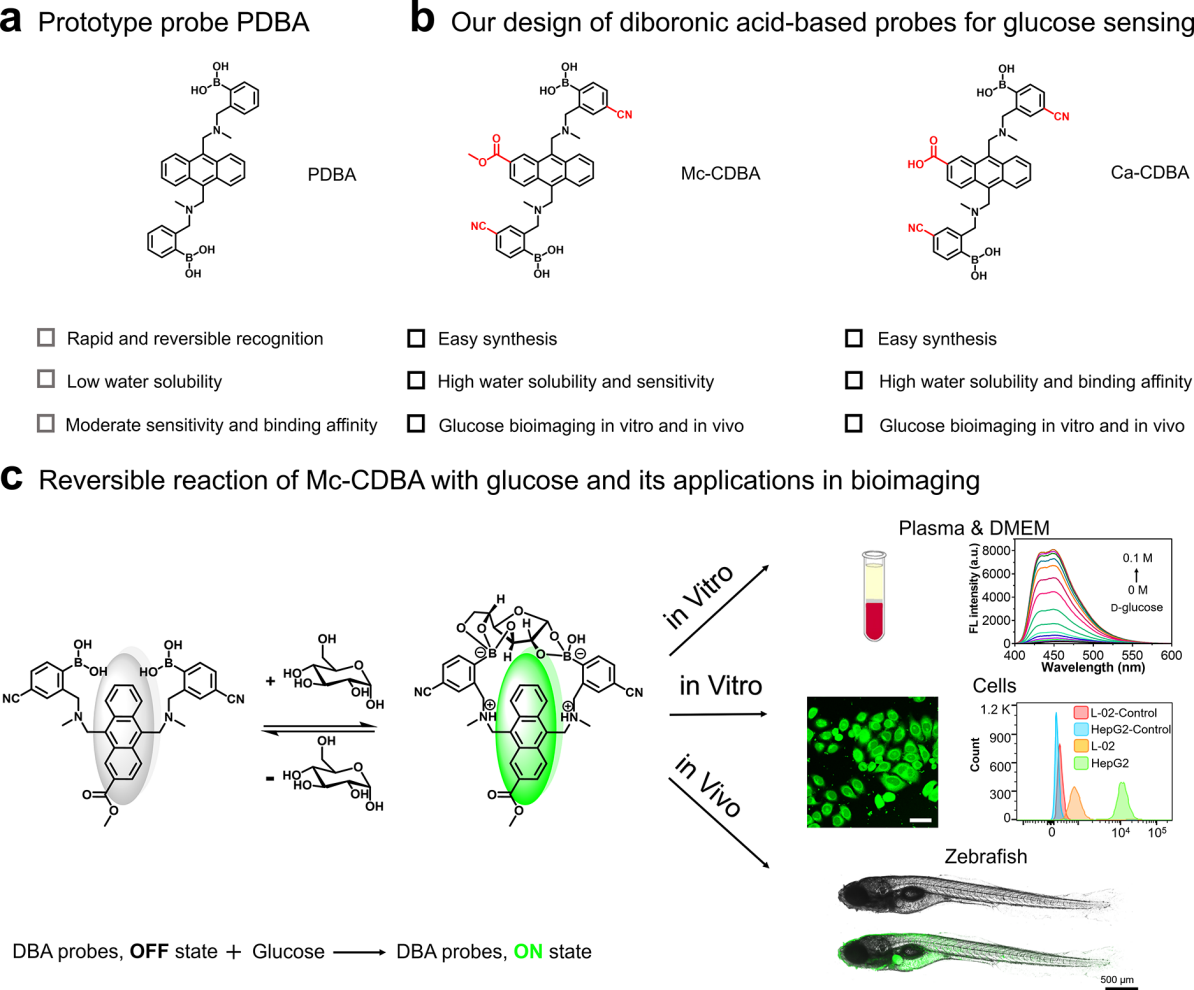
Our strategy for developing diboronic acid-based fluorescent probes for detecting glucose. (a) Structure of the prototype diboronic acid probe PDBA. (b) Molecular design of diboronic acid-based glucose probes. (c) Schematic illustration of the sensing mechanism of **Mc-CDBA** for glucose and its bioimaging applications in this work.

Given that PDBA exhibits good binding affinity for glucose, we used PDBA as the template molecule from which to construct glucose probes with higher water solubility, sensitivity, and red-shifted emission. Therefore, we developed probes **Mc-CDBA** and **Ca-CDBA** in order to evaluate substituent effects on the system. As such a water-solubilizing −CN group^20,21^ was added directly opposite the boronic acid group and −COOCH_3_ and −COOH groups were introduced at the β site of the anthracene (Figure 1a). It was found that the emission peaks were both red-shifted (**Mc-CDBA**, λ_ex/em_ = 393/457 nm; **Ca-CDBA**, λ_ex/em_ = 382/438 nm). In addition, the sensitivity of the probe **Mc-CDBA** (*F*/*F*_0_ = 47.8, 0.1 M Glucose) to glucose was significantly higher than that of PDBA (*F*/*F*_0_ ≈ 14.5, 0.1 M Glucose),^19^ and the detection limit (LOD) was 1.37 μM, which suggests that **Mc-CDBA** was the most sensitive probe among the boronic acid-based glucose probes reported to date. The binding constant of **Ca-CDBA** to glucose (*K*_a_ = 4.5 × 10^3^ M^–1^) was higher than that of **Mc-CDBA** (*K*_a_ = 7.1 × 10^2^ M^–1^) and other reported water-soluble boronic acid probes. We then utilized **Mc-CDBA** and **Ca-CDBA** for glucose detection in Dulbecco’s modified Eagle’s medium (DMEM) and plasma; pointedly the probes exhibited high recovery ratios, good reproducibility, and high precision. The probes exhibit excellent biocompatibility, reversible recognition (Figure 1b), and high sensitivity with minimal cytotoxicity. **Mc-CDBA** was then successfully applied to image glucose in cells and differentiate normal and tumor cells. Notably, in vivo visualization with the aid of the diboronic acid probes revealed the heterogeneity of glucose and the effect of antidiabetic drugs on glucose levels in zebrafish embryos (Figure 1c).

## Results and Discussion

### Synthesis and Photophysical Properties

**Figure 2:**
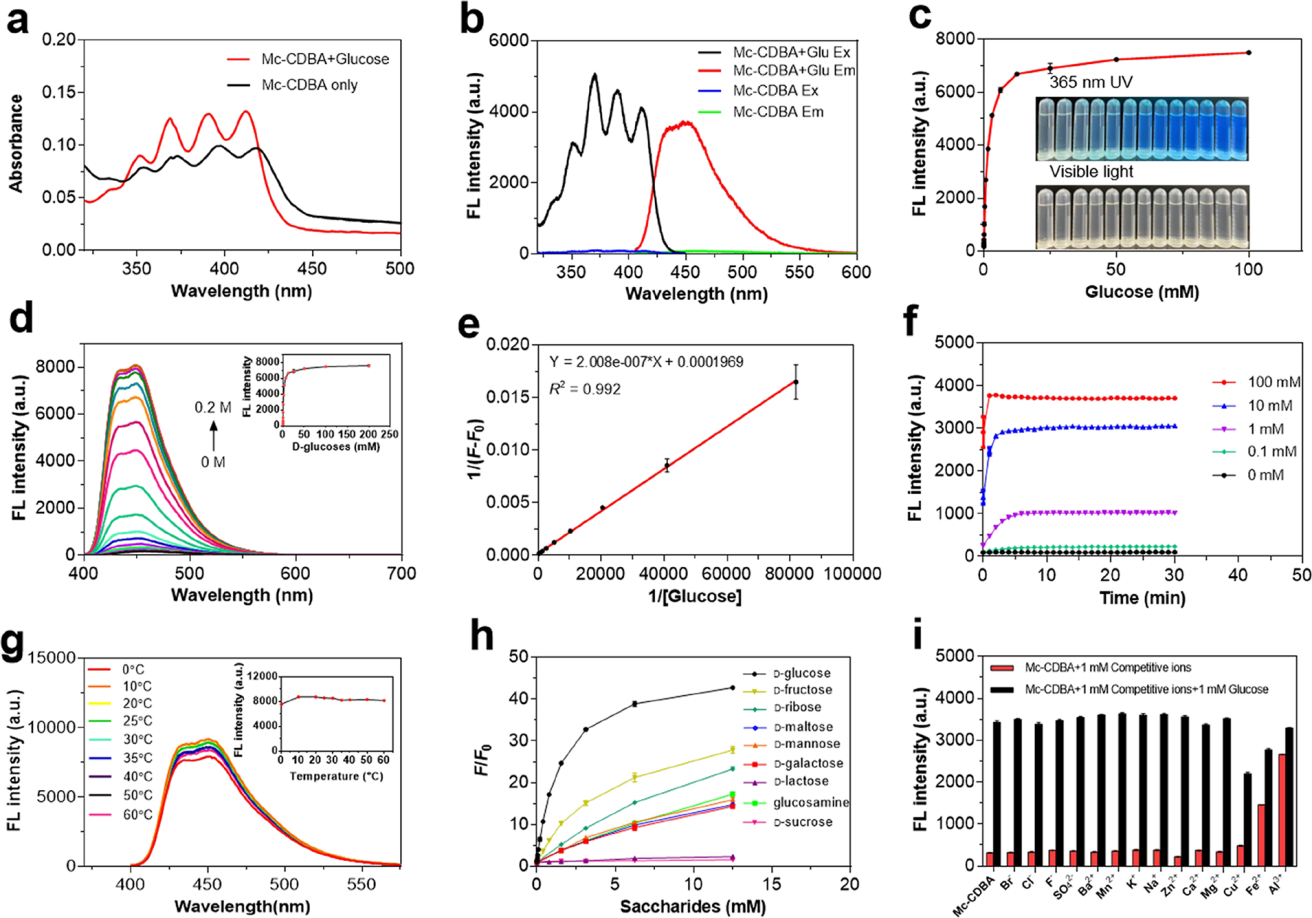
Photophysical properties and selectivity of **Mc-CDBA**. (a) UV–visible absorption spectrum of 10 μM **Mc-CDBA** with or without 0.1 M glucose. (b) Fluorescence excitation and emission spectra of 10 μM **Mc-CDBA** before and after the addition of 0.1 M glucose. (c) Fluorescence intensity increase of 10 μM **Mc-CDBA** upon the addition of increased concentrations (0–0.1 M) of glucose. Inset: Photographs of **Mc-CDBA** with various concentrations of glucose under 365 nm UV and visible light, respectively. (d) Fluorescence spectra of 10 μM **Mc-CDBA** toward increasing concentration of glucose (0–0.2 M). Inset: Plot of fluorescence intensity changes at 457 nm. (e) B–H plot of **Mc-CDBA** in glucose (12.2 μM–12.5 mM) sensing. (f) Time-dependent fluorescence spectra of **Mc-CDBA** (10 μM) in the presence of glucose (0.1–100 mM) for varying time intervals (0–30 min) at 25 °C. (g) Fluorescence spectra changes of 10 μM **Mc-CDBA** versus the solution temperature. Insert: Plot of temperature-dependent fluorescence intensity of 10 μM **Mc-CDBA** with 0.1 M glucose. (h) Fluorescence response (*F*/*F*_0_) of 10 μM **Mc-CDBA** in response to various saccharides including glucose, fructose, ribose, maltose, mannose, galactose, lactose, glucosamine, and sucrose. (i) Comparison of fluorescence intensity of 10 μM **Mc-CDBA** toward various species (1 mM) without or in the presence of glucose (1 mM): blank; Br^–^; Cl^–^; F^–^; SO_4_^2–^; Ba^2+^; Mn^2+^; K^+^; Na^+^; Zn^2+^; Ca^2+^; Mg^2+^; Cu^2+^; Fe^2+^; Al^3+^. All tests were performed in 0.5% MeOH/PBS buffer, pH = 7.4 at 25 °C with λ_ex_ = 393 nm, λ_em_ = 457 nm. Data are presented as the means ± standard deviation (SD) (*n* = 3).

The specific synthetic steps of **Mc-CDBA** and **Ca-CDBA** are shown in Figure S1, and the target compounds were characterized by ^1^H nuclear magnetic resonance (NMR), ^13^C NMR, and high resolution mass spectrometry (HR-MS) (Figures S2–24).^22^ We first explored the optical properties of **Mc-CDBA** and **Ca-CDBA** under simulated physiological conditions of 0.5% MeOH/phosphate buffered saline (PBS) buffer (Figure 2) and 0.5% dimethyl sulfoxide (DMSO)/PBS buffer (Figure S25), respectively. As shown in Figure 2a, **Mc-CDBA** exhibited strong UV absorption in the range of 350 to 425 nm, and the absorption band increased upon the addition of 0.1 M glucose. Next, the excitation and emission spectra of **Mc-CDBA** were examined. In the presence of 0.1 M glucose (Figure 2b), the fluorescence response of **Mc-CDBA** was significantly increased (about 46.8-fold), and the fluorescence quantum yield of **Mc-CDBA** increased from 0.018 to 0.529 (Table S1), which is a better optical performance than **Ca-CDBA** (*F*/*F*_0_ = 9.8). This is probably due to the introduction of the hydroneutral group −COOCH_3_,^23^ which increases the hydrophobicity of anthracene, resulting in enhanced CH-π interactions between the anthracene and glucose, and as a consequence a higher sensitivity.^24^ Meanwhile, there was a significant color variance under UV irradiation at 365 nm (Figure 2c), indicating that **Mc-CDBA** can sensitively recognize glucose. As depicted in Figure 2d, the fluorescence of **Mc-CDBA** at 457 nm gradually enhanced as glucose was added in the range of 0 to 0.2 M. In addition, the Benesi–Hildebrand (B–H) plot (Figure 2e) of the reciprocal of the enhanced fluorescence amplitude 1/(*F* – *F*_0_) of **Mc-CDBA** and the reciprocal of the glucose concentration 1/[*C*]_Glucose_ exhibited a good linear relationship, with a correlation coefficient of 0.992 in the detection range of 12.2 μM–12.5 mM, and the binding ratio of 1:1 is determined. Moreover, we found that **Ca-CDBA** was an *ortho*-aminomethylphenylboronic acid probe with the highest reported binding affinity for glucose (*K*_a_ = 4.5 × 10^3^ M^–1^), which may be due to the −COOH group as a polar and solubilizing group, which is advantageous for glucose binding in aqueous solutions. Conversely the hydroneutral −COOCH_3_ group of **Mc-CBDA** may generate steric hindrance, thus interfering with the glucose binding.^25^

Kinetic experiments (Figures 2f and S25e) indicated that the fluorescence intensity reached equilibrium in about 5 min and stabilized after 25 min, indicating that **Mc-CDBA** and **Ca-CDBA** can rapidly recognize glucose with good photostability. In addition, varying the temperature from 0 to 60 °C had little effect on the fluorescence response of **Mc-CDBA** toward glucose (0.1 M) (Figure 2g). To assess the specificity of **Mc-CDBA** for glucose, we explored the fluorescence responses of **Mc-CDBA** to other interfering substances including common saccharides, inorganic ions, and metal ions. As depicted in Figure 2h, **Mc-CDBA** exhibited significant fluorescence response and binding affinity (*K*_a_) to glucose than other saccharides (Figures S26–27, Table S2). When other interfering ions were added, the fluorescence response of **Mc-CDBA** and **Ca-CDBA** remained nearly unchanged except for Cu^2+^, Fe^2+^, and Al^3+^ (Figures 2i and S25i), which could form a complex with the boronic acids,^26−28^ but these interferences can be ignored since the amount of Cu^2+^, Fe^2+^, and Al^3+^ in normal organisms (μM level) is much lower than that of glucose (mM level).^29−31^ The main metabolic pathway of intracellular glucose is producing energy by aerobic oxidation.^32^ Thus, the fluorescence response of **Mc-CDBA** for various metabolites was evaluated. As shown in Figure S28, **Mc-CDBA** exhibits higher selectivity and sensitivity to glucose than other species. Taken together, these results confirm that **Mc-CDBA** and **Ca-CDBA** can rapidly detect glucose and exhibit good photostability and high specificity.

### Sensitivity and Reaction of the Fluorescent Probes with Glucose

**Figure 3:**
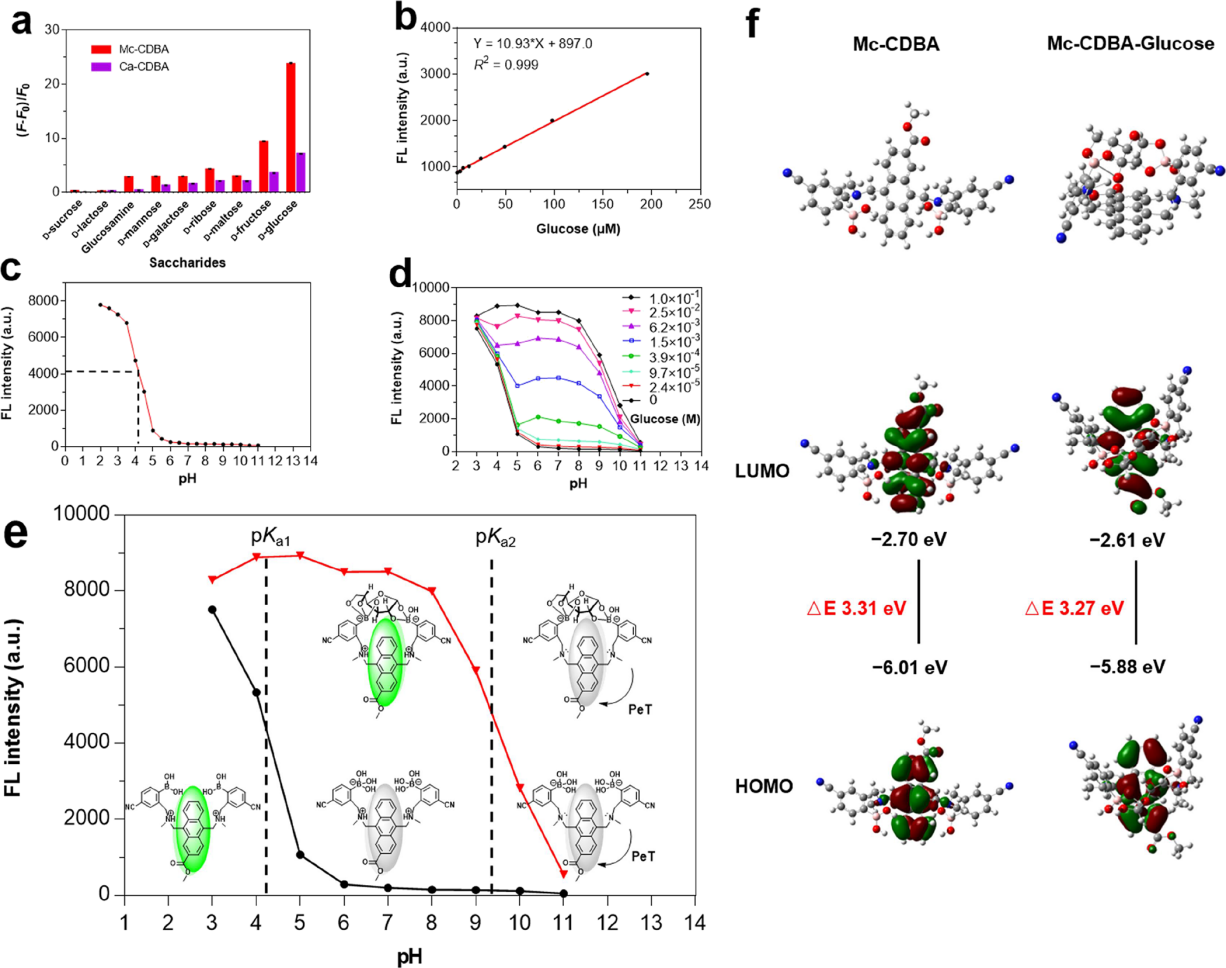
Sensitivity and glucose sensing mechanism of **Mc-CDBA**. (a) Fluorescence changes ((*F* – *F*_0_)/*F*_0_) of **Mc-CDBA** and **Ca-CDBA** in 1.56 mM various saccharides. (b) Linear changes in fluorescence intensity of 10 μM **Mc-CDBA** with various concentrations of glucose (0–195 μM). (c) pH-dependent (pH from 2 to 11) fluorescence emission intensity of 10 μM **Mc-CDBA** in PBS buffer. (d) pH-dependent (pH from 3 to 11) fluorescence emission intensity of 10 μM **Mc-CDBA** with various concentrations of glucose (0–0.1 M). (e) Proposed fluorescence sensing mechanism of **Mc-CDBA** for the turn-on detection of glucose. (f) Structure optimization diagram and theoretical calculation of probe **Mc-CDBA** and its glucose complex. Atom color: gray represents carbon atoms; white represents hydrogen atoms; blue represents nitrogen atoms; pink represents boron atoms; red represents oxygen atoms. **Mc-CDBA** and **Ca-CDBA** were analyzed in 0.5% MeOH/PBS buffer and 0.5% DMSO/PBS buffer, respectively, pH = 7.4 at 25 °C (**Mc-CDBA**, λ_ex/em_ = 393/457 nm; **Ca-CDBA**, λ_ex/em_ = 382/438 nm). Data are presented as the means ± SD (*n* = 3).

From Figure 3a, the probes exhibited significantly larger fluorescence response toward glucose than other saccharides, and **Mc-CDBA** exhibited the strongest glucose response. The fluorescence–concentration (FL–C) plot (Figure 3b) of the fluorescence intensity of **Mc-CDBA** corresponding to glucose concentrations (0–195 μM) is *F* = 10.93 × [Glucose] μM + 897.0, with a correlation coefficient of 0.999, and the detection limit was calculated to be 1.37 μM, indicating that **Mc-CDBA** is the most sensitive probe for glucose. The p*K*_a_ value of **Mc-CDBA** and its glucose complexes calculated from fluorescence measurements in Figure 3c,d was about 4.2 and 9.6, respectively, which were lower than that of the prototype probe PDBA (p*K*_a_ = 4.8 and 11.1, respectively).^19^ This is attributed to the electron-withdrawing −CN group, which lowers the p*K*_a_ of the *para*-boronic acid. Boronic acids with lower p*K*_a_ values tend to have higher affinities for diols at physiological pH. As can be seen from Figures 3d and S29, **Mc-CDBA** and **Ca-CDBA** exhibited stable and sensitive fluorescence responses to glucose over a pH range 5–8 and 6–8, further demonstrating the applicability of the probes in biological systems.

The sensing of glucose by **Mc-CDBA** at different pH probably involves multiple binding types (Figure 3e). The phenylboronic acid group in **Mc-CDBA** is *sp*^2^ hybridized and flat under strong acid conditions, so it cannot bind to glucose. As the pH gradually increases to the p*K*_a_ of **Mc-CDBA**, the probe **Mc-CDBA** begins to react with glucose and exhibits a sensitive and stable fluorescence response over a pH range from 5 to 8. When the pH was increased to strong alkalinity, the protonated amine group is deprotonated, and the resulting nitrogen lone pair of electrons initiate the PeT effect, leading to fluorescence quenching. To better understand the fluorescence turn-on response of **Mc-CDBA** toward glucose, a computational evaluation was performed for **Mc-CDBA**, **Ca-CDBA,** and their glucose-borate compounds using Gaussian 09 software (density functional theory method: B3LYP/6-31G*). As depicted in Figures 3f and S30, the highest occupied molecular orbital (HOMO) and lowest unoccupied molecular orbital (LUMO) levels of these probes were mainly distributed on the conjugate system of anthracene, and the HOMO–LUMO gap of **Mc-CDBA** and **Ca-CDBA** with their glucose-borate compounds is 3.31 eV/3.27 eV and 3.31 eV/3.26 eV, respectively. The smaller the probe band gap, the easier it is to be excited. Thus, these results indicate that the probes were more easily excited and produced a stronger fluorescence response after combining with glucose. All these results confirm that **Mc-CDBA** and **Ca-CDBA** can sense changes in glucose levels.

### DMEM Medium and Plasma Glucose Detection In Vitro

Next, we used probes **Mc-CDBA** and **Ca-CDBA** to detect glucose in complex biological matrices. As shown in Figure S31, the fluorescence responses of both **Mc-CDBA** and **Ca-CDBA** in DMEM medium exhibited a marked change with the addition of glucose. The B-H plots of **Mc-CDBA** and **Ca-CDBA** showed a good linear relationship in the range of glucose concentration from 48.8 μM to 12.5 mM (*R*^2^ = 0.996) and 24.4 μM to 12.5 mM (*R*^2^ = 0.997), respectively, which indicates that the probes could sensitively quantify glucose in complex media. To further verify the viability of this method, recovery experiments for plasma glucose detection were carried out using the B-H plot. The recovery rates for glucose detection by the probes **Mc-CDBA** and **Ca-CDBA** were higher than 91.3 and 96.9%, respectively, and the ability to detect glucose in sheep plasma was similar to that for commercial blood glucose detection kits (Table S3). In addition, the results of intrabatch, interbatch, intraday, and interday analysis indicated that the probes exhibited good reproducibility with variation coefficients lower than 5.15 and 3.78%, respectively (Tables S4–6). These results indicated that complex components in the biological matrix had little effect on glucose recognition, and the probes **Mc-CDBA** and **Ca-CDBA** exhibited good glucose selectivity.

### Glucose Imaging in Living Cells

**Figure 4:**
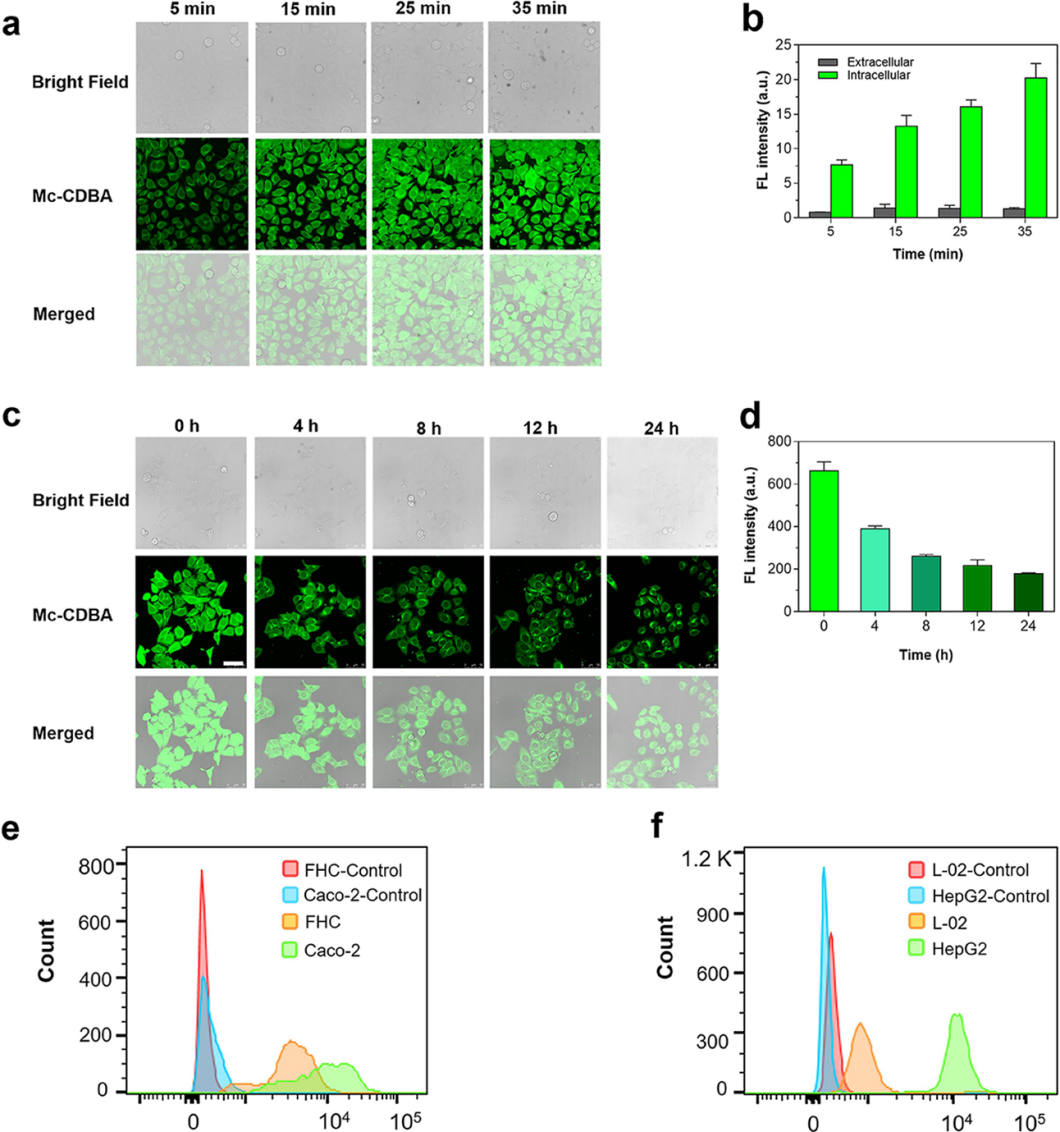
Confocal microscopy imaging of **Mc-CDBA**. (a, b) Confocal microscopy images (a) and the fluorescence intensity comparison between extracellular and intracellular regions (b) of HeLa cells incubated with 50 μM **Mc-CDBA** for 5, 15, 25, and 35 min, respectively. (c, d) Confocal microscopy images (c) and fluorescence intensity (d) of HeLa cells preincubated with glucose-free DMEM for 0, 4, 8, 12, and 24 h, then incubated with 50 μM **Mc-CDBA** for 30 min. (e) Flow images of probe **Mc-CDBA** distinguishing Caco-2 cells from FHC cells. (Red: FHC-Control cells; Blue: Caco-2-Control cells; Yellow: FHC cells with **Mc-CDBA**; Green: Caco-2 cells with **Mc-CDBA**). (f) Flow images of probe **Mc-CDBA** distinguishing HepG2 cells from L-02 cells. (Red: L-02-Control cells; Blue: HepG2-Control cells; Yellow: L-02 cells with **Mc-CDBA**; Green: HepG2 cells with **Mc-CDBA**). Cell images were captured on a Leica TCS SP8 with λ_ex/em_ = 405/410–600 (scale bar = 50 μm).

Since **Mc-CDBA** and **Ca-CDBA** can sensitively and selectively recognize glucose in a complex matrix, the ability of the probes for the imaging of glucose in cells was then evaluated. First, the cytotoxicity of **Mc-CDBA** and **Ca-CDBA** toward HeLa, HepG2, and L-02 cells was evaluated using an MTT assay (Figure S32), and no significant cytotoxicity was observed toward those cells in the concentration range of 0–100 μM. After incubation with 50 μM **Mc-CDBA**, the fluorescence intensity of HeLa cells increased rapidly within 5 min and then reached a plateau at about 30 min (Figure S33), while the uptake of probe **Ca-CDBA** was relatively slow and approached saturation at about 40 min. The fluorescence in HeLa cells was mainly concentrated in the cytoplasm, indicating that glucose was mainly distributed in the cytoplasm, which is consistent with previous reports.^33^ As shown in Figure 4a,b, at 5 min, a clear fluorescence difference was observed in the inner and outer regions of HeLa cells. With a prolonged incubation time for **Mc-CDBA**, the fluorescence difference gradually increased (25 min), while the fluorescence difference for **Ca-CDBA** was negligible even with extended incubation (Figure S34a,b). This result indicates that **Mc-CDBA** was better than **Ca-CDBA** for visualizing glucose at a cellular level.

To further explore the ability of the probes for cell imaging, we attempted to visualize glucose consumption in HeLa cells. As shown in Figures 4c,d and S34c,d, a remarkable decrease of fluorescence in HeLa cells was observed within 4 h, and the fluorescence intensity of the cells gradually decreased with extended cell starvation time from 4 to 24 h. These results indicated that both **Mc-CDBA** and **Ca-CDBA** were able to sensitively respond to changes of intracellular glucose levels. Compared with normal cells, tumor cells require more glucose for energy and their growth, invasion, and metastasis. In order to further explore the practical ability of the diboronic acid probes for cell imaging, we used **Mc-CDBA** to visualize the intracellular glucose levels in two groups of normal and tumor cells, fetal human cells (FHC) and Caco-2, L-02, and HepG2. These cells were incubated with **Mc-CDBA** for 30 min followed by measurement using a flow cytometer. As shown in Figure 4e,f, the fluorescence intensity of the tumor cells (Caco-2 and HepG2 cells) was higher than that of the normal cells (FHC and L-02 cells), indicating that tumor cells contain higher glucose levels, which was consistent with previous reports^34,35^ and the Warburg effect. The above results revealed that **Mc-CDBA** could efficiently differentiate tumor and normal cells and has potential applications for the diagnosis of cancer-related diseases.

### Fluorescence Imaging of Zebrafish

**Figure 5:**
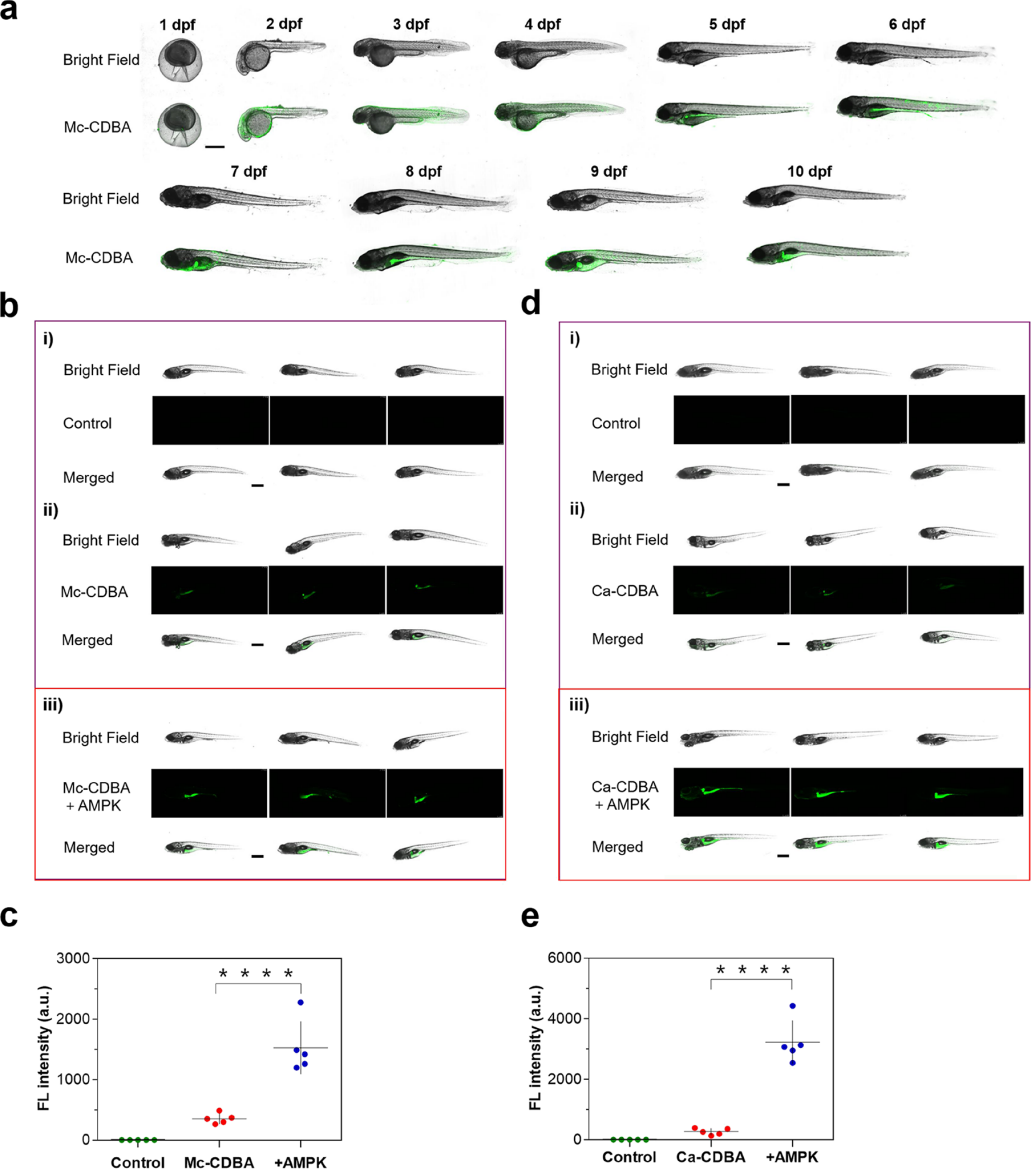
Fluorescence imaging of **Mc-CDBA** and **Ca-CDBA** in zebrafish. (a) Confocal microscopy images of 1–10 dpf zebrafish embryos incubated with 50 μM **Mc-CDBA** for 3 h. (b, c) Fluorescence confocal images (b) and fluorescence intensity (c) of 7 dpf zebrafish embryos preincubated with blank medium (i), blank medium (ii), and 20 μM ampkinone medium (iii) for 4 h, then group ii and iii further incubated with 50 μM **Mc-CDBA** for 1 h. (d, e) Fluorescence confocal images (d) and fluorescence intensity (e) of 7 dpf zebrafish embryos preincubated with blank medium (i), blank medium (ii), and 20 μM ampkinone medium (iii) for 4 h, then group ii and iii further incubated with 50 μM **Ca-CDBA** for 1 h. Zebrafish images were captured on a Leica TCS SP8 with λ_ex/em_ = 405/410–600 nm (*n* = 5, with levels of significance set at **** *P* < 0.0001, extremely significant). Scale bar represents 500 μm.

Zebrafish, a commonly used biomedical animal model, is convenient for breeding, and the embryo is transparent for easy imaging.^36^ Therefore, the realization of sensitive detection of glucose levels in zebrafish will be of great significance for the diagnosis and effective evaluation of diseases (Table S7). To explore whether **Mc-CDBA** and **Ca-CDBA** could be used for in vivo imaging, we explored the uptake of the probes in zebrafish embryos. As shown in Figure S35, the probe **Mc-CDBA** began to illuminate zebrafish embryos at about 1 h, and the fluorescence intensity increased significantly with an extension of incubation time, which clearly confirms the excellent ability for zebrafish imaging. Meanwhile, the imaging ability of **Ca-CDBA** was less satisfactory, and it could not light up zebrafish until after 3 h. Subsequently, **Mc-CDBA** and **Ca-CDBA** were used to image glucose levels in zebrafish embryos at different stages. As shown in Figures 5a and S36, the fluorescence intensity of zebrafish embryos at 1–4 day post-fertilization (dpf) was weak, indicating that the glucose content in zebrafish is low at that time, and the fluorescence of zebrafish embryos at 5–10 dpf was mainly concentrated in the pancreas, liver, intestine, and other parts, indicating that glucose is mainly distributed in these parts of zebrafish embryos at that time, which was consistent with the research of Jurczyk et al.^37^ In conclusion, **Mc-CDBA** and **Ca-CDBA** are able to sensitively respond to the changes of glucose level in zebrafish embryos.

Based on the imaging capability of **Mc-CDBA** and **Ca-CDBA** toward glucose in zebrafish, we examined whether the probes could be used to screen drug activity. Ampkinone, an activator of AMP protein kinase, can enhance the glucose uptake in zebrafish. As shown in Figure 5b–e, weak fluorescence was observed for the control group where the zebrafish was incubated with **Mc-CDBA** or **Ca-CDBA** only, while bright fluorescence was observed for the zebrafish treated with ampkinone (****P < 0.0001), revealing that the glucose level of zebrafish in the ampkinone treatment group were higher than that in the non-ampkinone treatment group, which is consistent with the previous report.^38^ 4,6-EDG, a glucose transporter inhibitor and a glucose analogue, can inhibit the uptake of glucose in an organism. Figure S37 shows that the fluorescence intensity of **Mc-CDBA** and **Ca-CDBA** with 4,6-EDG treated groups was slightly lower than that of the control group, but the difference between the two groups was not statistically significant (*P* > 0.05). This may be because the culture environment of the zebrafish was high in glucose (10 mM), which could reduce the effect of 4,6-EDG on the zebrafish, resulting in interference and inconclusive imaging results. As such, glucose imaging using probes **Mc-CDBA** and **Ca-CDBA** in zebrafish embryos exhibits significant potential for the screening of diabetes-related drugs.

## Conclusions

With this research we developed two *ortho*-aminomethylphenylboronic acid-based probes, **Mc-CDBA** and **Ca-CDBA**, for the detection of glucose in vitro and in vivo through in situ fluorescence imaging. Compared with other glucose tracers and enzyme probes, the diboronic acid probes can directly report on intracellular glucose due to the reversible, dynamic, and in situ recognition. Owing to their unique ability to recognize intracellular glucose, **Mc-CDBA** and **Ca-CDBA** can be used to distinguish and determine the cell status, as well as assess and screen potential drugs for glucose-related diseases.

Compared with the reported boronic acid probes, our probes provide a novel design strategy for incorporating different functional groups, the water-solubilizing group −CN and the −COOH and −COOCH_3_ groups, to improve the physiochemical properties of the PDBA probe, so that it exhibits good biocompatibility and excellent sensitivity, facilitating glucose bioimaging. This design method could be generalized for the development of specific boronic acid probes for the sensing of other important physiological substances, such as catechol, sugars, fluoride ion, etc.

## Supporting Information Available

Additional experimental data including experimental details; synthesis of **Mc-CDBA** and **Ca-CDBA**; NMR and HR-MS spectra; photophysical properties and selectivity; fluorescence imaging of live cells and zebrafish; sensitivity and glucose sensing mechanism; structure optimization diagram and theoretical calculation; glucose detection; cell viability and cell uptake assay; confocal microscopy imaging of zebrafish; uptake tests; efficacy evaluation test; characteristics of the diboronic acid-based probes for glucose; binding affinity (K_a_) of the probes; results of glucose detection; interday variations of plasma glucose detection; key information of the reported boronic acid-based glucose probes (PDF)
