# Supplementary material for: Reversible Recognition-Based Boronic Acid Probes for Glucose Detection in Live Cells and Zebrafish
Source: J Am Chem Soc. 2023 Apr 6;145(15):8408–16. doi: 10.1021/jacs.2c13694 (PMC10119935; doi:10.1021/jacs.2c13694)
Supplement: Supplementary file 1 — ja2c13694_si_001.pdf [file ja2c13694_si_001.pdf]

Reversible recognition-based boronic acid probes for glucose detection in live cells and zebrafish

Kai Wang,[ad] Ruixiao Zhang,[b] Xiujie Zhao,[b]* Yan Ma,[b] Lijuan Ren,[a] Youxiao Ren,[a] Gaofei Chen,[a] Dingming Ye,[a] Jinfang Wu,[a] Xinyuan Hu,[a] Yuanqiang Guo,[b] Rimo Xi,[b] Meng Meng,[b] Qingqiang Yao,[a]* Ping Li,[c]* Qixin Chen,[a]* Tony D. James,[de]*

a. Institute of Materia Medica, Science and Technology Innovation Center, Shandong First Medical University & Shandong Academy of Medical Sciences, Jinan 250062, Shandong, People's Republic of China
b. State Key Laboratory of Medicinal Chemical Biology, College of Pharmacy and KLMDASR of Tianjin, Nankai University, Tongyan Road, Haihe Education Park, Tianjin 300350, People's Republic of China
c. College of Chemistry, Chemical Engineering and Materials Science, Key Laboratory of Molecular and Nano Probes, Ministry of Education, Collaborative Innovation Center of Functionalized Probes for Chemical Imaging in Universities of Shandong, Institutes of Biomedical Sciences, Shandong Normal University, Jinan 250014, People's Republic of China
d. Department of Chemistry, University of Bath, Bath BA2 7AY, U.K.
e. School of Chemistry and Chemical Engineering, Henan Normal University, Xinxiang 453007, People's Republic of China

***Corresponding author:**

Tony D. James:

E-mail: t.d.james@bath.ac.uk

Qixin Chen:

E-mail: chenqixin@sdfmu.edu.cn

Ping Li:

E-mail: lip@sdnu.edu.cn

Qingqiang Yao:

E-mail: qqyao@sdfmu.edu.cn

Xiujie Zhao:

E-mail: 9820200042@nankai.edu.cn

Supplementary Methods

Supplementary Figure 1. Synthetic routes for **Mc-CDBA** and **Ca-CDBA**.

Supplementary Figure 2. ^{1}H NMR spectrum of compound **2**.

Supplementary Figure 3. ^{1}H NMR spectrum of compound **3**.

Supplementary Figure 4. ^{1}H NMR spectrum of compound **5**.

Supplementary Figure 5. ^{13}C NMR spectrum of compound **5**.

Supplementary Figure 6. High resolution mass spectrum (HR-MS) of compound **5**.

Supplementary Figure 7. ^{1}H NMR spectrum of compound **6**.

Supplementary Figure 8. ^{13}C NMR spectrum of compound **6**.

Supplementary Figure 9. High resolution mass spectrum (HR-MS) of compound **6**.

Supplementary Figure 10. ^{1}H NMR spectrum of compound **7**.

Supplementary Figure 11. ^{13}C NMR spectrum of compound **7**.

Supplementary Figure 12. High resolution mass spectrum (HR-MS) of compound **7**.

Supplementary Figure 13. ^{1}H NMR spectrum of compound **8**.

Supplementary Figure 14. ^{13}C NMR spectrum of compound **8**.

Supplementary Figure 15. High resolution mass spectrum (HR-MS) of compound **8**.

Supplementary Figure 16. ^{1}H NMR spectrum of compound **9**.

Supplementary Figure 17. ^{13}C NMR spectrum of compound **9**.

Supplementary Figure 18. High resolution mass spectrum (HR-MS) of compound **9**.

Supplementary Figure 19. ^{1}H NMR spectrum of compound **Mc-CDBA**.

Supplementary Figure 20. ^{13}C NMR spectrum of compound **Mc-CDBA**.

Supplementary Figure 21. High resolution mass spectrum (HR-MS) of compound **Mc-CDBA**.

Supplementary Figure 22. ^{1}H NMR spectrum of compound **Ca-CDBA**.

Supplementary Figure 23. ^{13}C NMR spectrum of compound **Ca-CDBA**.

Supplementary Figure 24. High resolution mass spectrum (HR-MS) of compound **Ca-CDBA**.

Supplementary Figure 25. Photophysical properties and selectivity of **Ca-CDBA**.

Supplementary Figure 26. Fluorescence response of **Mc-CDBA** with various saccharides (0–0.2 M).

Supplementary Figure 27. Fluorescence response of **Ca-CDBA** with various saccharides (0–0.2 M).

Supplementary Figure 28. Comparison of fluorescence intensity of 10 μM **Mc-CDBA** towards various glucose metabolic species (1 mM) with or without glucose (1 mM).

Supplementary Figure 29. Sensitivity and glucose sensing mechanism of **Ca-CDBA**.
Supplementary Figure 30. Structure optimization diagram and theoretical calculation of probe **Ca-CDBA** and its glucose borate compound.
Supplementary Figure 31. Glucose detection of **Mc-CDBA** and **Ca-CDBA** in DMEM Medium.
Supplementary Figure 32. Cell viability assay.
Supplementary Figure 33. Cell uptake assay of **Mc-CDBA** and **Ca-CDBA** in HeLa cells.
Supplementary Figure 34. Confocal microscopic imaging of **Ca-CDBA**.
Supplementary Figure 35. Zebrafish uptake tests of **Mc-CDBA** and **Ca-CDBA**.
Supplementary Figure 36. Fluorescence imagining of **Ca-CDBA** for 1−10 dpf zebrafish.
Supplementary Figure 37. Efficacy evaluation tests of 4,6-EDG in zebrafish embryos.
Supplementary Table 1. Characteristics of the diboronic acid-based probes for glucose.
Supplementary Table 2. Binding affinity (K_a) of the probes for different saccharides.
Supplementary Table 3. The results of glucose detection in sheep plasma by commercial glucose analysis kit and the probe **Mc-CDBA**.
Supplementary Table 4. The inter-day variations of plasma glucose detection by **Mc-CDBA**.
Supplementary Table 5. The results of glucose detection in sheep plasma by commercial glucose analysis kit and the probe **Ca-CDBA**.
Supplementary Table 6. The inter-day variations of plasma glucose detection by **Ca-CDBA**.
Supplementary Table 7. Key information of the reported boronic acid-based glucose probes.
References

Supplementary Methods

Chemical synthesis

Materials and instruments： All commercially available reagents were used as specified without further purification. 9,10-dimethylanthracene, 2,2-dimethyl-1,3-propanediol, KI, *N*-bromosuccinimide and aluminum chloride were purchased from Tianjin Heowns Biochemical Technology Co., Ltd. O-tolylboronic acid and (4-cyano-2-methylphenyl)boronic acid were purchased from Bide Pharmatech Co., Ltd. (Shanghai, China). Benzoylperoxide was purchased from Beijing Innochem Science & Technology Co., Ltd. Methylamine methanol, carbon disulfide, 1-phenyl-2-thioure and sodium carboxymethylcellulose were purchased from Shanghai Aladdin Bio-Chem Technology Co., Ltd. Acetyl chloride was purchased from Infinity Scientific (Beijing) Co. Ltd. DMEM was purchased from GIBCO medium (Invitrogen, Camarillo, CA). DMEM (without glucose) was from GIBCO® DMEM medium (#11966025, Thermo Fisher Scientific). D-glucose, D-fructose, D-ribose, D-galactose, D-mannose, glucosamine, D-lactose and D-sucrose were purchased from Shanghai Yuanye Bio-Technology Co., Ltd. Blood sugar assay kit was purchased from Solarbio Science & Technology Co., Ltd. (Beijing, China). 4, 6-EDG was purchased from Beijing Chemsynlab Co., Ltd. Ampkinone was purchased from MedChemExpress LLC. Tricaine methanesulfonate was purchased from Shanghai Dibai Bio-Chem Technology Co., Ltd. Fetal bovine serum was purchased from SeraPro FBS (SYSTECH GmbH, Germany). Malic acid, Oxaloacetic acid, *a*-Ketoglutaric acid, L-(+)-Lactic acid, D-Glucose-6-phosphate, D-Fructose-1,6-bisphosphate, D-Fructose-1,6-bisphosphate, Uridine diphosphate glucose, D-Ribulose 5-phosphate were purchased from Sigma Aldrich (Shanghai) Co., Ltd. UV-visible absorption spectra were accomplished on Hitachi UH5300 double-beam UV-Vis spectrophotometer (Japan). Fluorescence emission (FL) spectra were carried out using a Hitachi F-7000 FL Spectrophotometer (Japan). ^{1}H NMR and ^{13}C NMR spectra were recorded on a Bruker Advance 400 MHz and 800 MHz Spectrometer (Bruker, Germany). High-resolution mass spectra (HR-MS) were obtained on Bruker En Apex ultra7.0T FT-MS.

Syntheses of Mc-CDBA and Ca-CDBA

The diboronic acid probes, **Mc-CDBA** and **Ca-CDBA**, were prepared according to the synthetic routes in

Figure S1.[1] The detailed synthetic procedures of **Mc-CDBA** and **Ca-CDBA** are described below.

4-(5,5-dimethyl-1,3,2-dioxaborinan-2-yl)-3-methylbenzonitrile (compound 2): (4-cyano-2-methylphenyl)boronic acid (5.00 g, 31.06 mmol) was mixed with 2,2-dimethyl-1,3-propanediol (3.88 g, 37.25 mmol) and 200 mL toluene in a 500 mL round bottom flask, and refluxed in an oil bath with the Dean-Stark trap for 20 h. After the reaction was completed, the solvent was evaporated under vacuum, and the mixture was purified by silica gel column chromatograph using dichloromethane as the eluent to obtain compound **2** in 85.8% yield (6.11 g). ^{1}H NMR (400 MHz, Chloroform-*d*) δ 7.79 (d, *J* = 7.5 Hz, 1H), 7.41 (d, *J* = 7.7 Hz, 2H), 3.79 (s, 4H), 2.52 (s, 3H), 1.04 (s, 6H).

3-(bromomethyl)-4-(5,5-dimethyl-1,3,2-dioxaborinan-2-yl)benzonitrile (compound 3): Compound **2** (6.0 g, 26.19 mmol), *N*-bromosuccinimide (5.52 g, 30.99 mmol), AIBN (0.13 g, 0.79 mmol) were mixed in 200 mL carbon tetrachloride. After the solution was refluxed and stirred for 16 h, the solvent was allowed to cool to RT and then filtered. The crude product was separated by gel chromatography with the eluent as petroleum ether/ethyl acetate (10/1, v/v) and the white product **3** was obtained in 97.9% yield (7.87 g). ^{1}H NMR (400 MHz, Chloroform-*d*) δ 7.89 (d, *J* = 7.7 Hz, 1H), 7.62 (s, 1H), 7.53 (dd, *J* = 7.7, 1.6 Hz, 1H), 4.90 (s, 2H), 3.82 (s, 4H), 1.06 (s, 6H).

1-(9,10-dimethylanthracen-2-yl)ethan-1-one (compound 5): Aluminum chloride (3.74 g, 28.05 mmol) was mixed with 9,10-dimethylanthracene **4** (4.00 g, 19.39 mmol), distilled acetylchrolide (2.15 mL, 30.25 mmol) and 215 mL carbondisulfide in a round bottom flask, and stirred at room temperature for 12 h. Then the mixture was heated at 45 °C for 2 h. Subsequently, 65 mL of cracked ice with 3.4 mL hydrogen chloride was added and the reaction mixture was allowed to cool to RT. The mixture was extracted with 150 mL of chloroform, evaporated under vacuum, and then purified by silica gel chromatography using dichloromethane as the eluent to obtain compound **5** in 63.3% yield (3.05 g). ^{1}H NMR (400 MHz, Chloroform-*d*) δ 9.00 (d, *J* = 1.5 Hz, 1H), 8.37–8.31 (m, 3H), 8.00 (dd, *J* = 9.2, 1.8 Hz, 1H), 7.61–7.54 (m, 2H), 3.17 (s, 3H), 3.08 (s, 3H), 2.79 (s, 3H). ^{13}C NMR (101 MHz, $CDCl_3$) δ 198.39, 133.05, 131.54,

131.51, 130.90, 130.39, 128.71, 128.69, 128.61, 126.16, 126.01, 125.68, 125.49, 125.41, 122.02, 26.75, 14.40, 14.27. HRMS (ESI$^+$): calcd for $C_{18}H_{16}ONa$ $[M+Na]^+$ 271.1093, found 271.1100.

9,10-dimethylanthracene-2-carboxylic acid (compound 6): In a round-bottom flask, 1-(9,10-dimethylanthracen-2-yl)ethan-1-one **5** (0.86 g, 3.46 mmol) was mixed with 30 mL of dioxane, sodium hypochlorite (10 ~ 13%, 5.0 mL) and sodium hydroxide (6.7% (w/v), 3.5 mL). Then, the reaction mixture was stirred at 85 °C for 8 h. After that, the reaction mixture was diluted with 5 mL of water, and acidified with hydrochloric acid. The reaction mixture was filtered, washed with little water, evaporated under vacuum to obtain compound **6** as a yellow powder in 83.0% yield (719.3 mg). ^{1}H NMR (400 MHz, DMSO-d_6) δ 13.15 (s, 1H), 8.98 (s, 1H), 8.35 (s, 3H), 7.92 (d, J = 8.6 Hz, 1H), 7.59 (s, 2H), 3.02 (d, J = 17.8 Hz, 6H). ^{13}C NMR (101 MHz, DMSO) δ 167.64, 130.69, 130.24, 129.72, 128.47, 128.40, 128.15, 126.68, 126.14, 125.90, 125.57, 125.50, 125.34, 123.36, 13.89. HRMS (ESI$^-$): calcd for $C_{17}H_{13}O_2$ $[M-H]^-$ 249.0921, found 249.0923.

methyl 9,10-dimethylanthracene-2-carboxylate (compound 7): 9,10-dimethylanthracene-2-carboxylic acid **6** (150 mg, 0.60 mmol) was mixed with 80 mL of absolute methanol and 0.2 mL of sulfuric acid in a round bottom flask, and refluxed for 20 h. The solvent was evaporated under vacuum. After that, the mixture was diluted with 50 mL of chloroform, washed with 5% sodium bicarbonate aqueous solution and saturated aqueous sodium chloride. The solvent was evaporated under vacuum and purified by silica gel chromatography using dichloromethane as the eluent to obtain compound **7** in 78.9% yield (125 mg).^{1}H NMR (400 MHz, Chloroform-d) δ 9.15 (d, J = 1.3 Hz, 1H), 8.34 (dd, J = 9.0, 7.0 Hz, 3H), 8.01 (dd, J = 9.2, 1.5 Hz, 1H), 7.57 (td, J = 6.2, 5.6, 2.6 Hz, 2H), 4.03 (s, 3H), 3.16 (s, 3H), 3.08 (s, 3H). ^{13}C NMR (101 MHz, $CDCl_3$) δ 167.76, 131.41, 131.04, 130.39, 129.58, 128.86, 128.66, 126.07, 125.84, 125.76, 125.49, 125.36, 123.33, 52.42, 14.49, 14.35. HRMS (ESI$^+$): calcd for $C_{18}H_{16}O_2Na$ $[M+Na]^+$ 287.1043, found 287.1047.

methyl 9,10-bis(bromomethyl)anthracene-2-carboxylate (compound 8): In a three-necked round-bottom flask, methyl 9,10-dimethylanthracene-2-carboxylate **7** (468 mg, 1.77 mmol) was mixed with N-bromosuccinimide (702 mg, 3.94 mmol), benzoylperoxide (6.5 mg, 26.78 μmol), 6 mL of chloroform and 16 mL of carbontetrachloride. The mixture was refluxed for 1.8 h. Then the mixture was evaporated under vacuum, and 50 mL of methanol was added and stirred for 10 min. After that, the mixture was filtered and washed with washed with 5 mL of methanol. The solid was dried under vacuum, to give compound **8** in 98.0% yield (732.3 mg). ^{1}H NMR (400 MHz, Chloroform-*d*) δ 9.16 (s, 1H), 8.40 (t, *J* = 9.0 Hz, 3H), 8.20 (d, *J* = 9.2 Hz, 1H), 7.78–7.69 (m, 2H), 5.53 (d, *J* = 26.3 Hz, 4H), 4.05 (s, 3H). ^{1}H NMR (800 MHz, DMF-d_7) δ 9.27 (s, 1H), 8.73 (d, *J* = 9.1 Hz, 1H), 8.66 (dd, *J* = 23.4, 8.4 Hz, 2H), 8.20 (d, *J* = 9.1 Hz, 1H), 7.90 – 7.81 (m, 2H), 5.94 (d, *J* = 40.3 Hz, 4H), 4.05 (s, 3H). ^{13}C NMR (201 MHz, Pyr) δ 167.51, 134.71, 132.70, 132.00, 131.69, 131.12, 129.48, 129.08, 129.02, 128.81, 128.51, 126.90, 126.24, 126.09, 125.95, 53.30, 28.54, 28.17. HRMS (ESI$^+$): calcd for $C_{18}H_{14}Br_2ONa$ [M+Na]$^+$ 442.9253, found 442.9253.

methyl 9,10-bis((methylamino)methyl)anthracene-2-carboxylate (compound 9) : methyl 9,10-bis(bromomethyl)-anthracene-2-carboxylate **8** (0.65 g, 1.54 mmol) was dissolved in 80 mL chloroform and methylamine methanol (30%, w/v. 8 mL) was added, and stirred for 4 h at room temperature. After the reaction was completed, the solvent was evaporated under vacuum, and the residue was purified by silica gel column chromatograph using methanol/chloroform as the eluent to give compound **9** in 69.1% (343.3 mg). ^{1}H NMR (400 MHz, Chloroform-*d*) δ 9.19 (s, 1H), 8.41 (dt, *J* = 9.8, 5.4 Hz, 3H), 8.05 (d, *J* = 9.2 Hz, 1H), 7.59 (p, *J* = 5.9 Hz, 2H), 4.71 (d, *J* = 26.0 Hz, 4H), 4.02 (s, 3H), 2.68 (d, *J* = 5.8 Hz, 6H). ^{13}C NMR (101 MHz, $CDCl_3$) δ 167.51, 134.79, 132.24, 131.79, 131.40, 130.76, 129.19, 129.04, 127.05, 126.35, 125.48, 125.38, 125.10, 124.41, 52.53, 48.22, 48.05, 37.36. HRMS (ESI$^+$): calcd for $C_{20}H_{23}N_2O_2$ [M+H]$^+$ 323.1754, found 323.1752.

(((((2-(methoxycarbonyl)anthracene-9,10-diyl)bis(methylene))bis(methylazanediyl))bis(methylene)) bis(4-cyano-2,1-phenylene))diboronic acid Mc-CDBA: In a round-bottom flask, compound **9** (363 mg, 1.13 mmol) was mixed with compound **3** (0.87 g, 2.83 mmol), potassium carbonate (234 mg, 1.70 mmol),

potassium iodide (38 mg, 0.23 mmol) and 8 mL of DMF, and evacuated under nitrogen for three times. The reaction mixture was stirred at room temperature for 16 h. After that, the reaction mixture was diluted with 40 mL of chloroform, washed with saturated sodium chloride solution, and evaporated under vacuum. The solid was then dissolved in methanol and water was added dropwise with stirring until light-yellow precipitate was observed. The yellow solid was filtrated and re-precipitated with DCM-ether, to give **Mc-CDBA** in 47.7% yield (0.52 g). ^{1}H NMR (800 MHz, Pyridine-d_5) δ 9.40 (s, 1H), 8.67 – 8.50 (m, 3H), 8.43 – 8.28 (m, 3H), 7.88 – 7.63 (m, 6H), 4.65 (d, J = 41.2 Hz, 4H), 4.09 – 4.00 (m, 7H), 2.20 (d, J = 19.4 Hz, 6H). ^{13}C NMR (201 MHz, Pyr) δ 167.78, 150.69, 144.05, 143.93, 137.17, 137.01, 136.37, 134.46, 134.39, 133.34, 133.00, 132.46, 131.39, 131.31, 131.00, 130.67, 130.16, 127.80, 127.58, 127.17, 126.96, 126.69, 126.61, 124.98, 124.33, 120.05, 120.00, 113.64, 113.62, 64.47, 64.26, 52.98, 52.80, 52.58, 42.31, 42.11. HRMS (ESI$^+$): calcd for $C_{40}H_{43}B_2N_4O_6$ $[M+4CH_3OH–4H_2O+H]^+$ 697.3363, found 697.3368.

9,10-bis(((2-borono-5-cyanobenzyl)(methyl)amino)methyl)anthracene-2-carboxylic acid Ca-CDBA: In a round-bottom flask, compound **Mc-CDBA** (0.50 g, 0.78 mmol) was mixed with 15 mL of methanol and 5 mL of 3 N sodium hydroxide aqueous solution, and stirred at 70 °C for 5 h. After that, the mixture was treated with prewashed cation exchange resin to remove alkaline. The mixture was evaporated under vacuum to give compound **Ca-CDBA** as a yellow powder in 67.2% yield (328.7 mg). ^{1}H NMR (800 MHz, Pyridine-d_5) δ 9.59 (dd, J = 36.5, 11.5 Hz, 1H), 9.10 (d, J = 27.8 Hz, 1H), 8.65 – 8.38 (m, 9H), 7.87 – 7.73 (m, 2H), 4.75 – 4.46 (m, 4H), 4.21 – 3.95 (m, 4H), 2.20 (d, J = 14.1 Hz, 6H). (Mixture of diastereomer) ^{13}C NMR (201 MHz, Pyr) δ 170.44, 170.43, 170.40, 170.39, 150.48, 144.10, 143.95, 143.05, 142.91, 137.44, 137.36, 137.13, 137.09, 136.95, 134.53, 134.46, 133.17, 133.15, 133.11, 133.08, 133.03, 133.02, 132.95, 132.41, 132.37, 132.32, 132.28, 131.68, 131.41, 131.31, 131.06, 131.03, 131.01, 130.94, 130.85, 130.81, 130.73, 130.08, 130.04, 130.02, 129.97, 129.75, 129.73, 129.69, 129.66, 127.56, 127.54, 127.38, 127.34, 127.31, 127.27, 126.91, 126.81, 126.77, 126.74, 126.70, 126.64, 126.52, 125.88, 125.82, 125.79, 125.73, 120.10, 120.03, 113.65, 113.57, 65.74, 65.71, 65.55, 65.52, 64.58, 64.56, 64.28, 64.26, 52.87, 52.62, 52.60, 52.33, 52.11, 52.08, 42.51, 42.35, 42.17, 42.10. HRMS (ESI$^+$): calcd for $C_{39}H_{41}B_2N_4O_6$ $[M+4CH_3OH–4H_2O+H]^+$ 683.3207, found 683.3213.

General procedures of fluorescence detection

Stock solutions were prepared by dissolving 2 mM **Mc-CDBA** and **Ca-CDBA** probes in methanol and DMSO, respectively, and stored at 4 °C until use. Before spectroscopic tests, the probe solution was freshly prepared by diluting the stock solution to 20 μM probe solution, then the probe solution was mixed with equal volume of sample solution to record the fluorescence spectra. The glucose detection assay in DMEM used the DMEM (without glucose) from GIBCO® DMEM medium (#11966025, Thermo Fisher Scientific).

Quantum yield (Φfl) measurement

Fluorescence quantum yield (Φfl) of the probes was measured using coumarin-1 (Φfl = 0.58 in DMSO) as standard at an excitation wavelength of 370 nm and recorded emission spectra from 380 to 600 nm. The fluorescence quantum yield (Φfl) was calculated according to the following equation:

$$\Phi_{\text{probe}} = \Phi_{\text{std}} \frac{F_{\text{probe}}\, \eta_{\text{probe}}\, Abs_{\text{std}}}{F_{\text{std}}\, \eta_{\text{std}}\, Abs_{\text{probe}}} \ ,$$

where Φ_{probe} and Φ_{ref} represent the fluorescence quantum yield of the probe and standard, F_{probe} and F_{std} are the calculated area under the fluorescent emission profile of the probe and standard, η_{probe} and η_{std} are the reflective index of the probe and standard solvent, and Abs_{probe} and Abs_{std} are the absorbance of the probe and standard.

Cell line and cell culture

HeLa, Caco-2, FHC, HepG2 and L-02 cells were obtained from the Institute of Cell Biology (Shanghai, China). HeLa, FHC and HepG2 cells were cultured in DMEM supplemented with 10% FBS, 1% penicillin, 1% streptomycin sulfate in a 5% CO_2 incubator at 37 °C. L-02 cells were maintained in RPMI 1640 medium supported with 10% FBS, 1% penicillin, 1% streptomycin sulfate in a 5% CO_2 incubator at 37 °C. Caco-2 cells were cultured in DMEM supplemented with 15% FBS, 1% penicillin, 1% streptomycin sulfate in a 5% CO_2 incubator at 37 °C. One day before imaging, the cells were isolated and placed in glass-bottomed confocal dishes.

Cytotoxicity assay

MTT assay was performed to determine the cytotoxicity of **Mc-CDBA** and **Ca-CDBA** towards HeLa, HepG2 and L-02 cells. The well-growing cells were washed with PBS for 2−3 times and digested with trypsin to get cell suspension, counted, and diluted so that the cell density was about 10^6 cells/mL. The diluted cell suspension was placed in a 96-well plate with 200 μL per well and cultured in a cell incubator. After 24 h, the adherent state of the cells was observed under the microscope. If the cells grew well, the original culture medium in the orifice plate was abandoned, and 200 μL medium containing different concentrations of probes (0, 10, 20, 40, 50, 60, 80, 100 μM) was added. After 24 hours, the culture medium containing probe was discarded and the MTT (3-(4,5-dimethylthiazol-2-yl)-2,5-diphenyltetrazolium bromide) of 5 mg/mL was added with 200 μL per well under dark, and further cultured in the cell incubator. After 4 hours, the supernatant was discarded and 200 μL of DMSO was added to each hole to avoid light and shake for 10 min to completely dissolve the formazan product. Finally, the absorbance value at 570 nm was measured by microplate reader.

Cell uptake assay

Stock solutions were prepared by dissolving 2 mM **Mc-CDBA** and **Ca-CDBA** probes in methanol and DMSO, respectively, and stored at 4 °C until use. Before cell tests, the probe solution was freshly prepared by diluting the stock solution with DMEM (without glucose) to 50 μM probe solution. The glucose detection assay used DMEM (without glucose) from GIBCO® DMEM medium (#11966025, Thermo Fisher Scientific). HeLa cells were subcultured into glass-bottomed confocal dishes, and when the cells had grown to 80% of the area, the cells were incubated with a medium of 50 μM probe concentration. Cell images were acquired by confocal microscopy (Leica TCS SP8, λ_{ex}: 405 nm, λ_{em}: 410−600 nm) at 0, 5, 10, 15, 20, 25, 30, 35, 40 min, respectively. Images were processed using Image J software, and then the incubation time of the probes were determined based on the fluorescence intensity results.

Cell starvation experiment

HeLa cells in good growth state were passaged into confocal dishes, keeping the same cell density in each dish and cultured overnight. After 24 h, HeLa cells were cultured in glucose-free DMEM medium for 0,

4, 8, 12, 24 h, respectively. Then, cells were washed three times with PBS, and then 1 mL of PBS was added for imaging (Leica TCS SP8, λ_{ex}: 405 nm, λ_{em}: 410−600 nm).

Flow cytometric imaging of normal and tumor cells

HepG2 and L-02 cells with good growth conditions were subcultured into 6-well plates, and those two kinds of cells were kept at the same density and cultured overnight. HepG2 and L-02 cells were incubated with probe **Mc-CDBA** for 30 min. At the end of incubation, the culture medium was removed, and the cells were washed with PBS for 3 times, digested with trypsin, washed with PBS for 3 times again, and then transferred for flow cytometer analysis. The excitation wavelength of 405 nm was selected and using the fluorescence signal of Pacific Blue-A channel for the test. The flow cytometry experiments of Caco-2 and FHC cells were consistent with those of HepG2 and L-02 cells.

Fish maintenance and embryo collection

Adult zebrafish (Danio rerio, AB strain) used in this work were maintained in a recirculating culture system (ESEN, Beijing, China) after being purchased from Fish Bio Co., Ltd. (Shanghai, China). The culture conditions of adult zebrafish were designed at approximately 28 °C with a 14/10 h light/dark photoperiod, and fresh fairy shrimp (Artemia salina) nauplii were provided to zebrafish twice daily. For mating, the adults were paired in a commercial incubator (23 cm × 12 cm × 12 cm) overnight, and spawning commenced the following morning. Collected fertilized eggs were rinsed with embryonic culture medium (E3 medium, 5 mM NaCl, 0.17 mM KCl, 0.33 mM $CaCl_2$, 0.33 mM $MgSO_4$, pH 7.4) and were used for subsequent experiments.

Zebrafish uptake tests

7 dpf zebrafish embryos were administrated with 50 μM **Mc-CDBA** or **Ca-CDBA** and incubated continuously for 0.5, 1, 2, and 3 h, respectively. After incubation, embryos were anesthetized with 0.02% tricaine for 1 min and the fluorescence images was detected by confocal microscope (Leica TCS SP8, λ_{ex}: 405 nm, λ_{em}: 410−600 nm). The fluorescence intensity of zebrafish embryos was quantified by Image J software.

Fluorescence imagining tests of 1-10 dpf zebrafish

1−10 dpf zebrafish embryos were administrated with 50 μM **Mc-CDBA** or **Ca-CDBA** and incubated continuously for 3 h. At the end of incubation, embryos were anesthetized with 0.02% tricaine for 1 min and the fluorescence images was detected by confocal microscope (Leica TCS SP8, λ_{ex}: 405 nm, λ_{em}: 410−600 nm). The fluorescence intensity of zebrafish embryos was quantified by Image J software.

Drug efficacy evaluation tests

Three groups were set up for each probe. In brief, the first and second group of zebrafish embryos were firstly incubated in culture medium with 10 mM glucose for 4 h, and the third group of zebrafish embryos were incubated in culture medium with 10 mM glucose and 20 μM AMPK or 20 mM 4,6-EDG for 4 h. Then, the second and third group were incubated in culture medium with 50 μM **Mc-CDBA** or **Ca-CDBA**, respectively, for 1 h. After incubation, embryos were anesthetized with 0.02% tricaine for 1 min and the fluorescence intensity was detected by confocal microscope (Leica TCS SP8, λ_{ex}: 405 nm, λ_{em}: 410−600 nm). The fluorescence intensity of zebrafish embryos was quantified by Image J software.

Data analysis

Data are presented as the means ± SD (n = 3) unless otherwise indicated in the figure legend. Statistical analysis was carried out using GraphPad Prism v6.01. The statistical comparison of results was performed using one- or two-way ANOVA with Tukey's honest significant difference test correction for multiple comparisons, with levels of significance set at n.s. (no significant difference), * $P < 0.05$, ** $P < 0.01$, *** $P < 0.001$, and **** $P < 0.0001$.

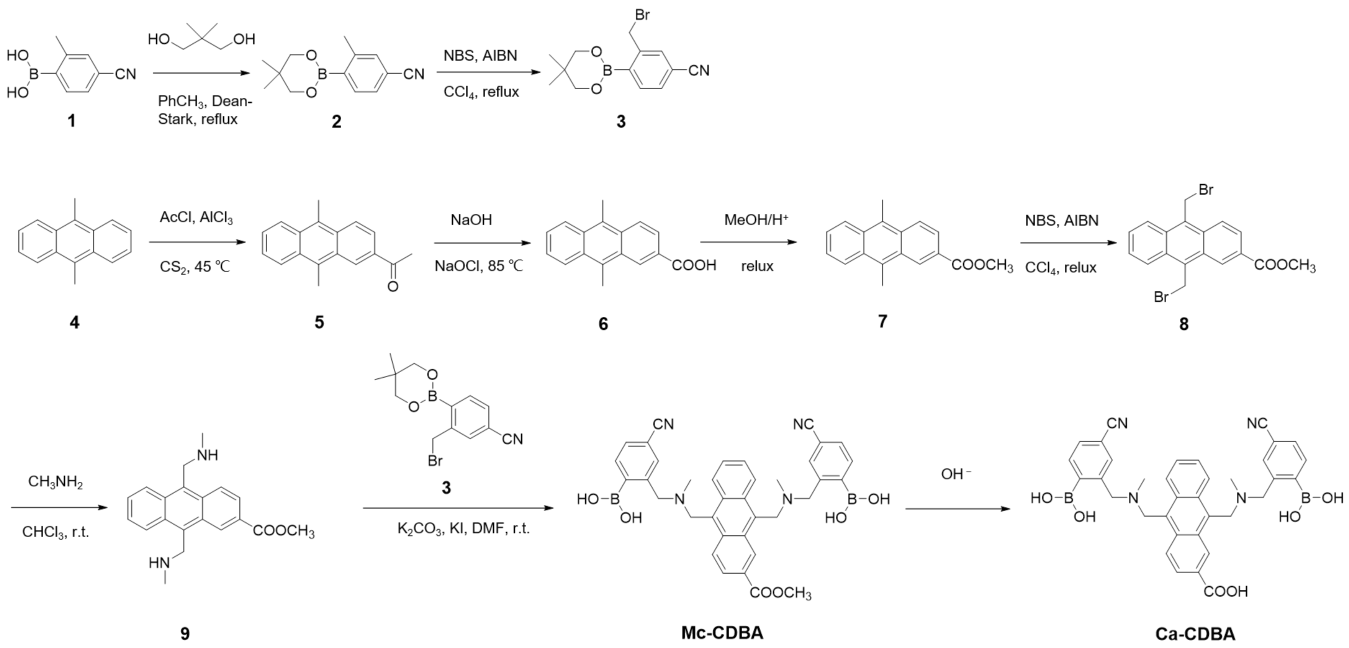

Supplementary Figure 1. Synthetic routes for **Mc-CDBA** and **Ca-CDBA**.

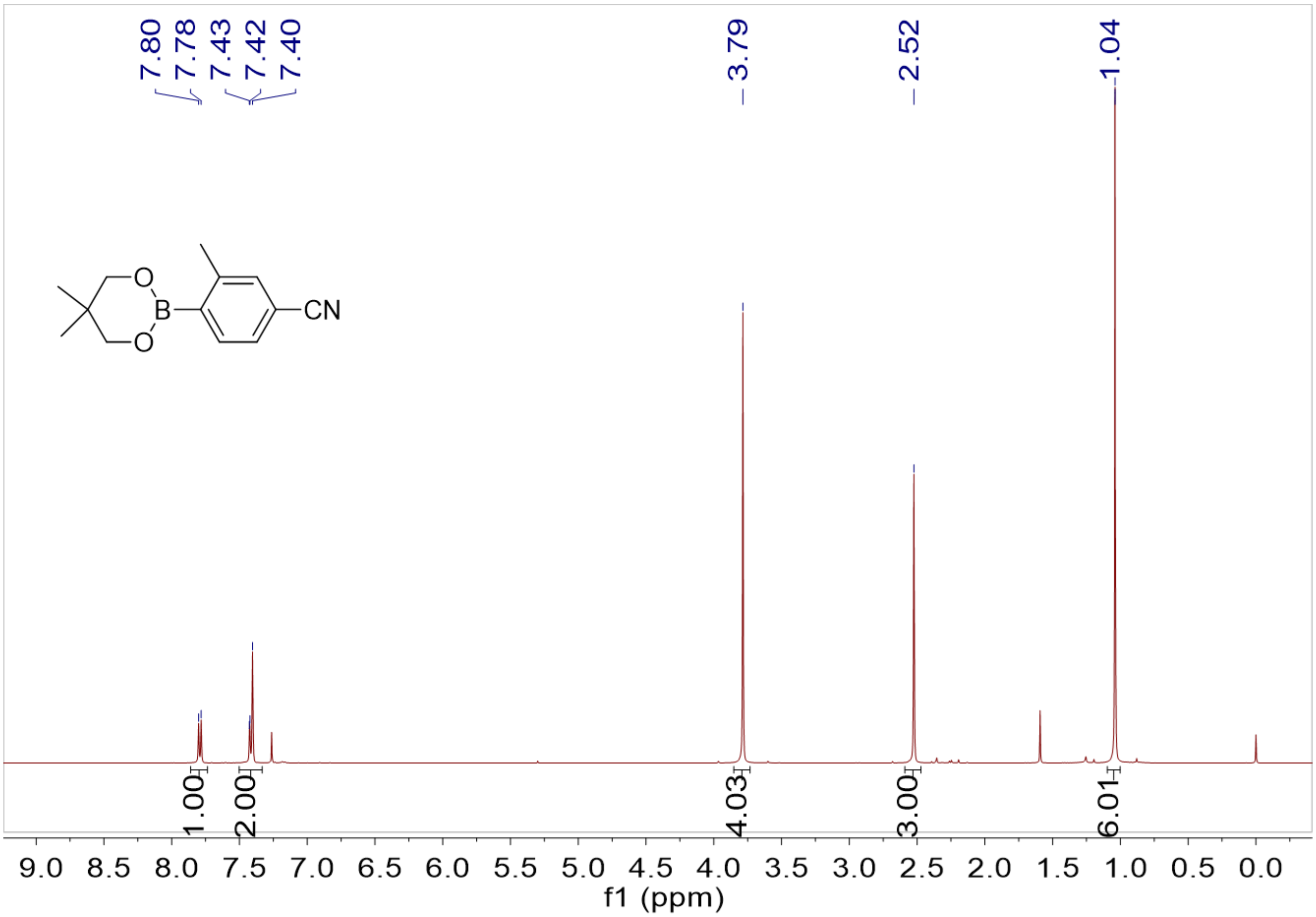

Supplementary Figure 2. ^{1}H NMR spectrum of compound **2**.

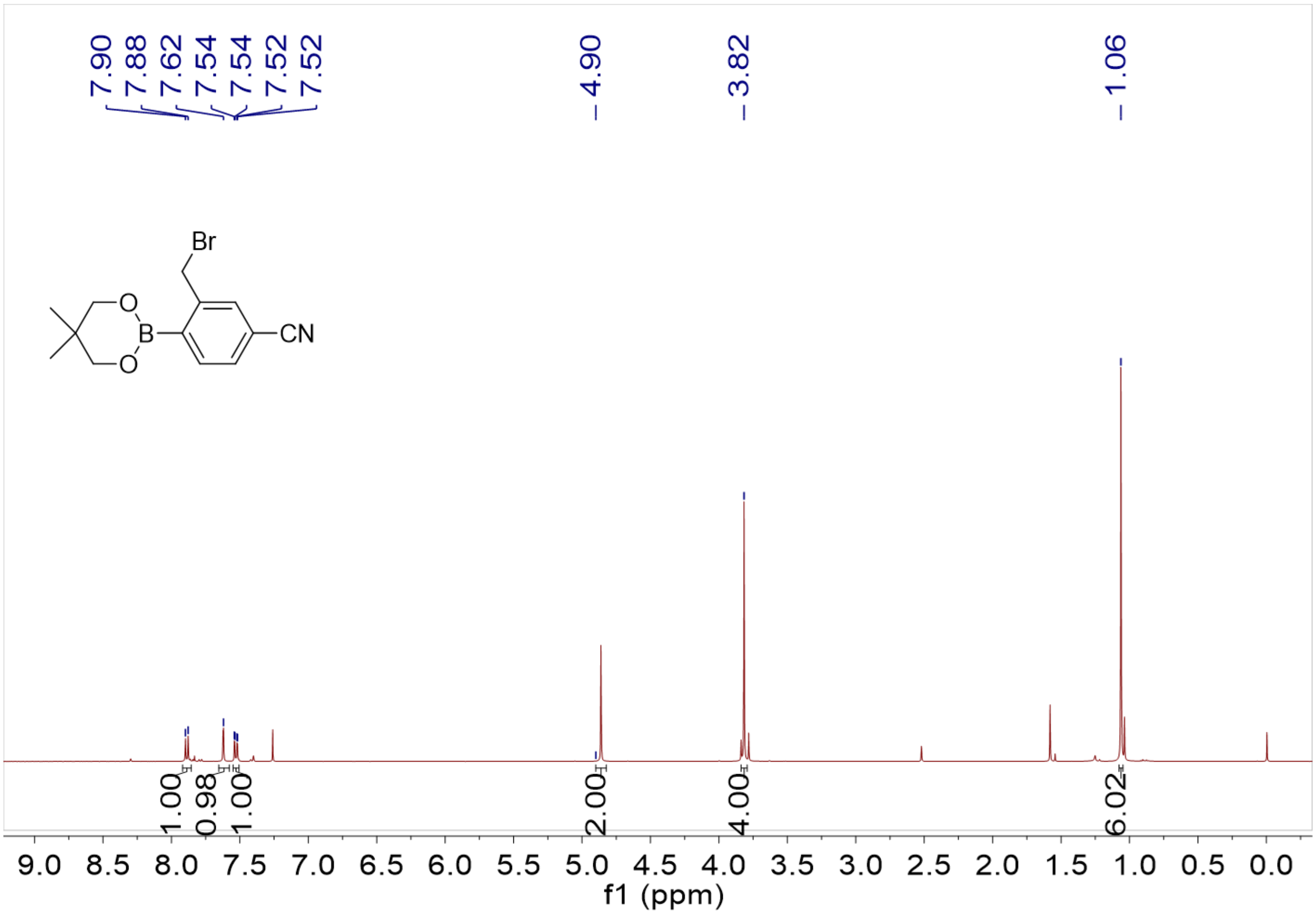

Supplementary Figure 3. ^{1}H NMR spectrum of compound **3**.

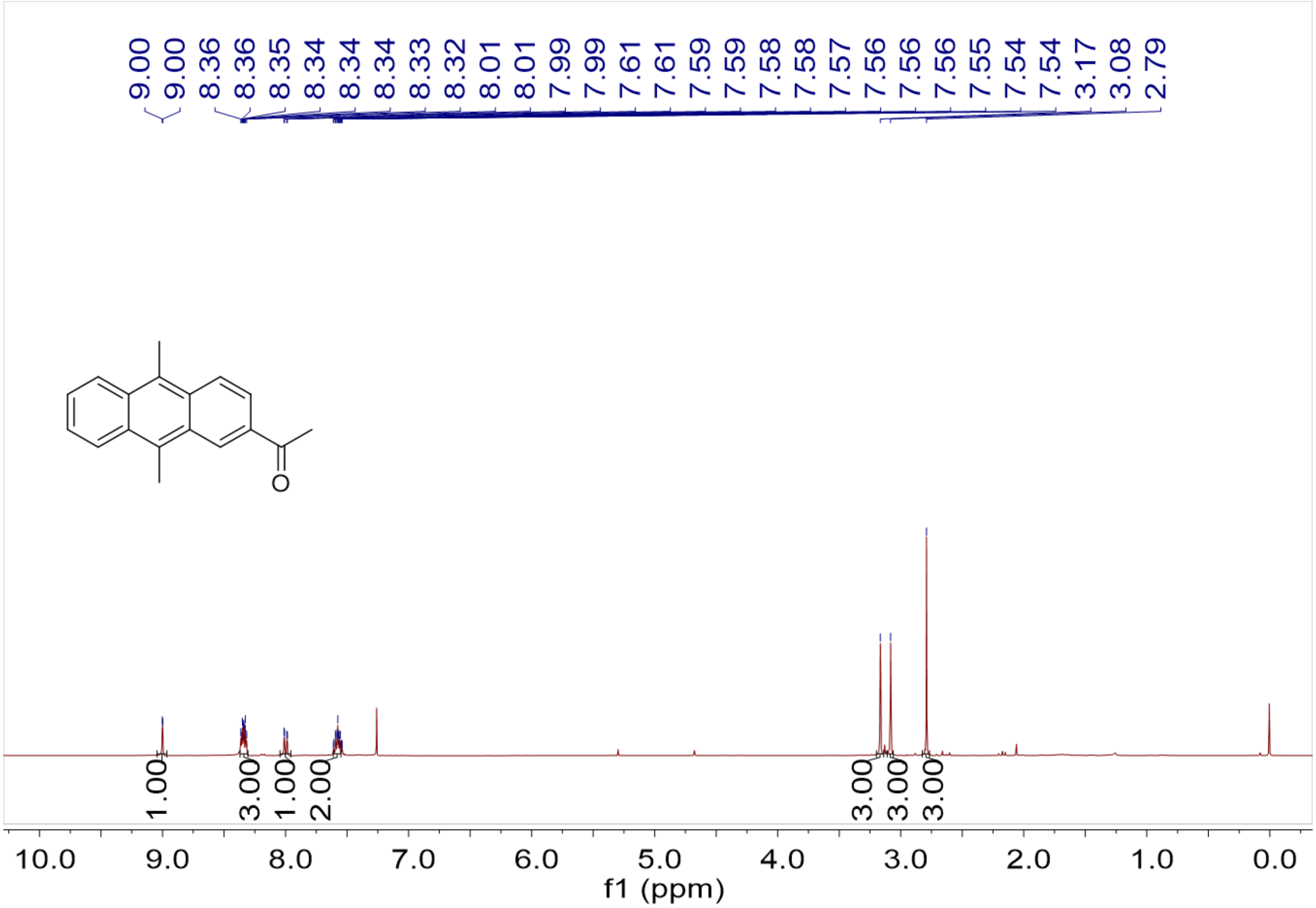

Supplementary Figure 4. ^{1}H NMR spectrum of compound **5**.

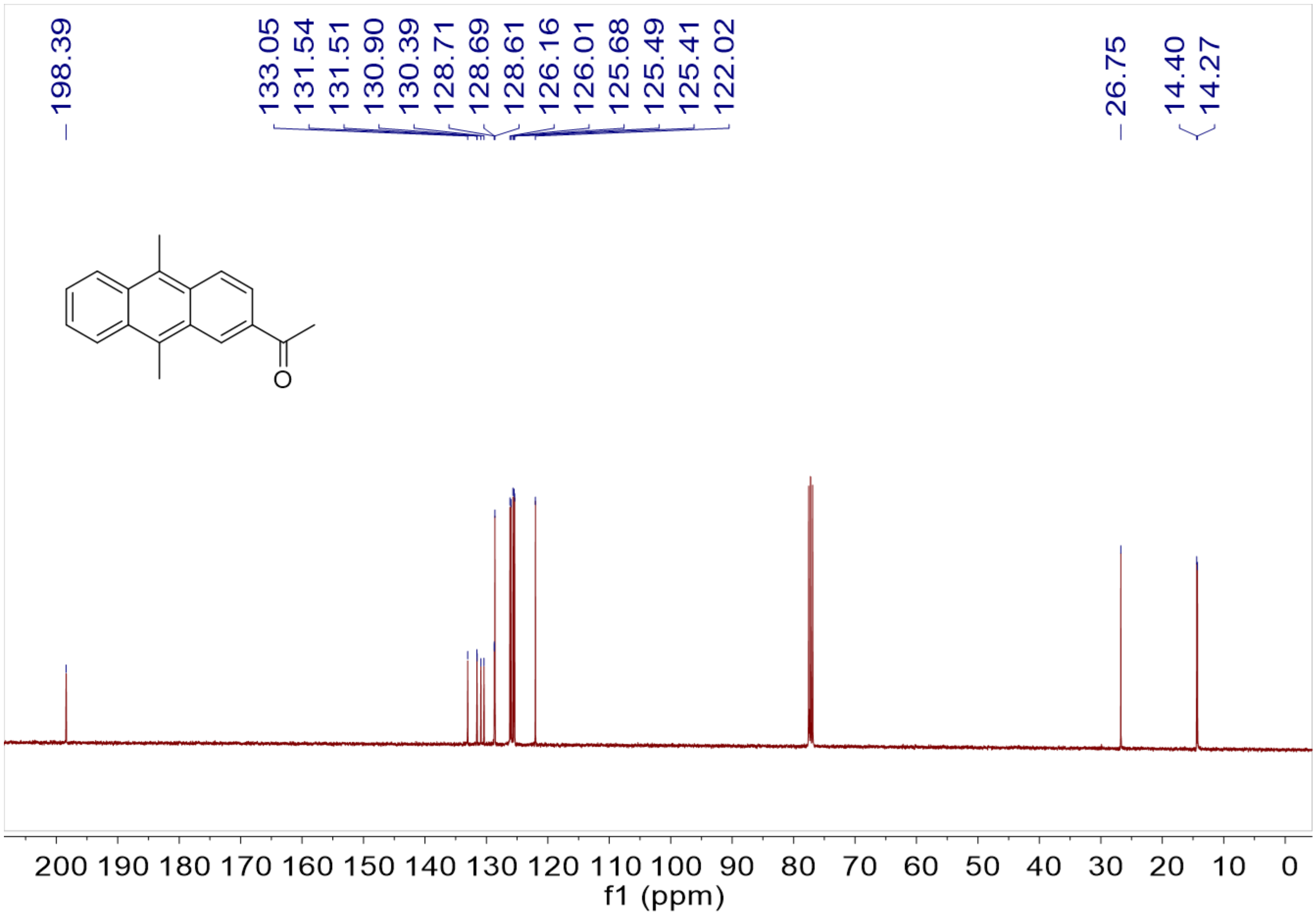

Supplementary Figure 5. ^{13}C NMR spectrum of compound **5**.

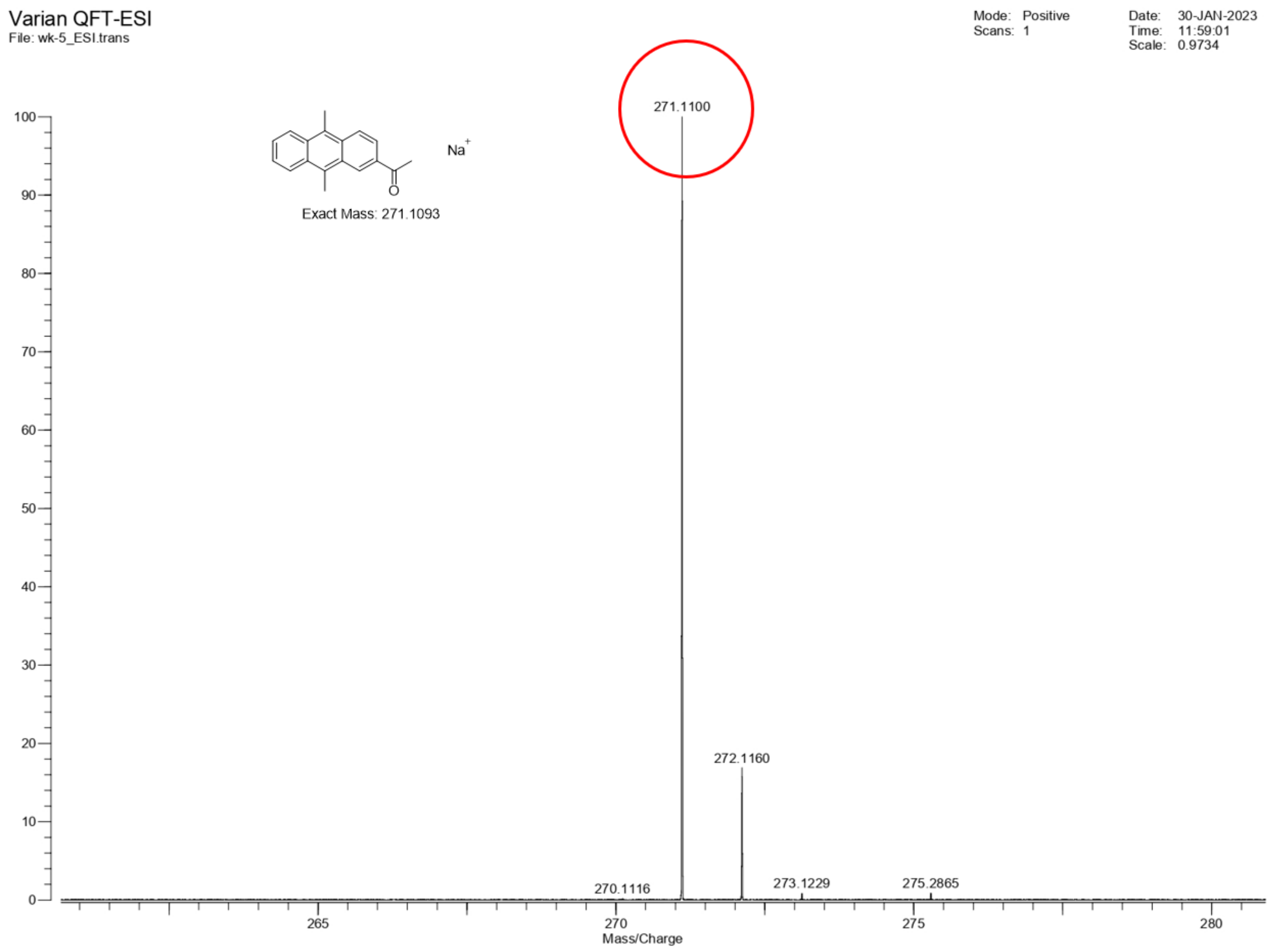

Supplementary Figure 6. High resolution mass spectrum (HR-MS) of compound **5**.

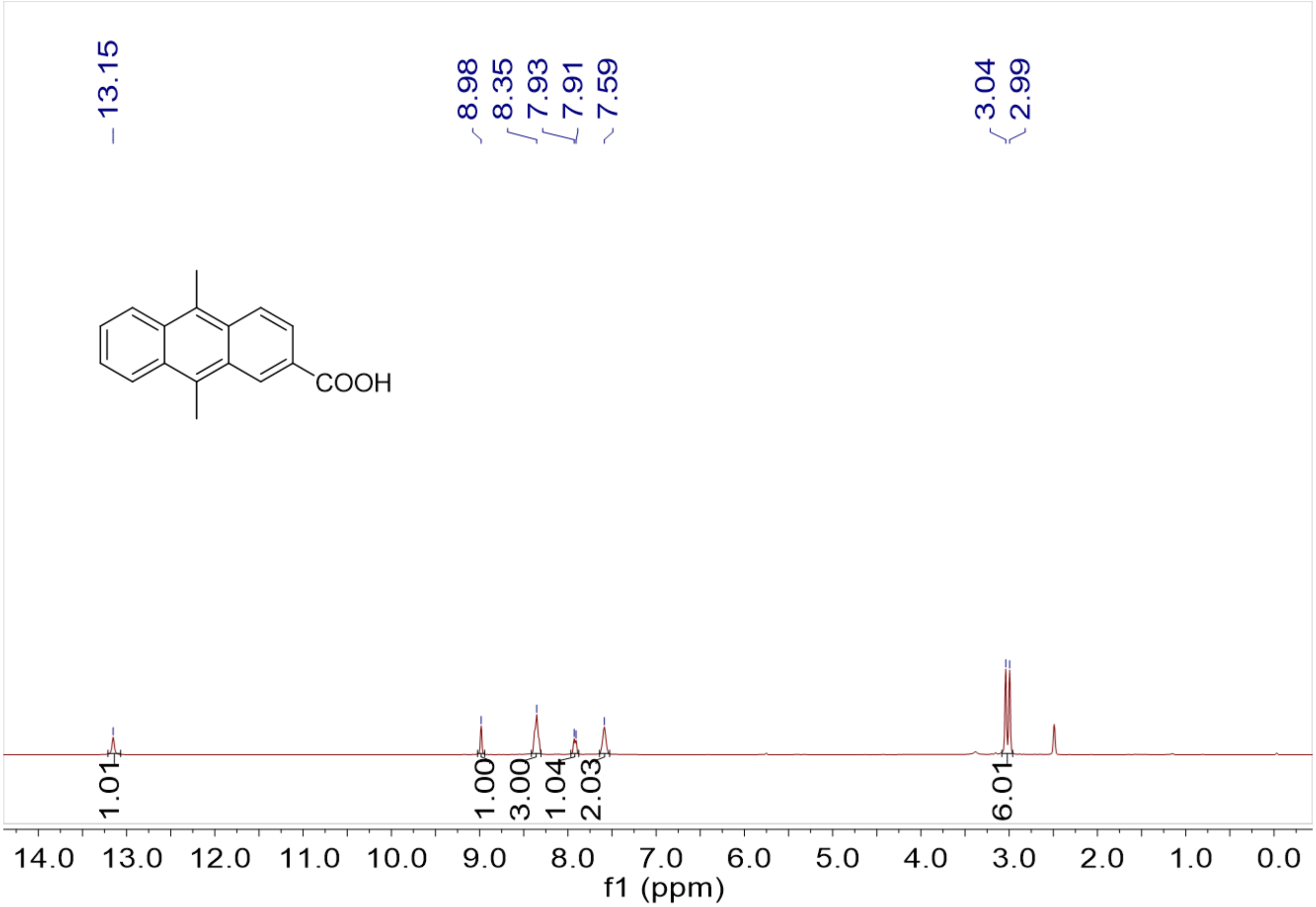

Supplementary Figure 7. ^{1}H NMR spectrum of compound **6**.

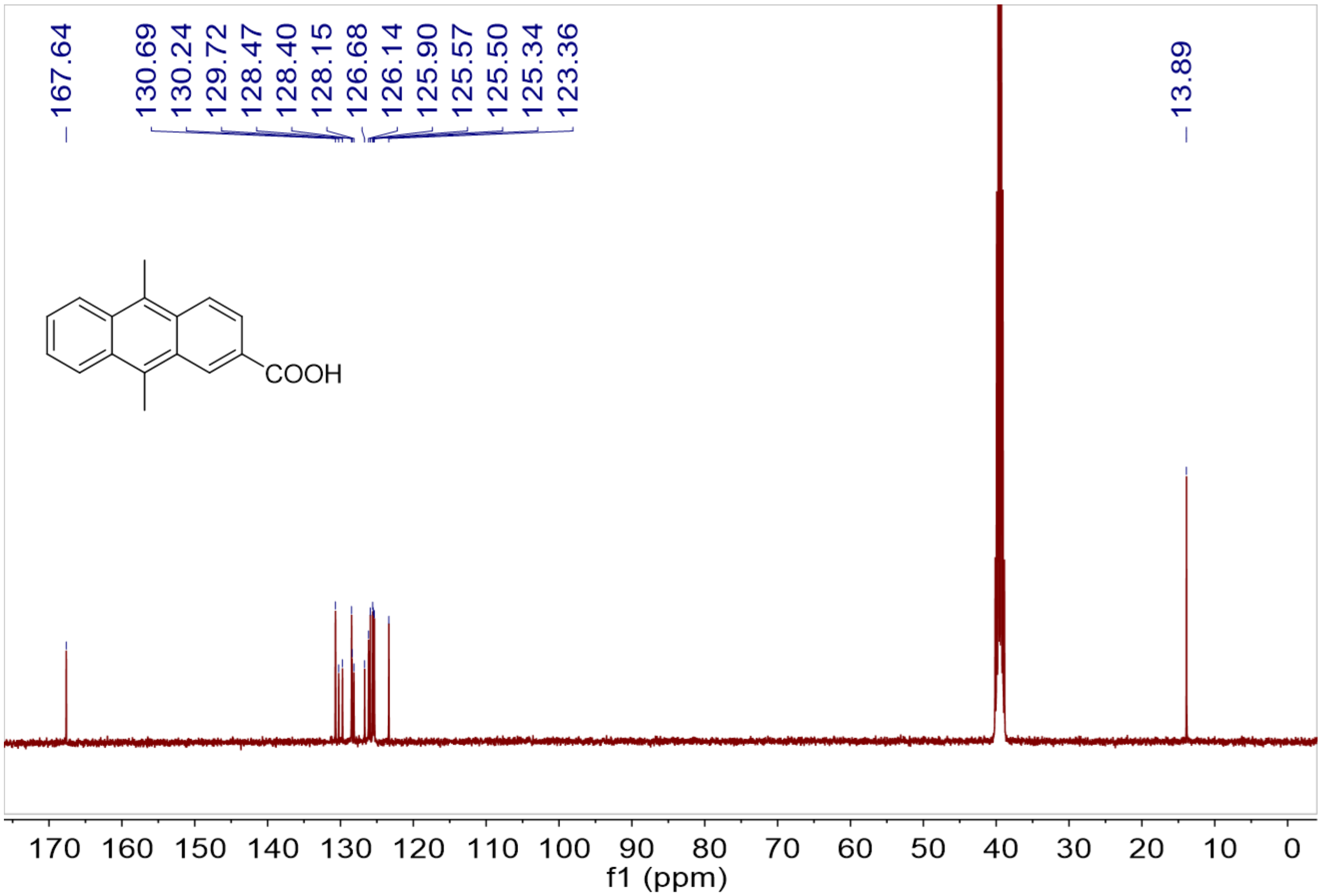

Supplementary Figure 8. ^{13}C NMR spectrum of compound **6**.

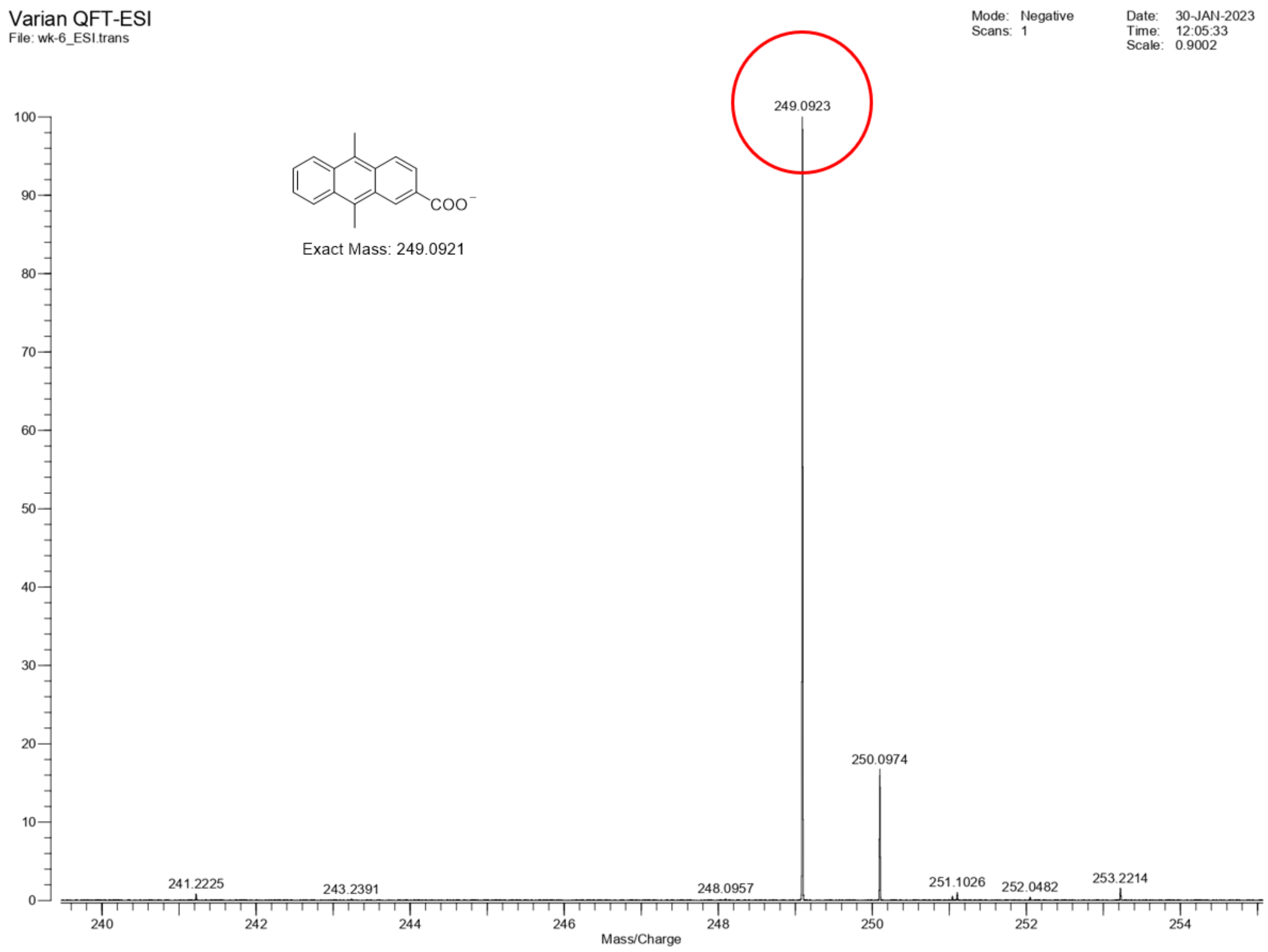

Supplementary Figure 9. High resolution mass spectrum (HR-MS) of compound **6**.

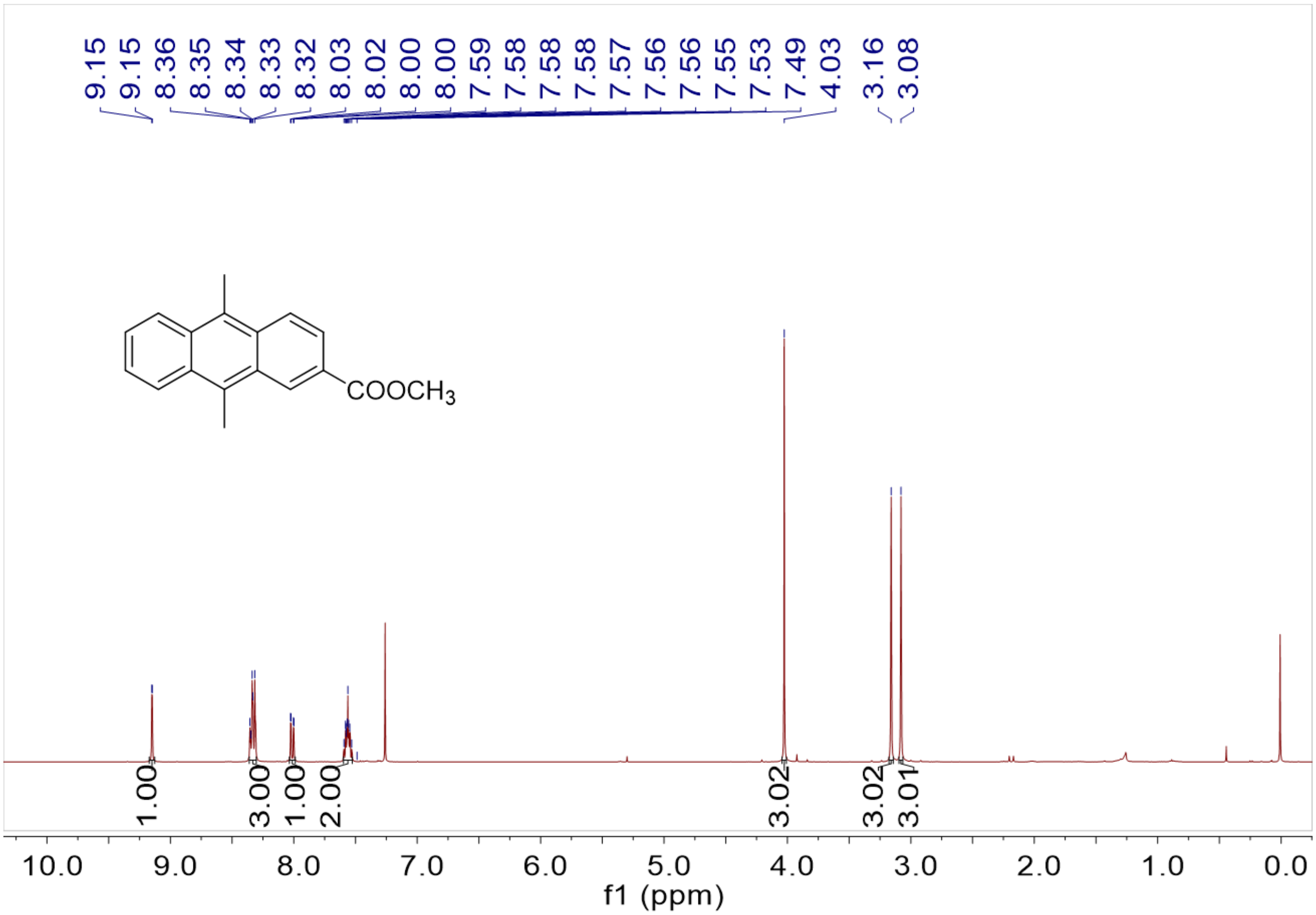

Supplementary Figure 10. ^{1}H NMR spectrum of compound **7**.

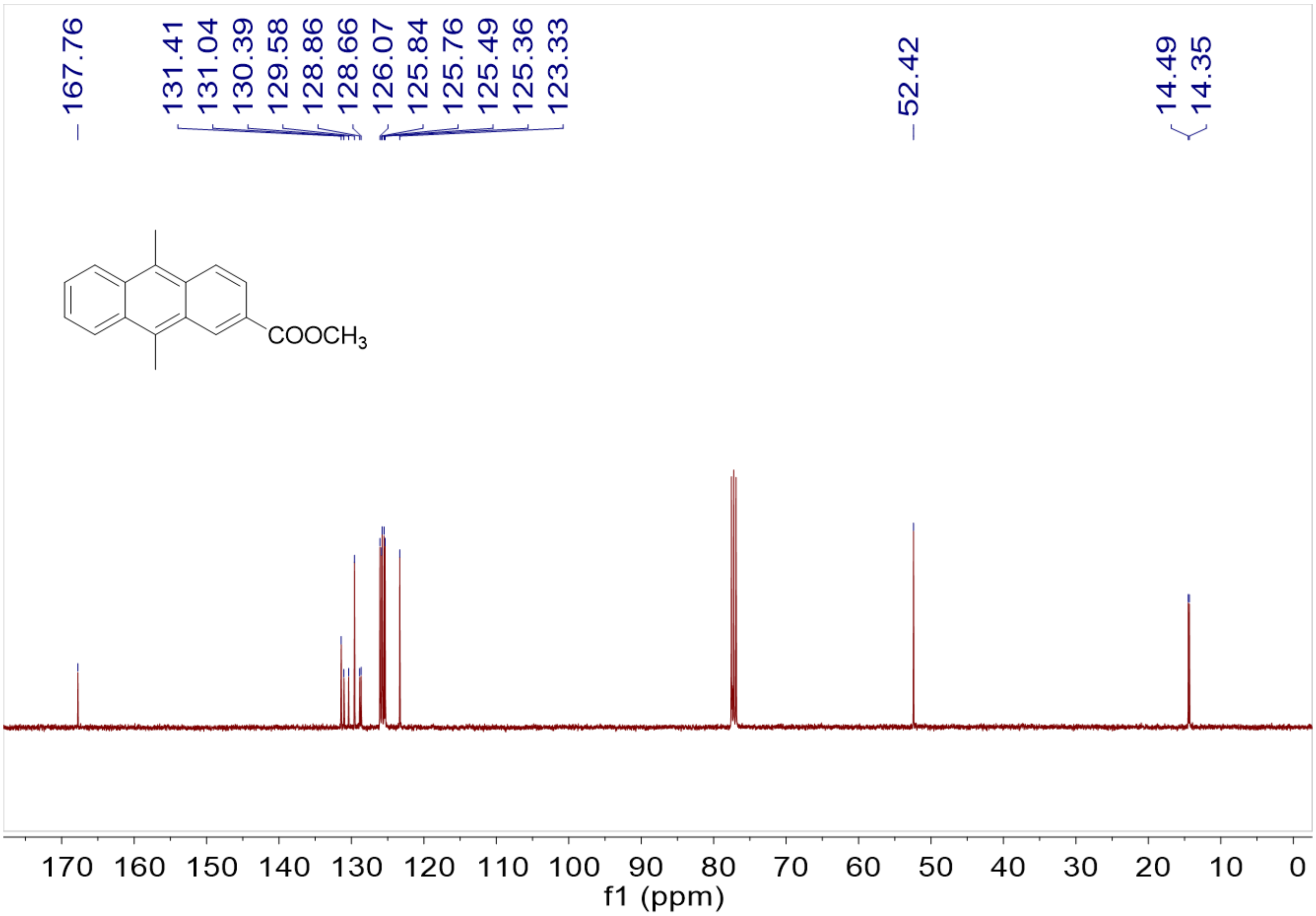

Supplementary Figure 11. ^{13}C NMR spectrum of compound **7**.

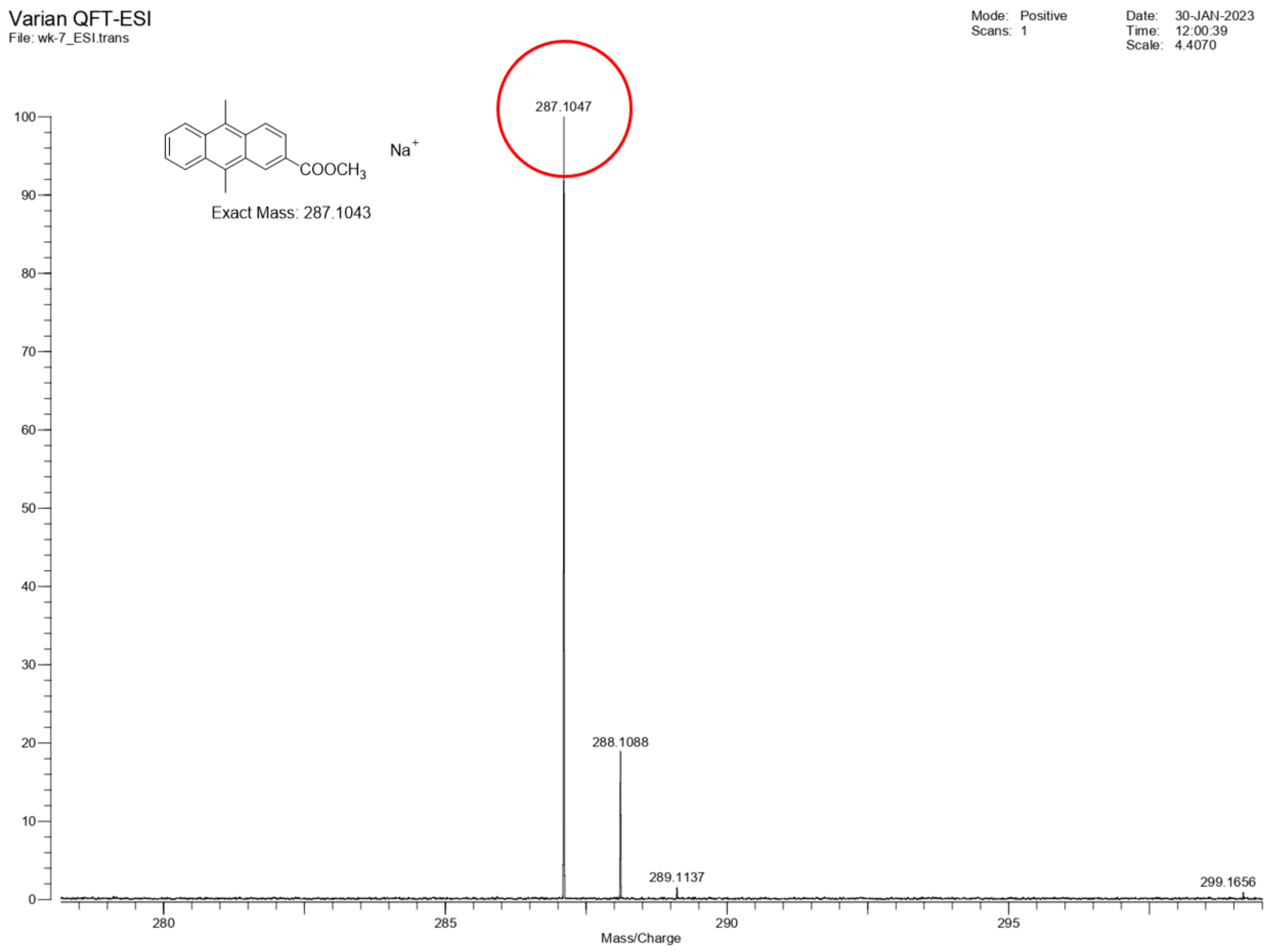

Supplementary Figure 12. High resolution mass spectrum (HR-MS) of compound **7**.

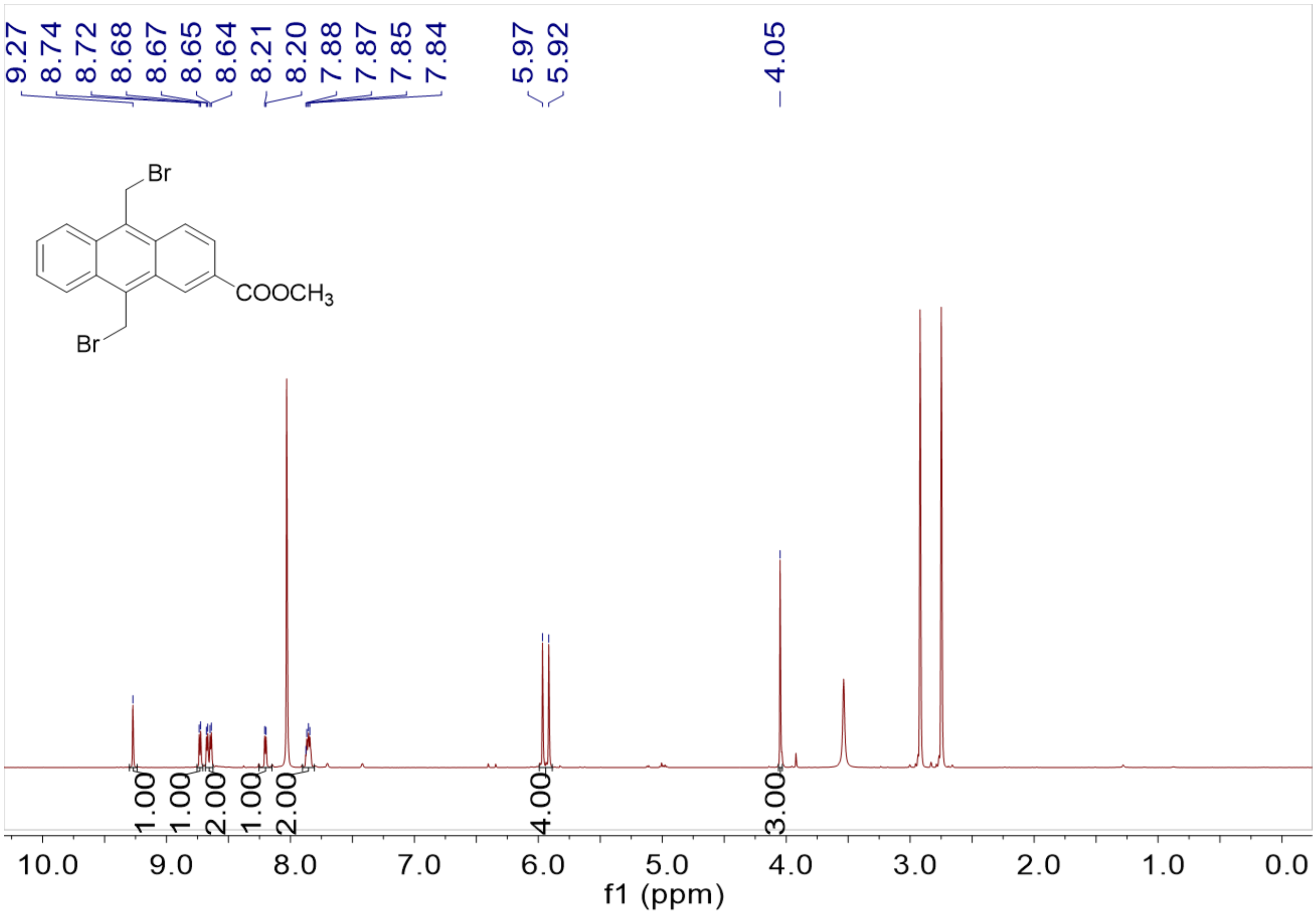

Supplementary Figure 13. ^{1}H NMR spectrum of compound **8.**

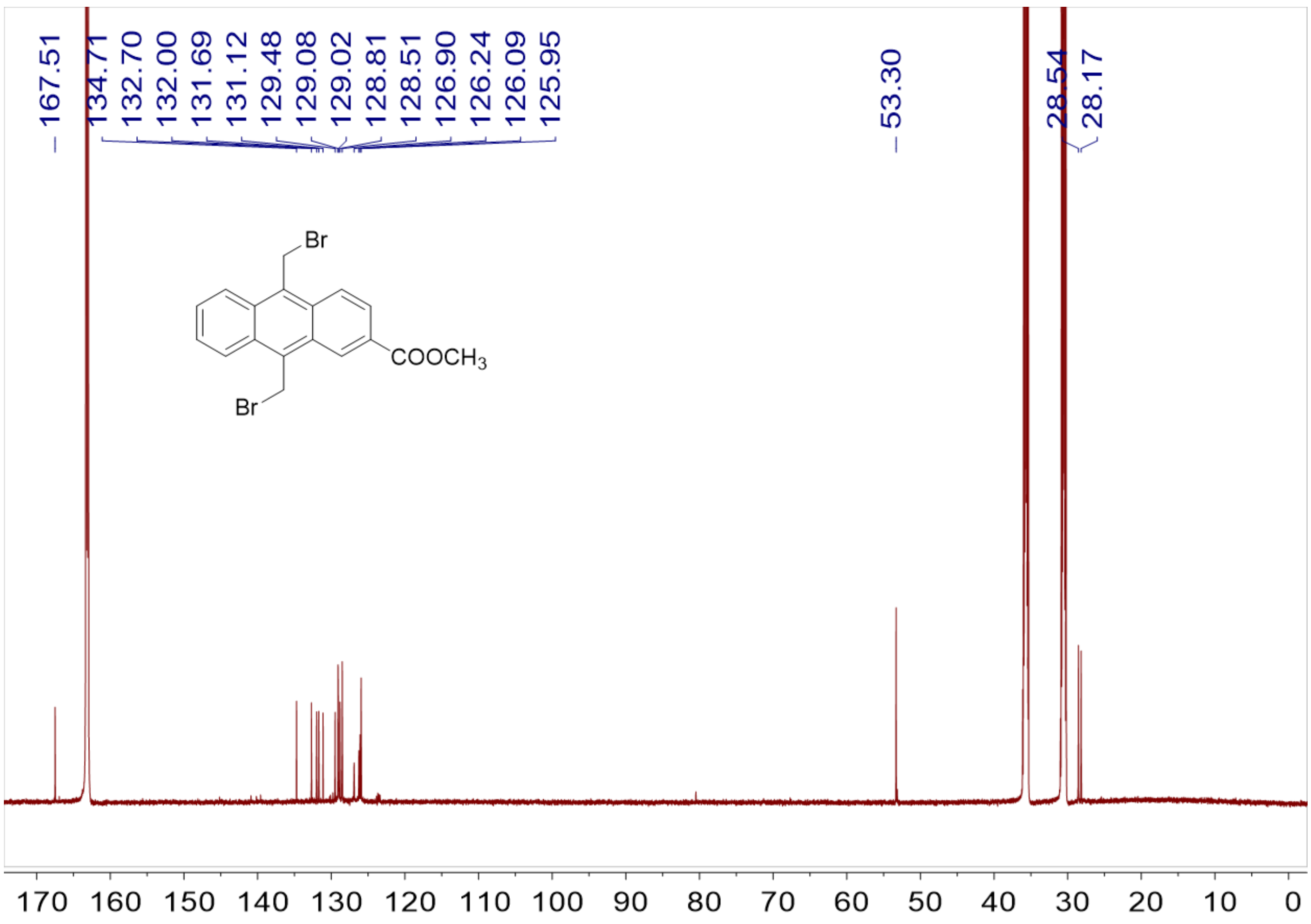

Supplementary Figure 14. ^{13}C NMR spectrum of compound **8**.

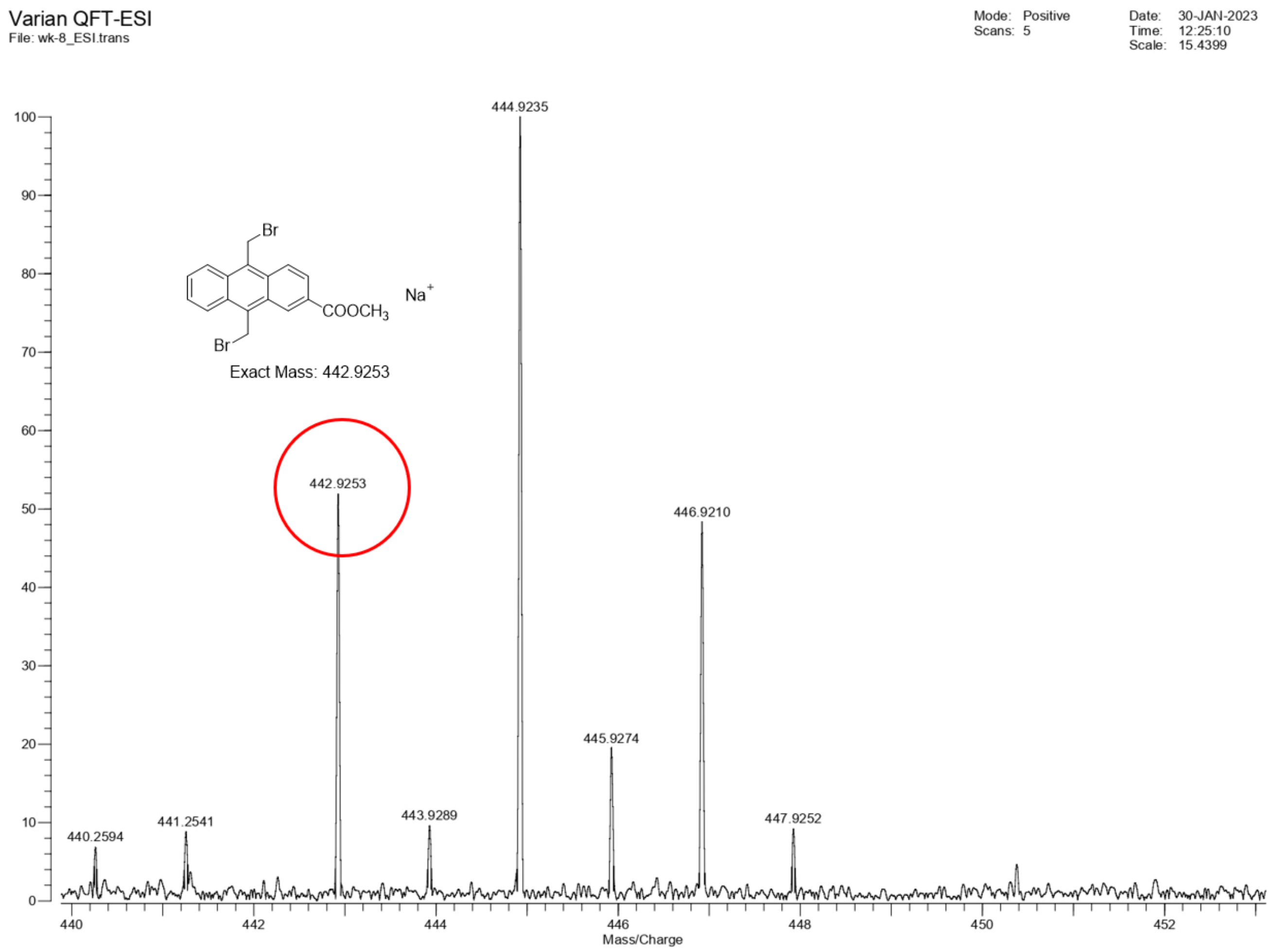

Supplementary Figure 15. High resolution mass spectrum (HR-MS) of compound **8**.

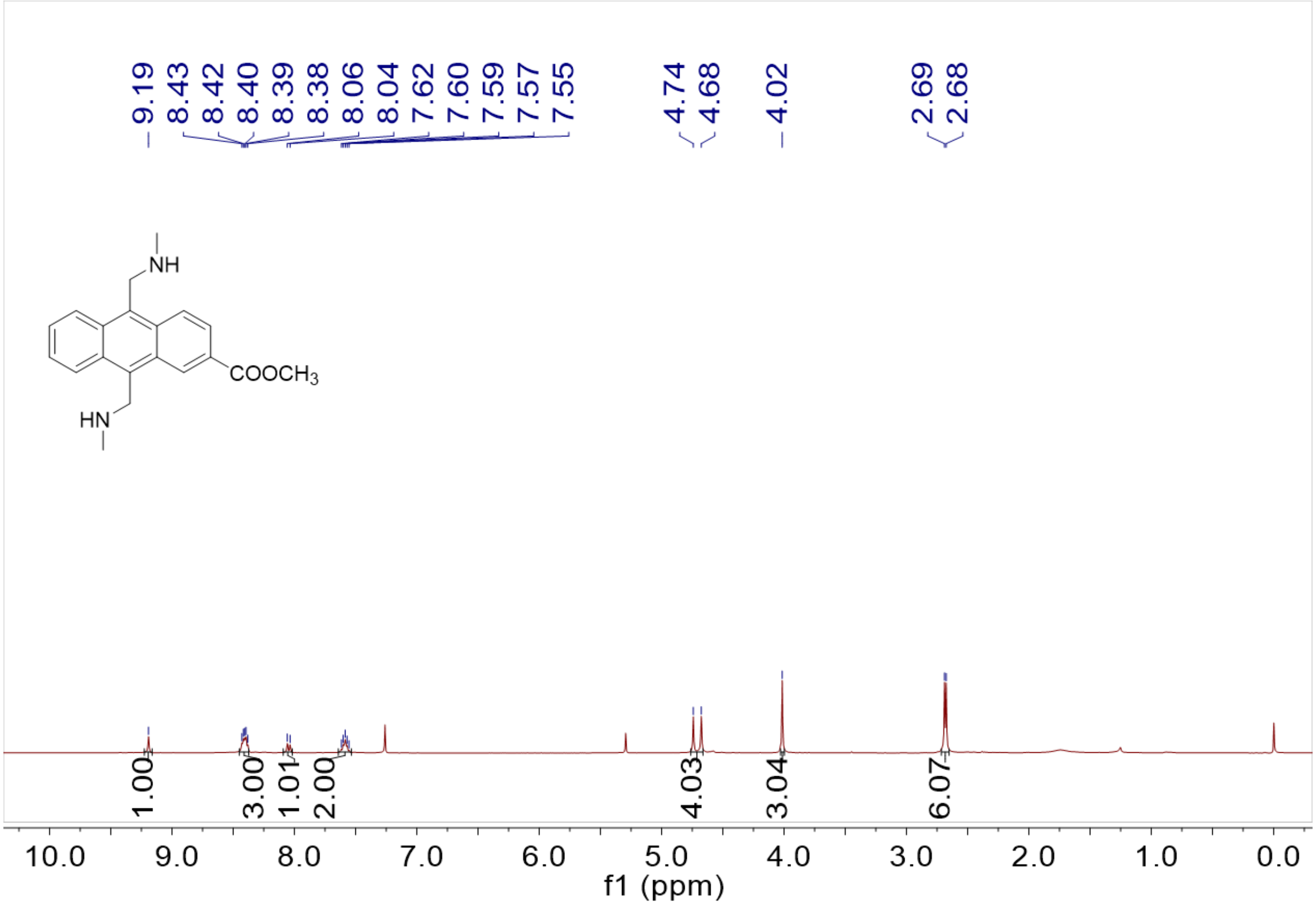

Supplementary Figure 16. ^{1}H NMR spectrum of compound **9**.

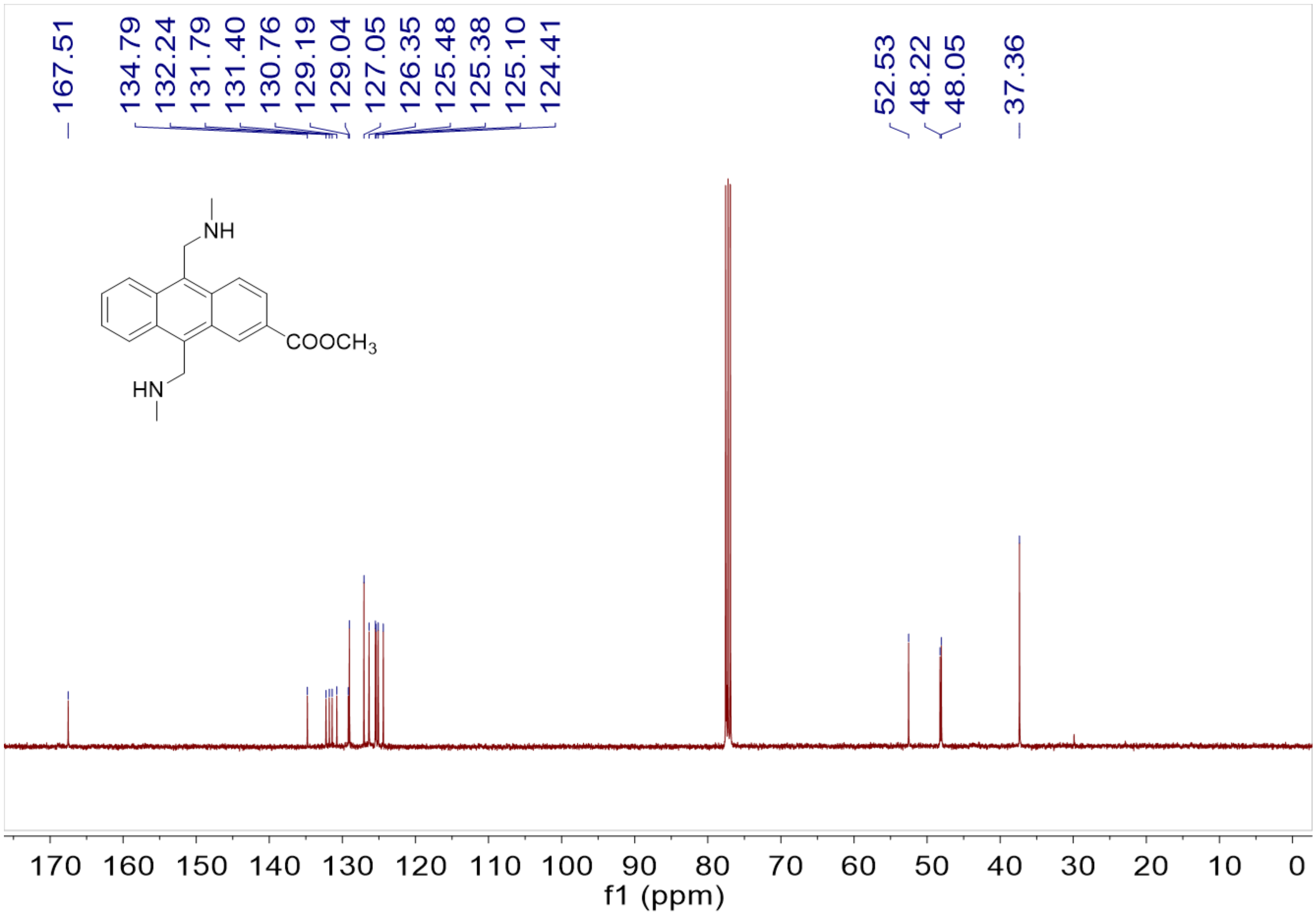

Supplementary Figure 17. ^{13}C NMR spectrum of compound **9**.

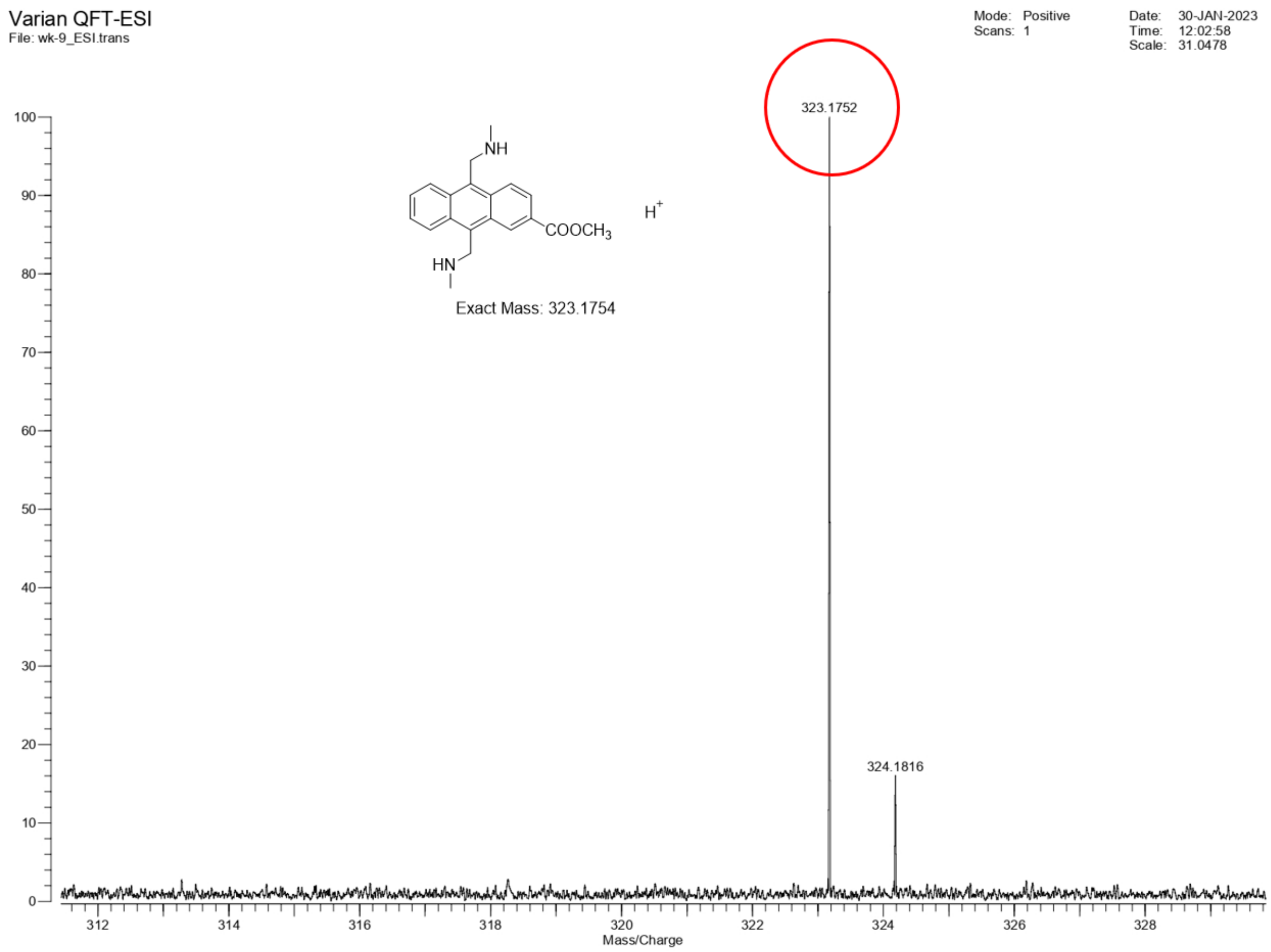

Supplementary Figure 18. High resolution mass spectrum (HR-MS) of compound **9**.

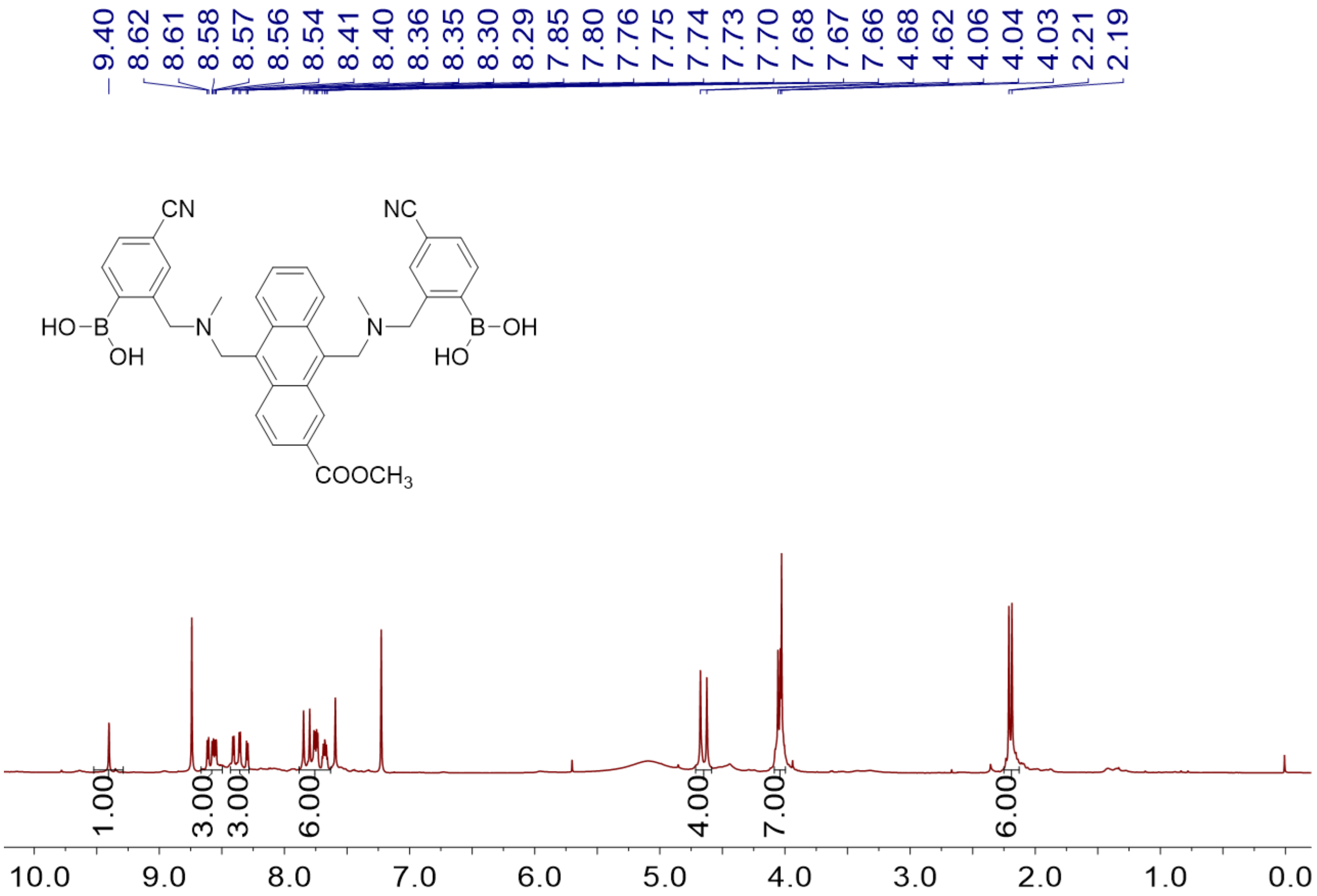

Supplementary Figure 19. ^{1}H NMR spectrum of compound **Mc-CDBA**.

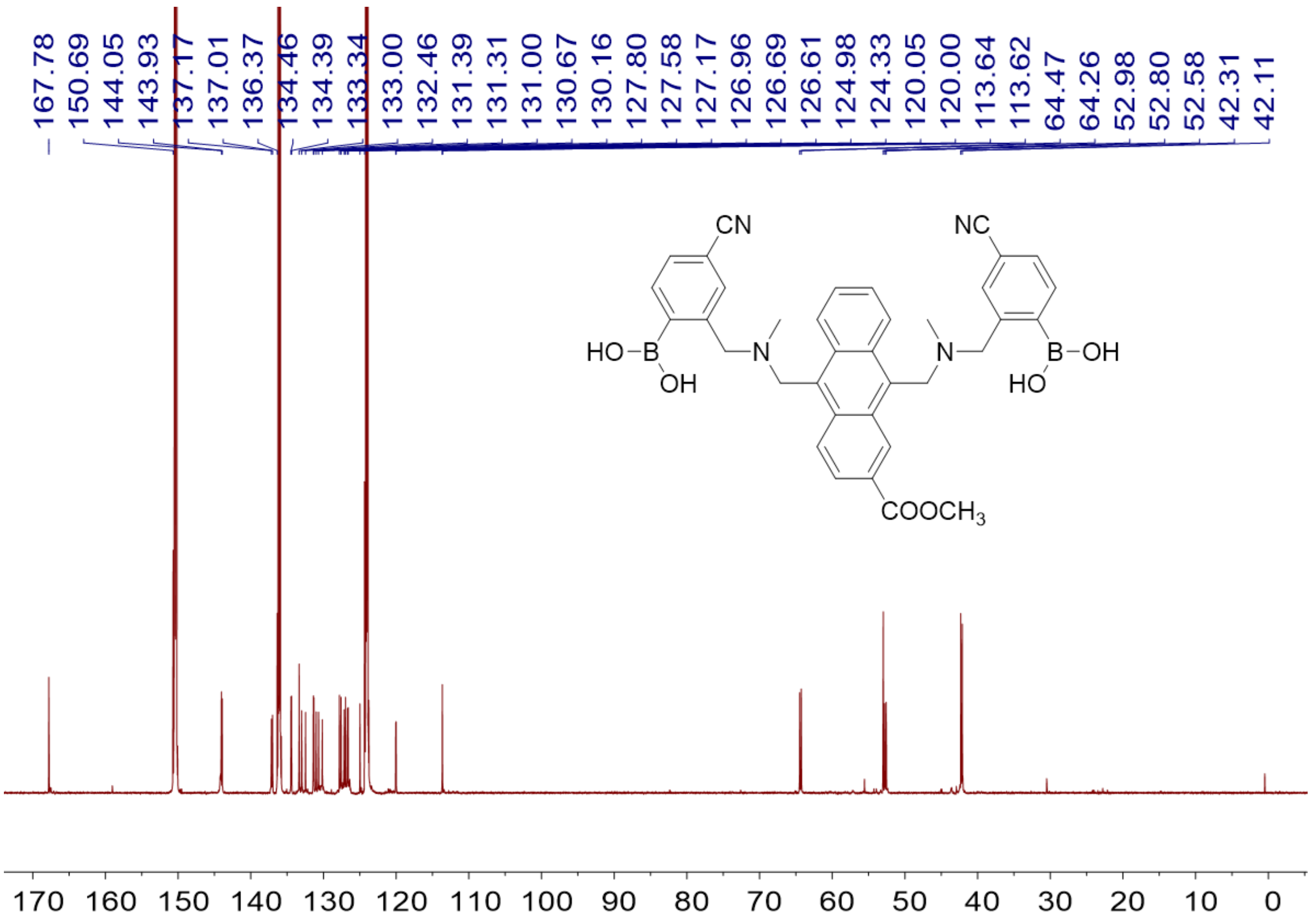

Supplementary Figure 20. ^{13}C NMR spectrum of compound **Mc-CDBA**.

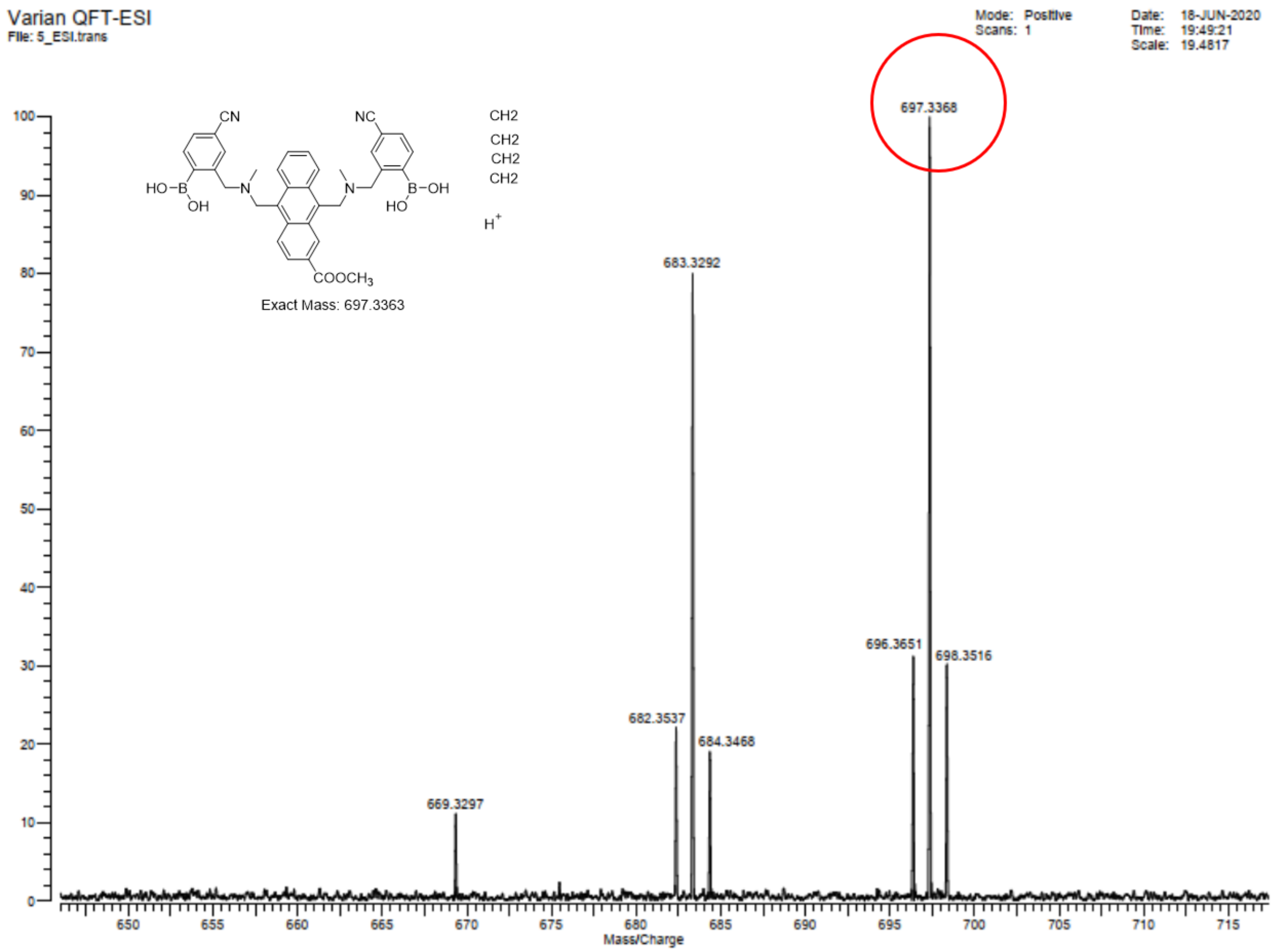

Supplementary Figure 21. High resolution mass spectrum (HR-MS) of compound **Mc-CDBA**.

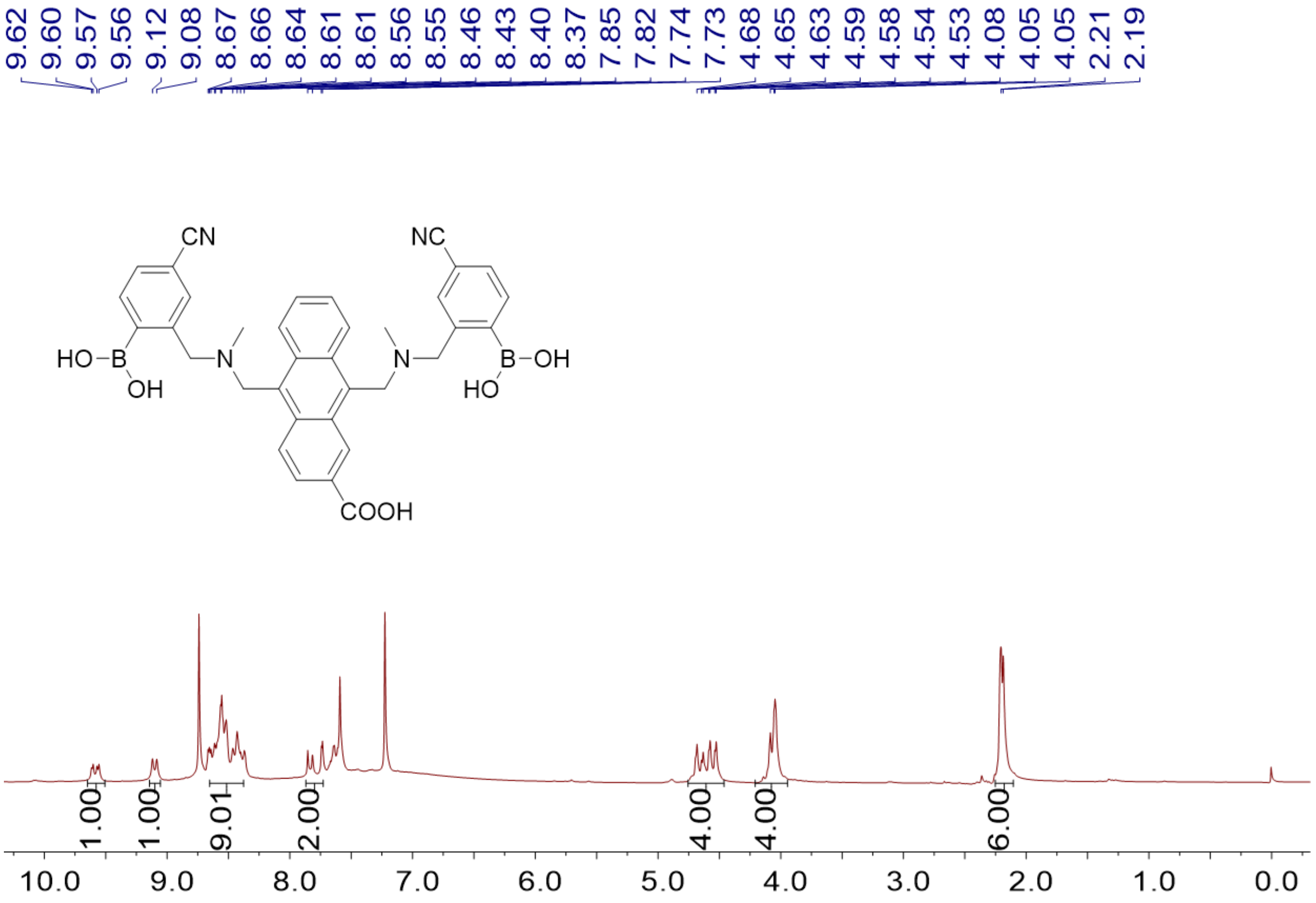

Supplementary Figure 22. ^{1}H NMR spectrum of compound **Ca-CDBA**.

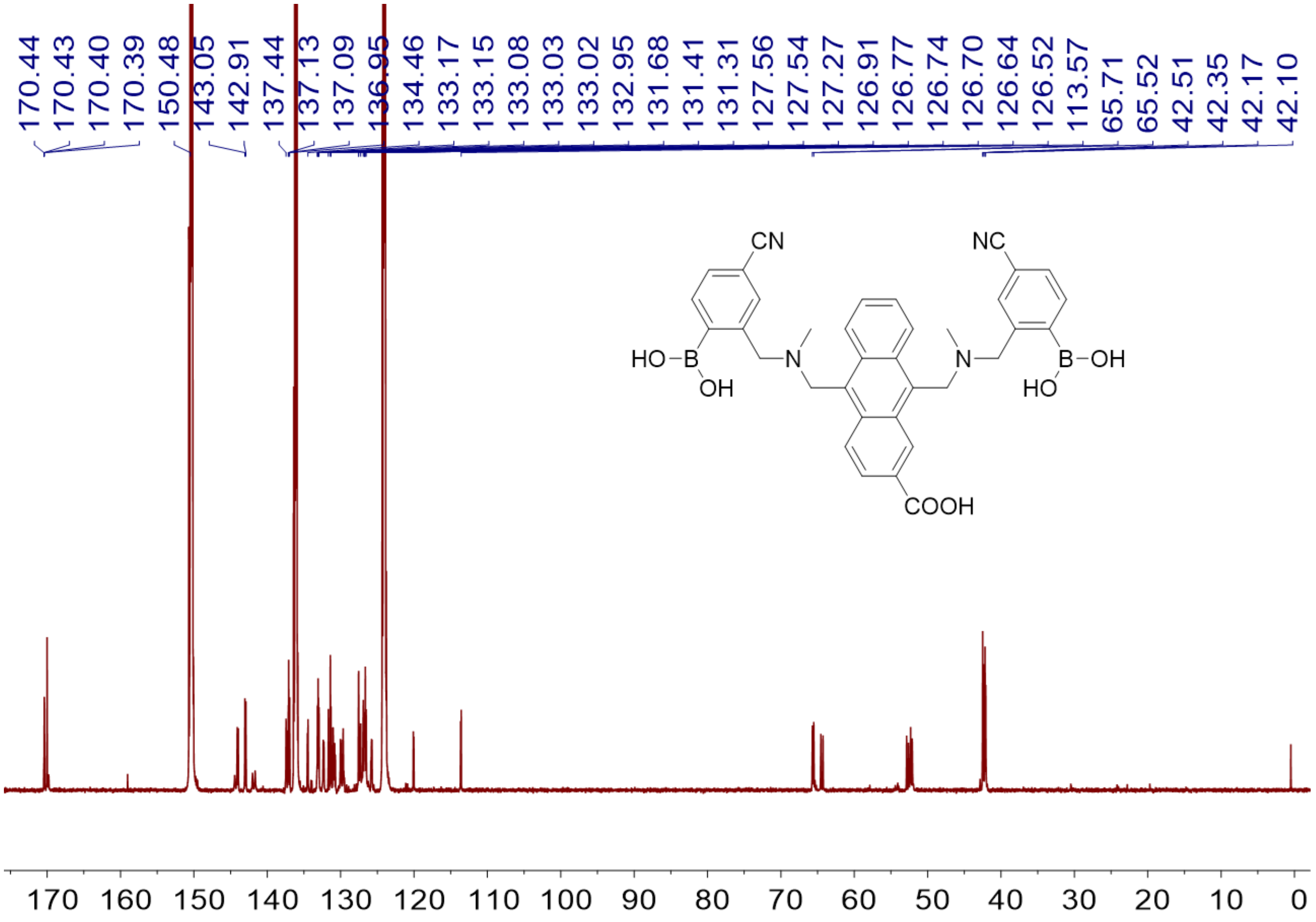

Supplementary Figure 23. ^{13}C NMR spectrum of compound **Ca-CDBA**.

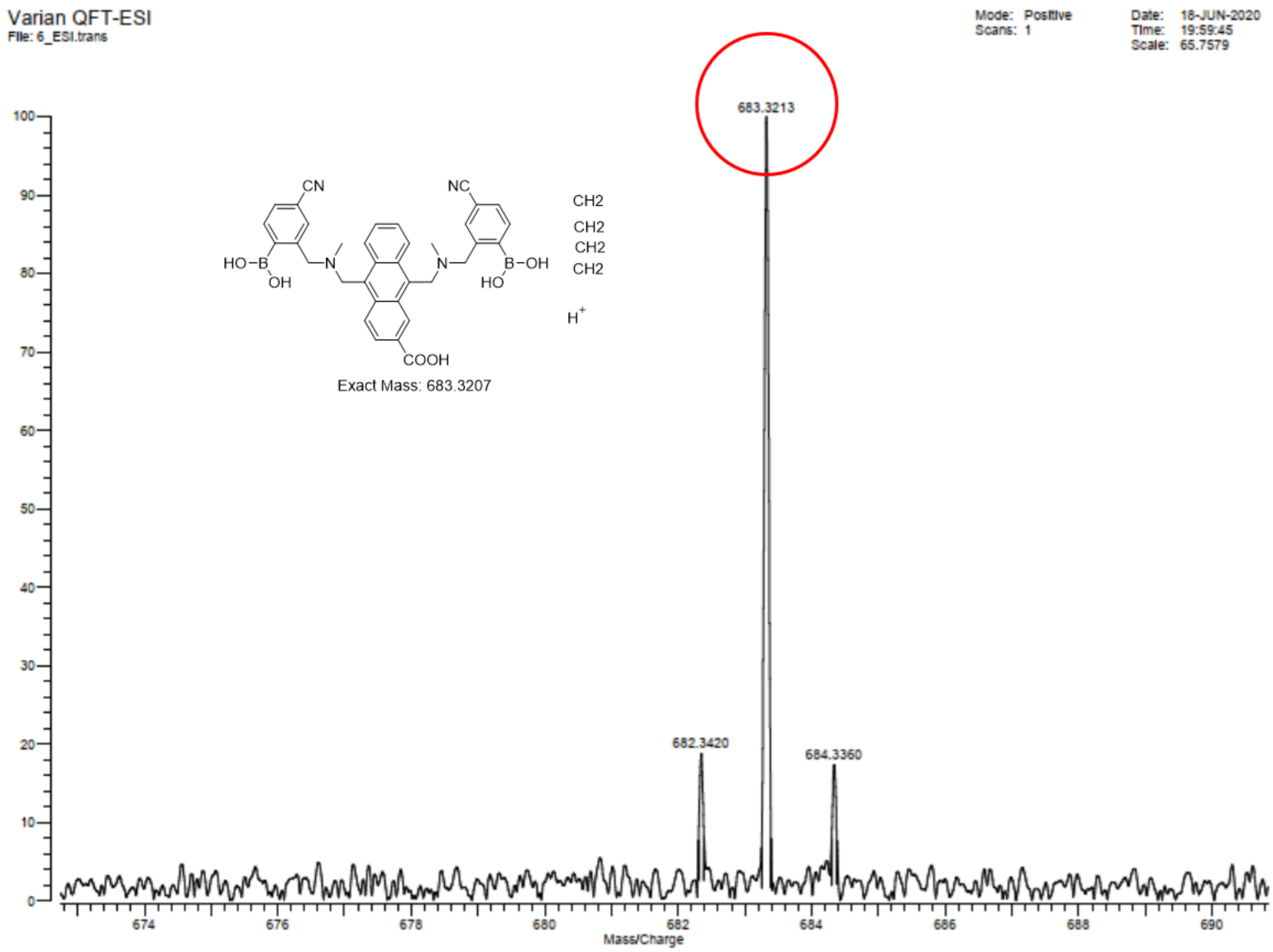

Supplementary Figure 24. High resolution mass spectrum (HR-MS) of compound **Ca-CDBA**.

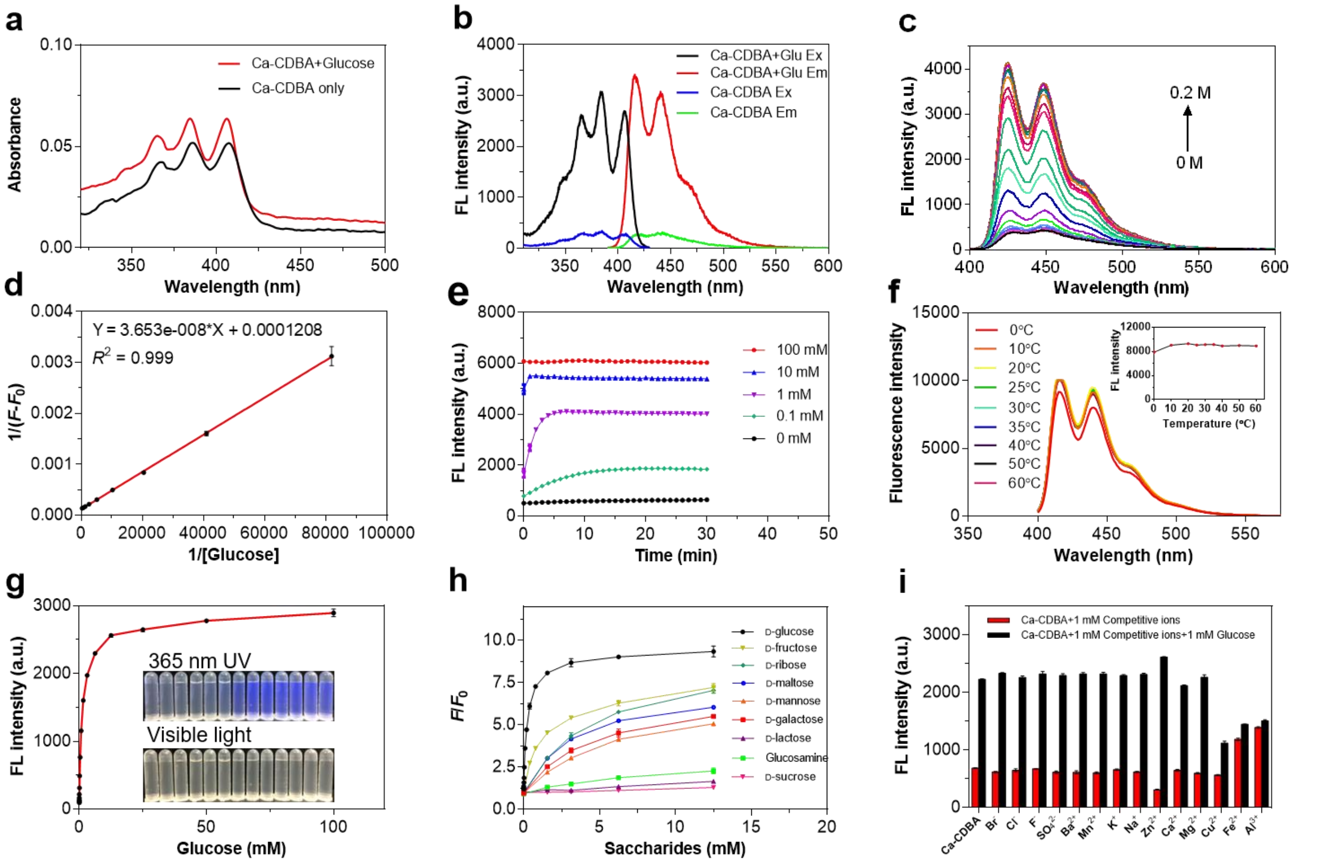

Supplementary Figure 25. Photophysical properties and selectivity of **Ca-CDBA**. (a) UV-visible absorption spectrum of 10 μM **Ca-CDBA** with or without 0.1 M glucose. (b) Fluorescence excitation and emission spectra of 10 μM **Ca-CDBA** before and after addition of 0.1 M glucose. (c) Fluorescence spectra of 10 μM **Ca-CDBA** towards increasing concentration of glucose (0−0.2 M). (d) B-H plot of **Ca-CDBA** in the glucose (12.2 μM−12.5mM) sensing. (e) Time-dependent fluorescence spectra of **Ca-CDBA** (10 μM) in presence of glucose (0.1−100 mM) for varying time intervals (0−30 min) at 25 °C. (f) Fluorescence spectra changes of 10 μM **Ca-CDBA** versus the solution temperature. Insert: Plot of temperature-dependent fluorescence intensity of 10 μM **Ca-CDBA** with 0.1 M glucose. (g) Fluorescence intensity increase of 10 μM **Ca-CDBA** upon addition of increased concentrations (0−0.1 M) of glucose. Insert: Photographs of **Ca-CDBA** with various concentrations of glucose under 365 nm UV and visible light, respectively. (h) Fluorescence response (F/F_0) of 10 μM **Ca-CDBA** in response to various saccharides including: glucose, fructose, ribose, maltose, mannose, galactose, lactose, glucosamine, sucrose. (i) Comparison of fluorescence intensity of 10 μM **Ca-CDBA** towards various species (1 mM) without or in the presence of glucose (1 mM): blank; Br^-; Cl^-; F^-; SO_4^{2-}; Ba^{2+}; Mn^{2+}; K^+; Na^+; Zn^{2+}; Ca^{2+}; Mg^{2+}; Cu^{2+}; Fe^{2+}; Al^{3+}. All tests were performed in 0.5% DMSO/PBS buffer, pH = 7.4 at 25 °C with λ_{ex} = 382 nm, λ_{em}

= 438 nm. Data are presented as the means ± SD (n = 3).

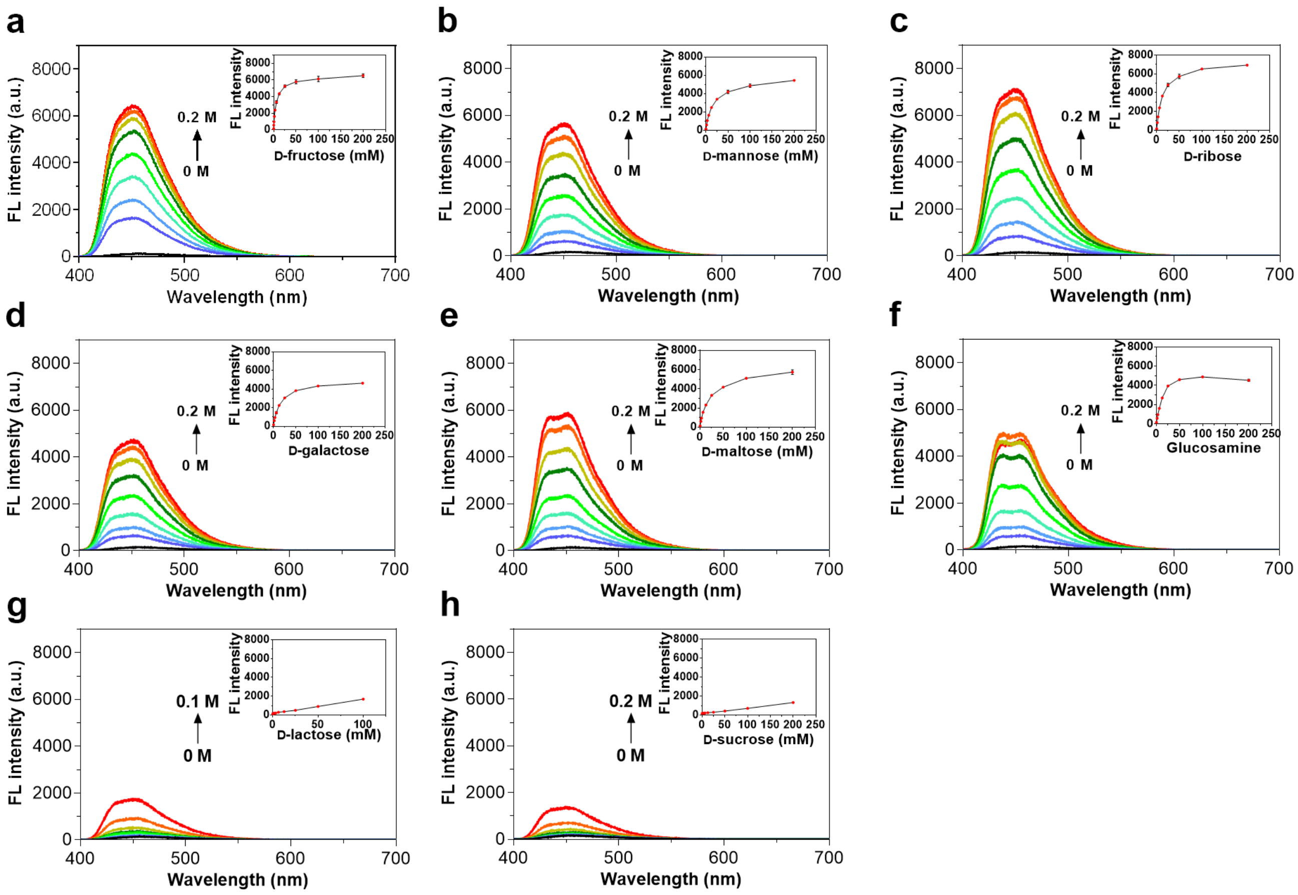

Supplementary Figure 26. Fluorescence response of **Mc-CDBA** with various saccharides (0−0.2 M): D-fructose (a), D-maltose (b), D-ribose (c), D-galactose (d), D-mannose (e), Glucosamine (f), D-lactose (g, 0−0.1M) and D-sucrose (h). 10 μM of **Mc-CDBA** in 0.5% MeOH/PBS (pH 7.4) at 25 °C, λ_{ex} = 393 nm.

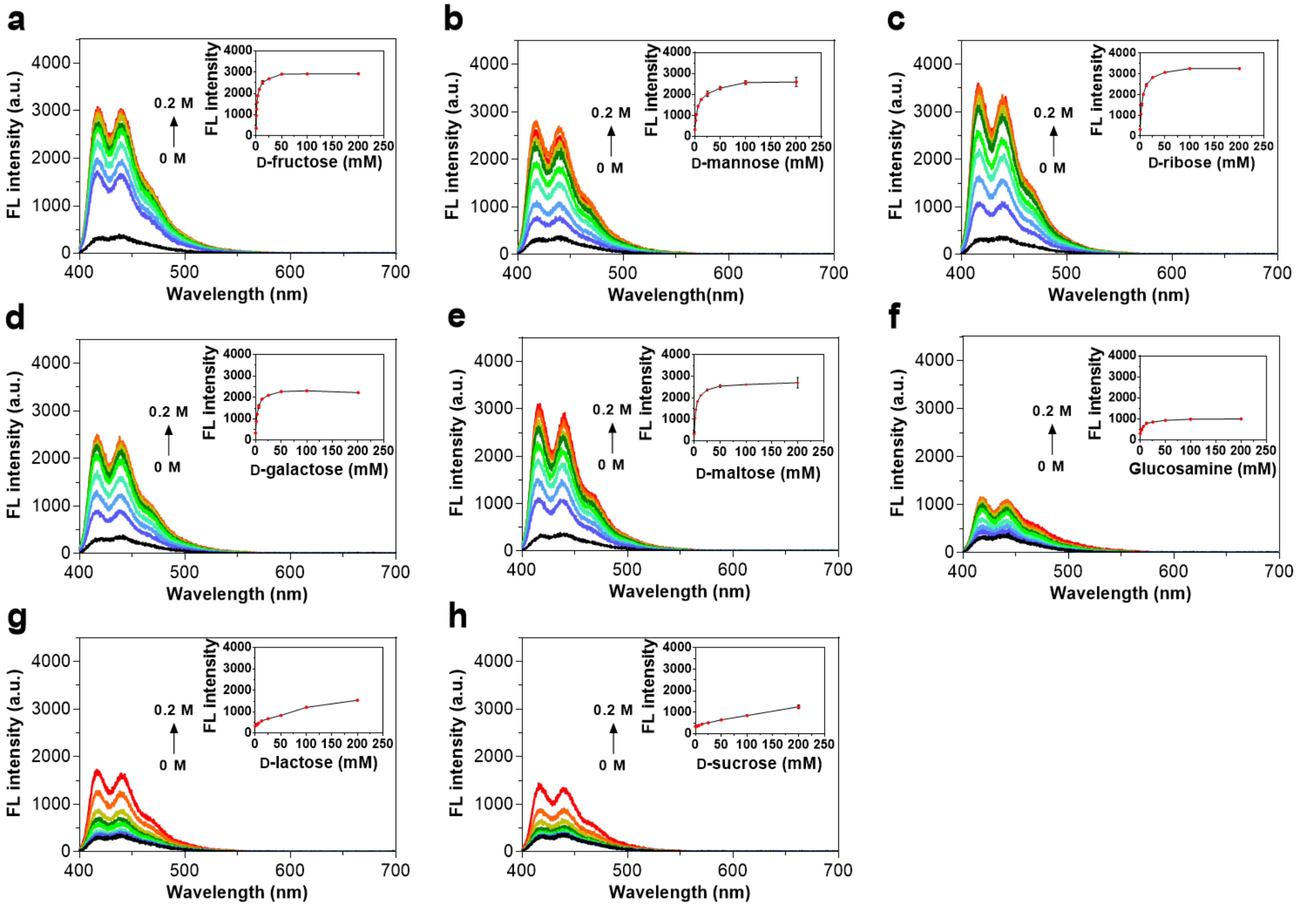

Supplementary Figure 27. Fluorescence response of **Ca-CDBA** with various saccharides (0−0.2 M): D-fructose (a), D-maltose (b), D-ribose (c), D-galactose (d), D-mannose (e), Glucosamine (f), D-lactose (g) and D-sucrose (h). 10 μM of **Ca-CDBA** in 0.5% DMSO/PBS (pH 7.4) at 25 °C, λ_{ex} = 382 nm.

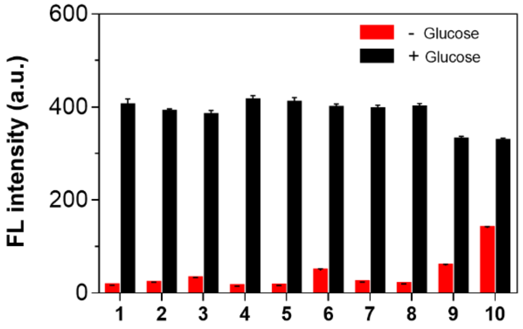

Supplementary Figure 28. Comparison of fluorescence intensity of 10 μM **Mc-CDBA** towards various glucose metabolic species (1 mM) with or without glucose (1 mM): (1) Blank; (2) Malic acid; (3) Oxaloacetic acid; (4) *a*-Ketoglutaric acid; (5) L-(+)-Lactic acid; (6) D-Glucose-6-phosphate; (7) D-Fructose-1,6-bisphosphate; (8) D-Fructose-1,6-bisphosphate; (9) Uridine diphosphate glucose; (10) D-Ribulose 5-phosphate. All tests were performed in 0.5% MeOH/PBS buffer, pH = 7.4 at 25 °C with λ_{ex} = 393 nm, λ_{em} = 457 nm. Data are presented as the means ± SD (n = 3).

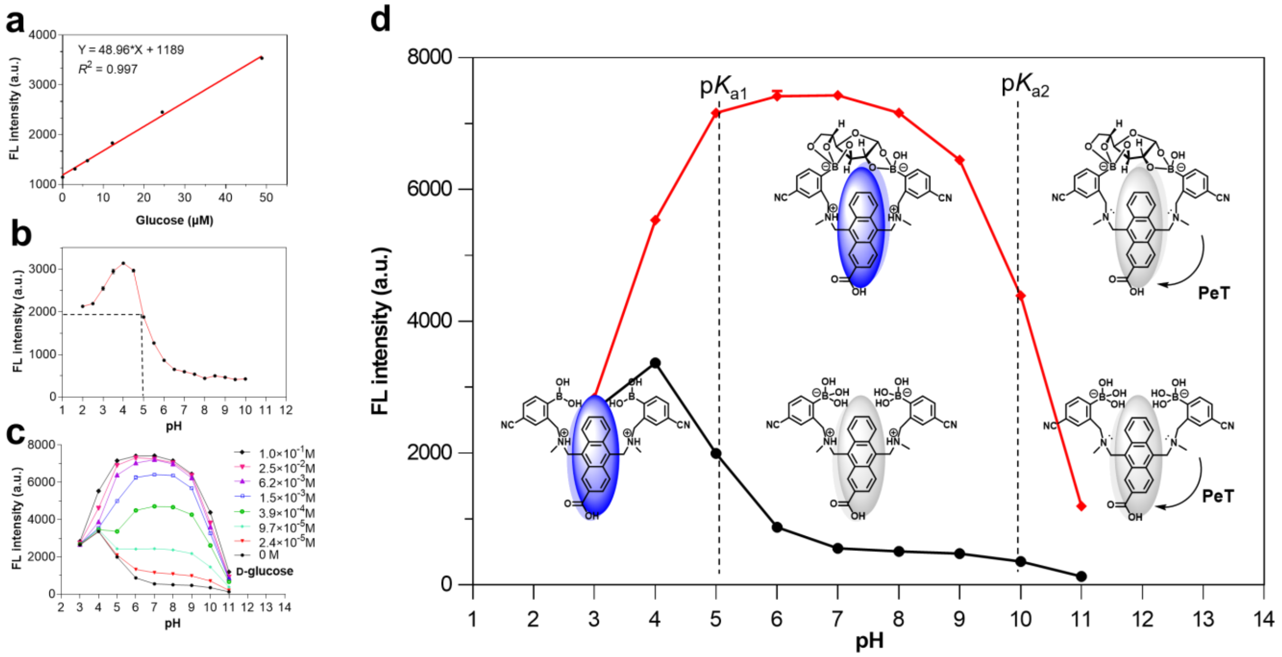

Supplementary Figure 29. Sensitivity and glucose sensing mechanism of **Ca-CDBA**. (a) Linear changes in fluorescence intensity of 10 μM **Ca-CDBA** with various concentrations of glucose (0−48 μM). (b) pH-dependent (pH from 2 to 10) fluorescence emission intensity of 10 μM **Ca-CDBA** in PBS buffer. (c) pH-dependent (pH from 3 to 11) fluorescence emission intensity of 10 μM **Ca-CDBA** with various glucose (0−0.1 M). (d) Proposed fluorescence sensing mechanism of **Ca-CDBA** for turn-on detection of glucose. All tests were performed in 0.5% DMSO/PBS buffer, at 25 °C with λ_{ex} = 382 nm, λ_{em} = 438 nm. Data are presented as the means ± SD (n = 3).

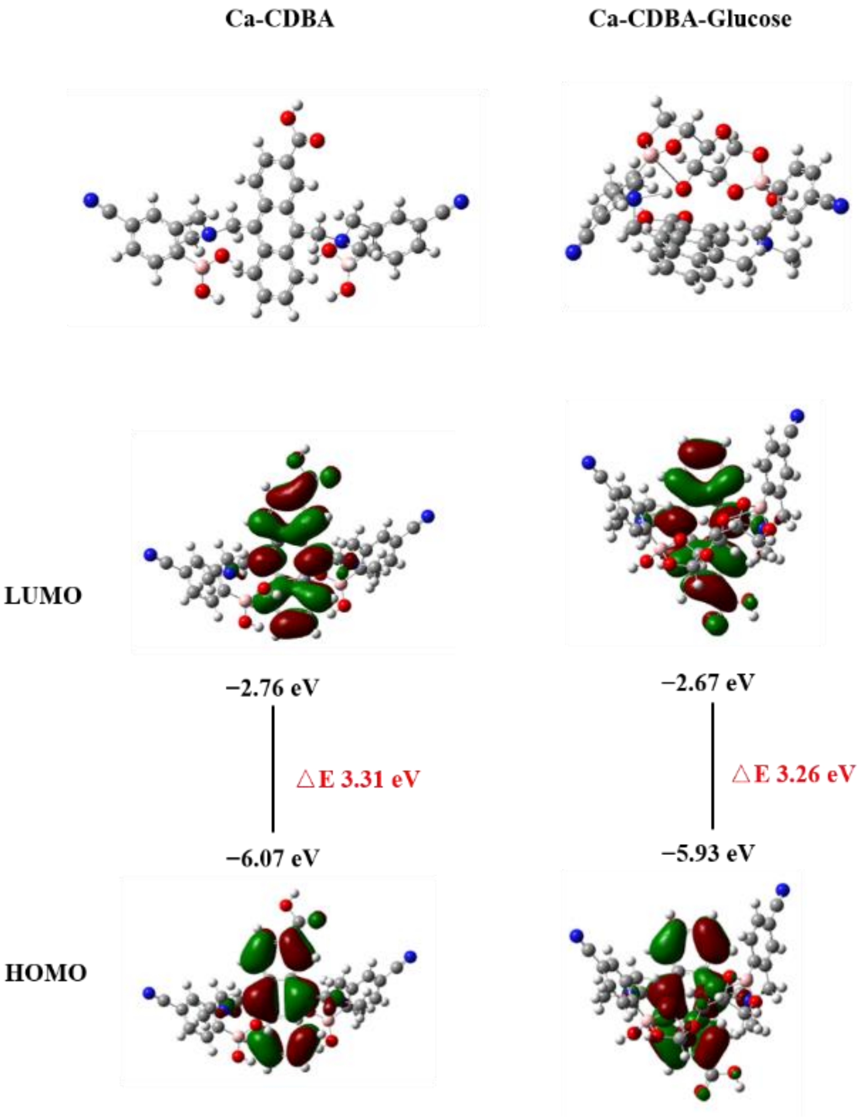

Supplementary Figure 30. Structure optimization diagram and theoretical calculation of probe **Ca-CDBA** and its glucose borate compound. Atom color: gray represents carbon atoms; white represents hydrogen atoms; blue represents nitrogen atoms; pink represents boron atoms; red represents oxygen atoms.

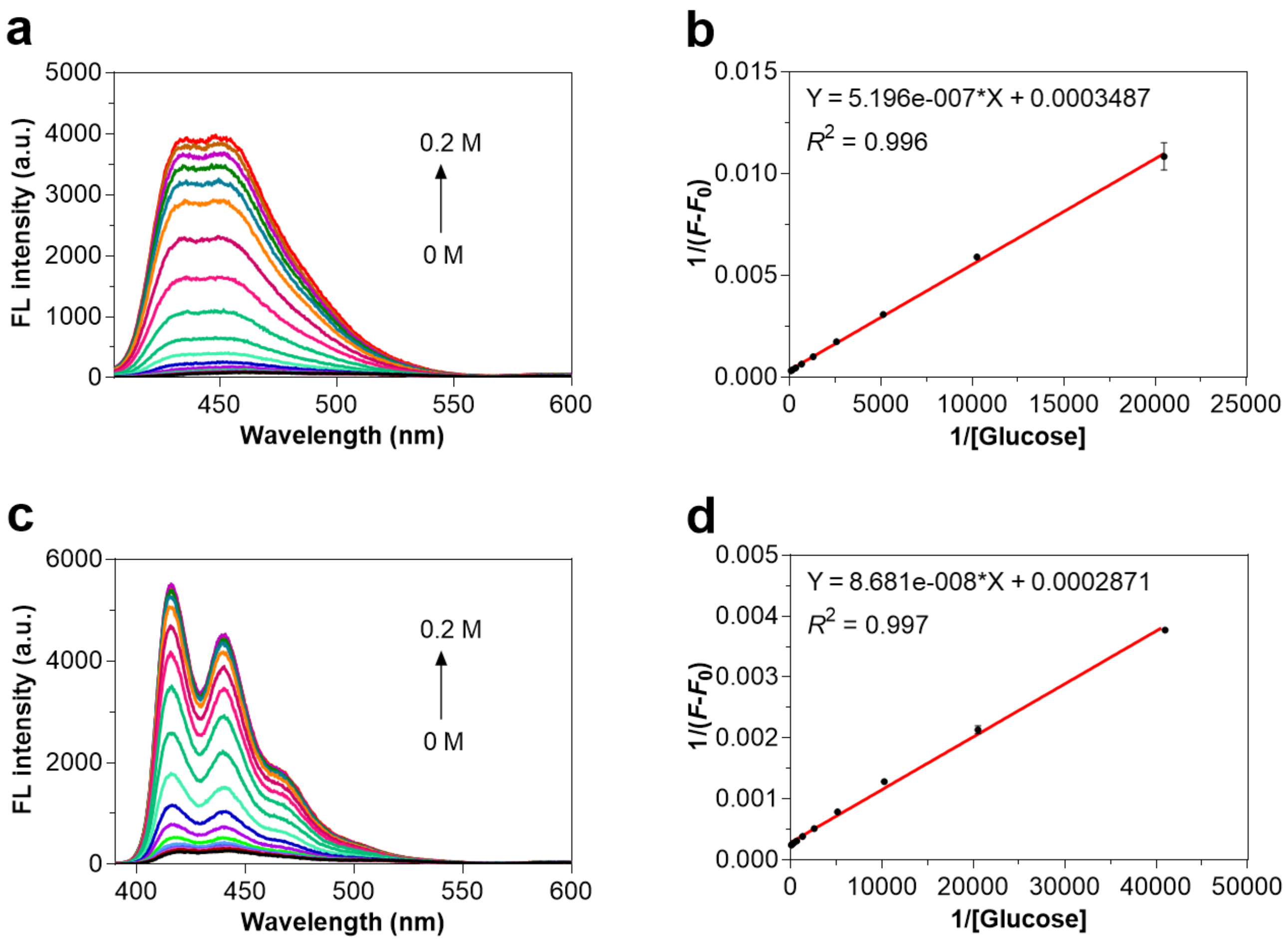

Supplementary Figure 31. Glucose detection of **Mc-CDBA** and **Ca-CDBA** in DMEM Medium. (a) Fluorescence changes of 10 μM **Mc-CDBA** in 0.5% MeOH/DMEM with various concentrations of glucose (0−0.2 M). (b) B-H plot of the **Mc-CDBA** fluorescence changes versus glucose concentrations (48.8 μM−12.5 mM). (c) Fluorescence changes of 10 μM **Ca-CDBA** in 0.5% DMSO/DMEM with various concentrations of glucose (0−0.2 M). (d) B-H plot of the **Ca-CDBA** fluorescence changes versus glucose concentrations (24.4 μM−12.5 mM). Data are presented as the means ± SD (n = 3).

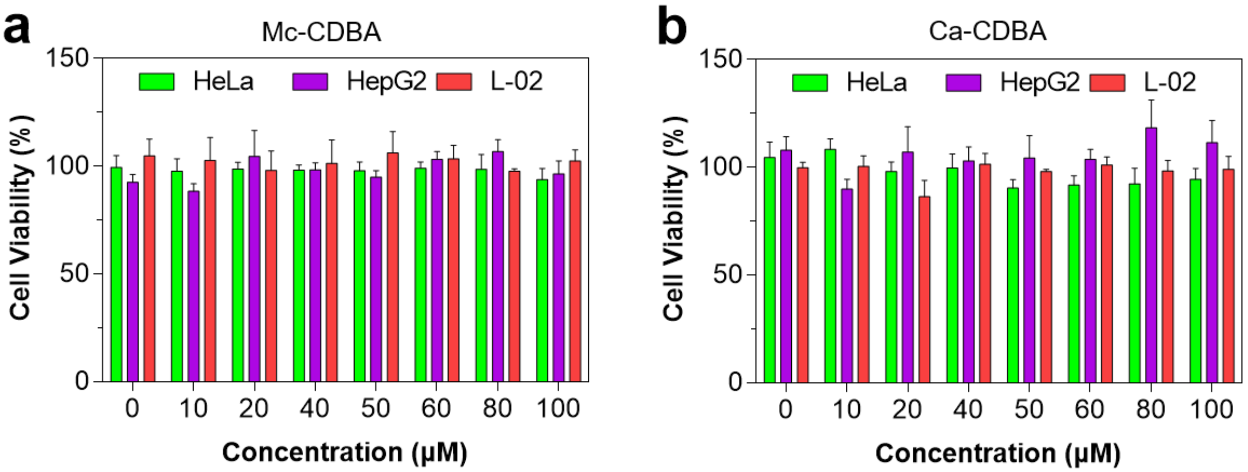

Supplementary Figure 32. Cell viability assay. (a-b) Cell viabilities of **Mc-CDBA** (a) and **Ca-CDBA** (b) at various concentrations (0−100 μM) for HeLa, HepG2 and L-02 cells after 24 h incubation.

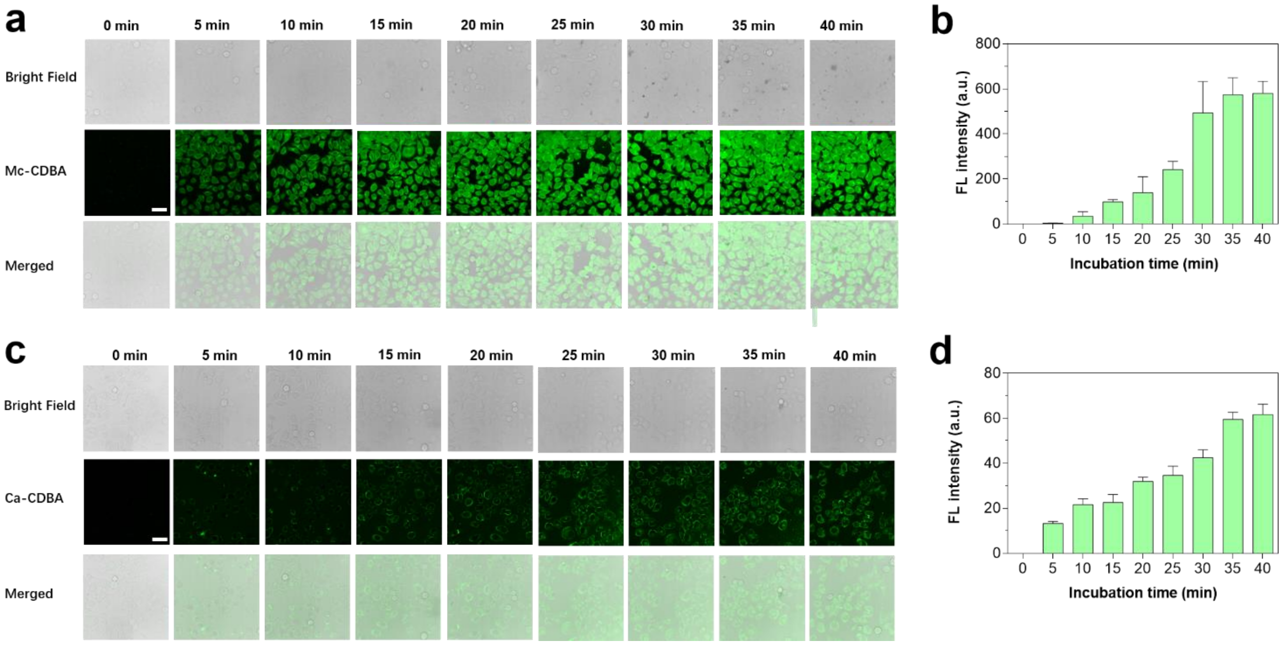

Supplementary Figure 33. Cell uptake assay of **Mc-CDBA** and **Ca-CDBA** in HeLa cells. (a-b) Confocal microscopy images (a) and fluorescence intensity (b) of HeLa cells incubated with 50 μM **Mc-CDBA** for 0, 5, 10, 15, 20, 25, 30, 35, 40 min, respectively. (c-d) Confocal microscopy images (c) and fluorescence intensity (d) of HeLa cells incubated with 50 μM **Ca-CDBA** for 0, 5, 10, 15, 20, 25, 30, 35, 40 min, respectively. Cell images were captured on a Leica SP8 with $\lambda_{ex/em}$ = 405/410−600 nm (scale bar = 50 μm).

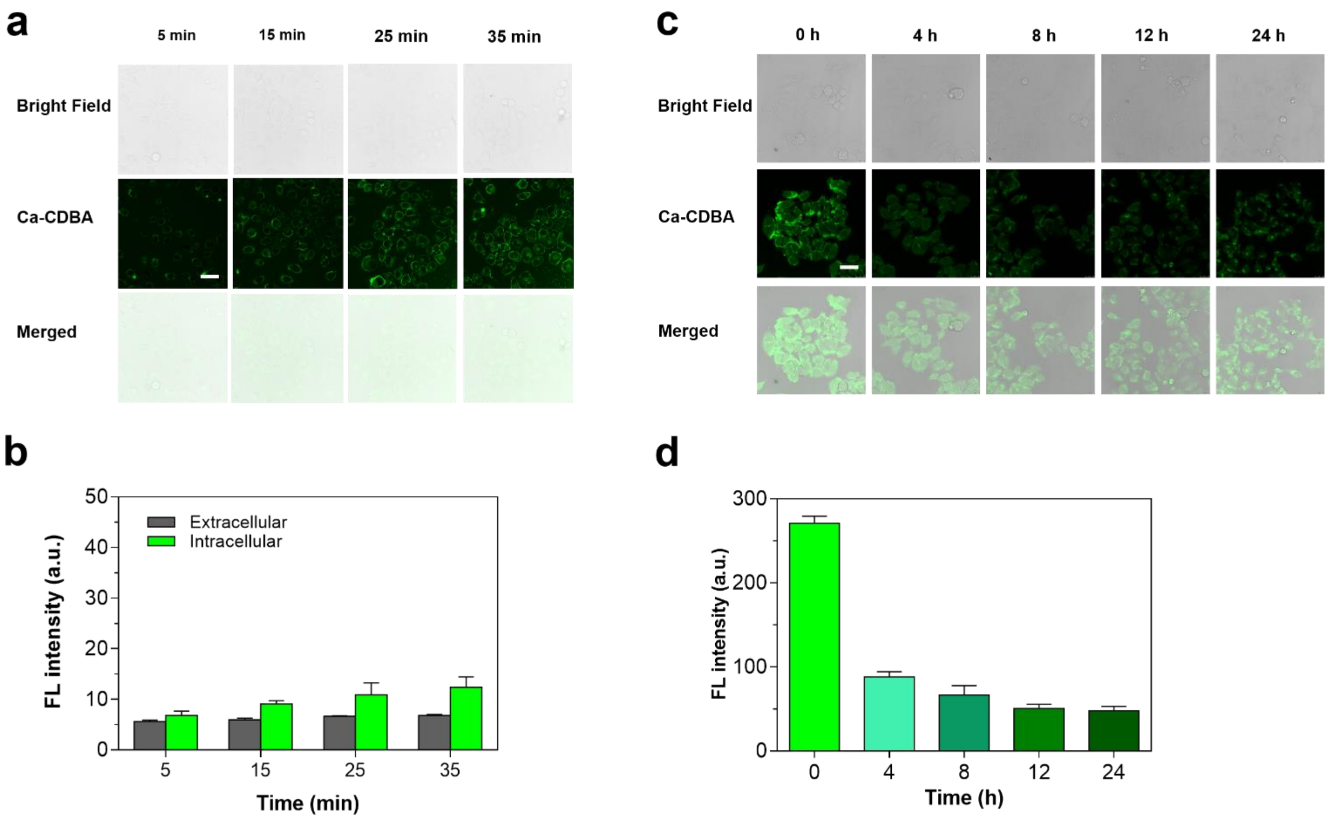

Supplementary Figure 34. Confocal microscopy imaging of **Ca-CDBA**. (a-b) Confocal microscopy images (a) and the fluorescence intensity comparison between extracellular and intracellular (b) of HeLa cells incubated with 50 μM **Ca-CDBA** for 5, 15, 25, 35 min, respectively. (c-d) Confocal microscopy images (c) and fluorescence intensity (d) of HeLa cells preincubated with glucose-free DMEM for 0, 4, 8, 12, 24 h, then incubated with 50 μM **Ca-CDBA** for 30 min. Cell images were captured on a Leica SP8 with $\lambda_{ex/em}$ = 405/410−600 nm (scale bar = 50 μm).

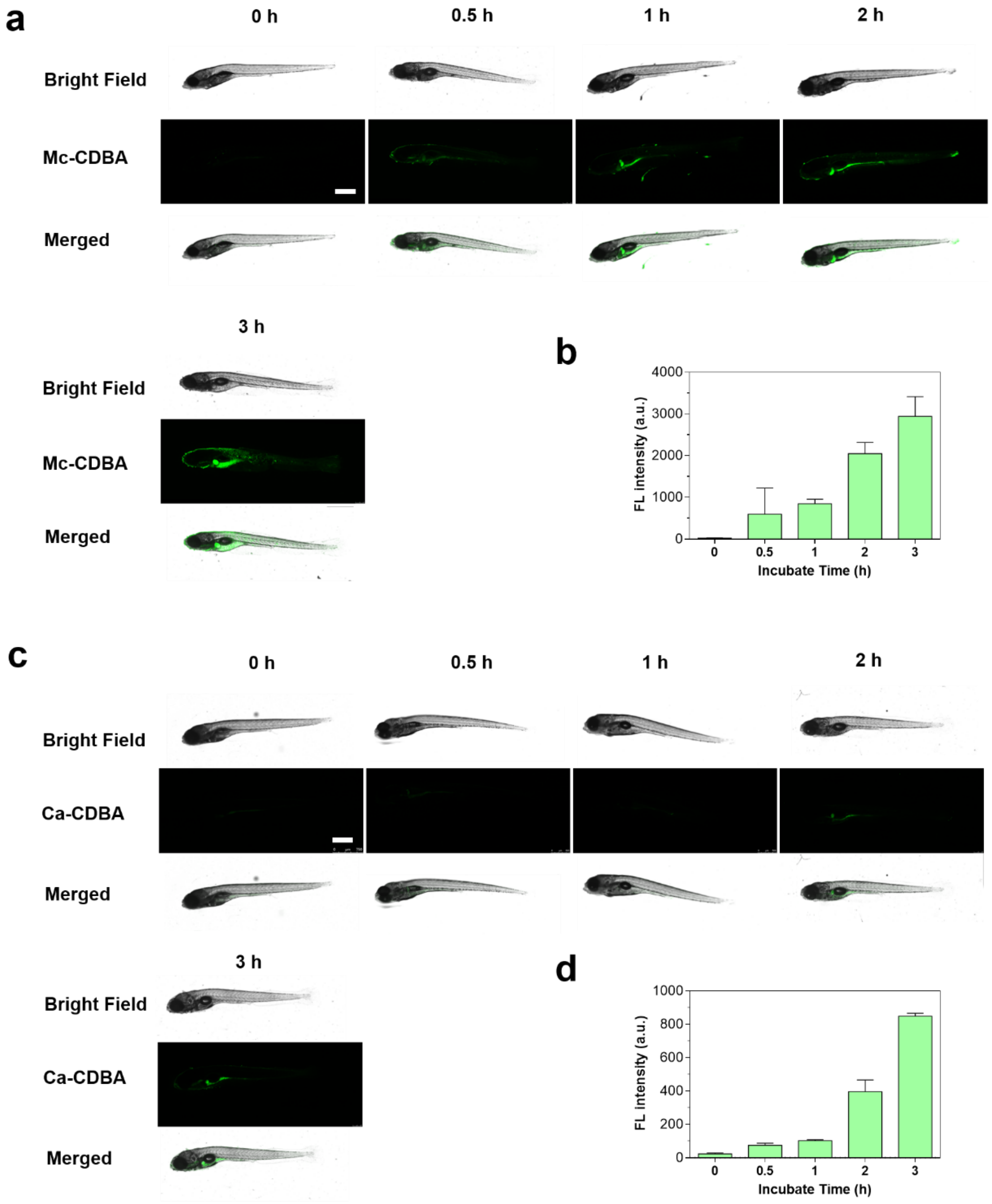

Supplementary Figure 35. Zebrafish uptake tests of **Mc-CDBA** and **Ca-CDBA**. (a-b) Confocal microscopy images (a) and the fluorescence intensity (b) of zebrafish embryos incubated with 50 μM **Mc-CDBA** for 0, 0.5, 1, 2, 3 h, respectively. (c-d) Confocal microscopy images (c) and the fluorescence intensity (d) of zebrafish embryos incubated with 50 μM **Ca-CDBA** for 0, 0.5, 1, 2, 3 h, respectively. Zebrafish images were captured on a Leica TCS SP8 with $\lambda_{ex/em}$ = 405/410−600 nm. The scale bar represents 500 μm.

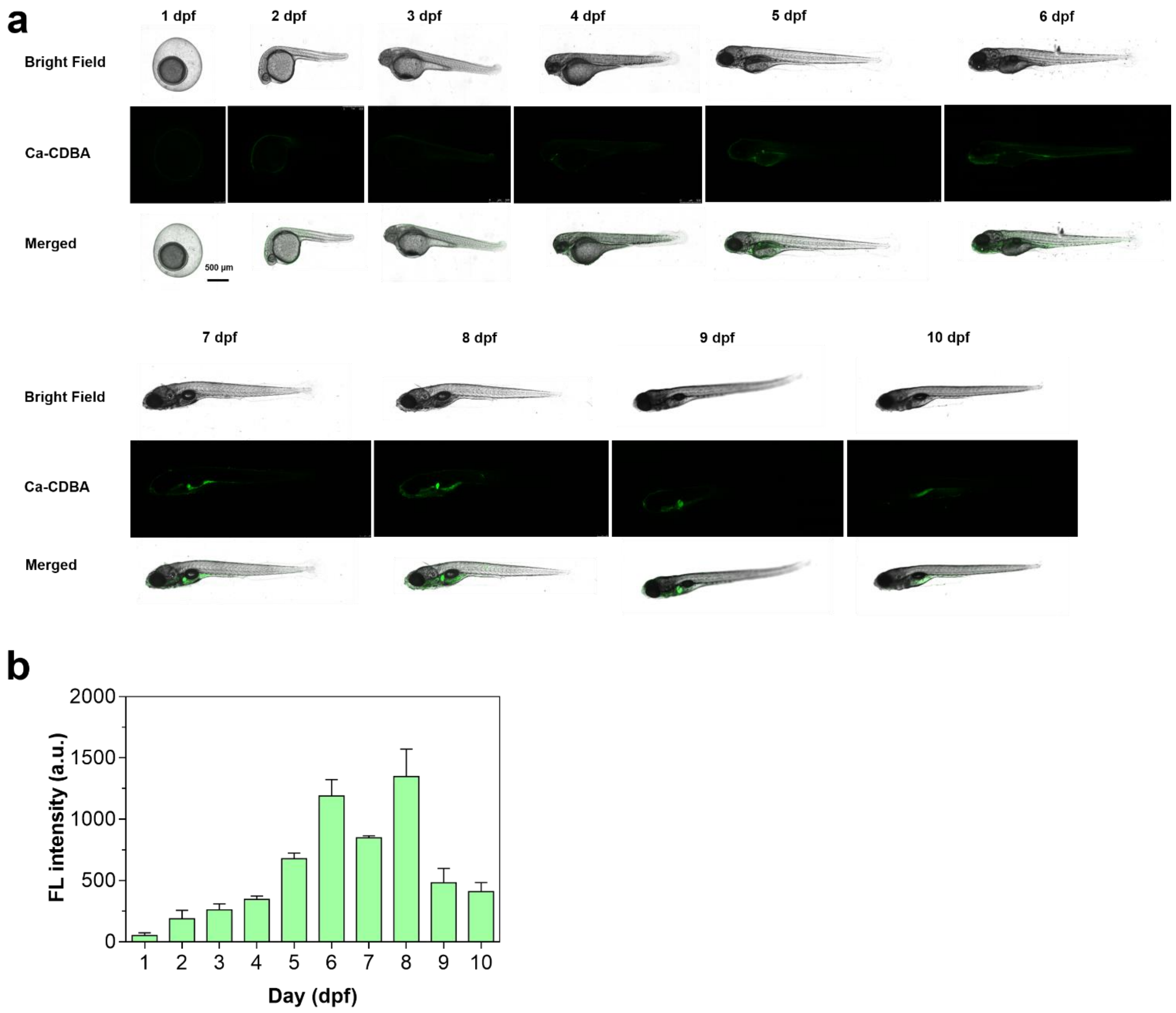

Supplementary Figure 36. Fluorescence imagining of **Ca-CDBA** for 1−10 dpf zebrafish. (a-b) Confocal microscopy images (a) and the fluorescence intensity (b) of 1−10 dpf zebrafish embryos incubated with 50 μM **Ca-CDBA** for 3 h, respectively. Zebrafish images were captured on a Leica TCS SP8 with $\lambda_{ex/em}$ = 405/410−600 nm.

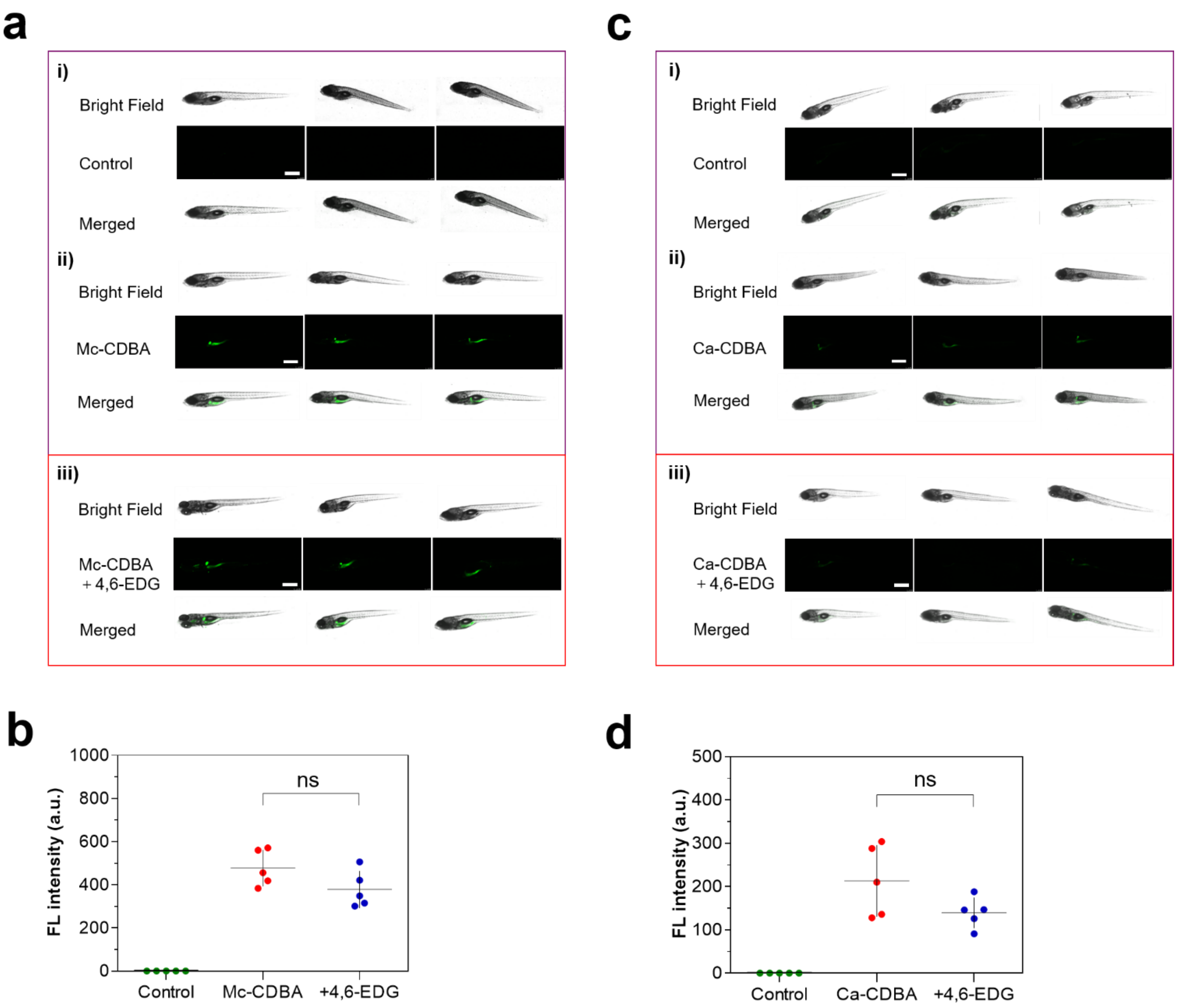

Supplementary Figure 37. Efficacy evaluation tests of 4,6-EDG in zebrafish embryos. (a-b) Fluorescence confocal images (a) and fluorescence intensity (b) of 7 dpf zebrafish embryos preincubated with blank medium (i), blank medium (ii), and 20 mM 4,6-EDG (iii) for 4 h, then the group ii and iii further incubated with 50 μM **Mc-CDBA** for 1 h. (c-d) Fluorescence confocal images (c) and fluorescence intensity (d) of 7 dpf zebrafish embryos preincubated with blank medium (i), blank medium (ii), and 20 mM 4,6-EDG (iii) for 4 h, then the group ii and iii further incubated with 50 μM **Ca-CDBA** for 1 h. Zebrafish images were captured on a Leica TCS SP8 with $\lambda_{ex/em}$ = 405/410−600 nm (n = 5, with levels of significance set at n.s. no significant difference). The scale bar represents 500 μm.

Supplementary table 1. Characteristics of the diboronic acid-based probes for glucose

Probe	K_a (M^{-1}) [a] Glucose	$\lambda_{ex}/\lambda_{em}$ (nm)	pK_a	LOD (μM)	Φ_{min} [b]	Φ_{max} [c]	F/F_0 [d]
**Mc-CDBA** [e]	7.1×10^2 ± 11	393/457	4.2	1.37	0.018	0.529	47.8-fold
**Ca-CDBA** [f]	4.5×10^3 ± 31	382/438	--[g]	2.59	0.097	0.671	9.8-fold

[a] Binding affinity for glucose. [b] Fluorescence quantum yield (Φ_{min}) was determined without glucose. [c] Fluorescence quantum yield (Φ_{max}) was determined with 0.1 M glucose. [d] Fluorescence changes for 0.1 M glucose. [e] Data were measured in 0.5% MeOH/PBS buffer. [f] Data were measured in 0.5% DMSO/PBS buffer. -- [g]: The fluorescence quenching of **Ca-CDBA** in pH 2−4 (Figure S29b) makes it impossible to acquire the accurate pK_a value.

Supplementary table 2. Binding affinity (K_a) of the probes for different saccharides

K_a [a] (M^{-1}) Saccharide	**Mc-CDBA** [b]	**Ca-CDBA** [c]
K_a (M^{-1}) D-glucose	7.1×10^2 ± 11	4.5×10^3 ± 31
K_a (M^{-1}) D-fructose	1.8×10^2 ± 6.7	8.5×10^2 ± 16
K_a (M^{-1}) D-ribose	77 ± 2.5	2.7×10^2 ± 10
K_a (M^{-1}) D-galactose	68 ± 4.3	3.6×10^2 ± 24
K_a (M^{-1}) D-maltose	48 ± 2.3	3.7×10^2 ± 27
K_a (M^{-1}) D-mannose	62 ± 0.71	2.1×10^2 ± 12
K_a (M^{-1}) Glucosamine	88 ± 2.4	3.9×10^2 ± 21
K_a (M^{-1}) D-lactose	5.2 ± 0.78	49 ± 4.5
K_a (M^{-1}) D-sucrose	3.6 ± 0.35	57 ± 6.8

[a] Date were determined by non-linear mathematical method based on triplicate measurements (R^2 > 0.99) (Figure S26-S27). [b] Data were measured in 0.5 % MeOH/PBS (pH 7.4). [c] Data were measured in 0.5% DMSO/PBS (pH 7.4).

Supplementary table 3. The results of glucose detection in sheep plasma by commercial glucose analysis kit and the probe **Mc-CDBA** (B-H Plot, n = 3)

Precision	Glucose added (mM)	Commercial analysis kit			**Mc-CDBA**		
		Glucose found (mM) [a]	Recovery (%)	RSD (%)	Glucose found (mM)	Recovery (%)	RSD (%)
Intra-assay	0.00	14.06±0.50	NA [b]	3.55	13.74±0.10	NA	0.74
	6.25	20.97±0.53	110.55	2.52	19.97±0.11	100.33	0.56
	12.50	26.89±0.96	102.65	3.57	25.89±0.25	96.02	0.97
	25.00	40.91±0.90	107.43	2.21	38.93±0.12	101.02	0.32
	50.00	70.01±1.21	111.90	1.73	65.80±0.39	103.70	0.59
Inter-assay	0.00	13.64±0.39	NA	2.88	13.66±0.20	NA	1.43
	6.25	20.34±0.78	107.14	3.83	19.87±0.06	99.25	0.28
	12.50	26.26±0.59	100.99	2.25	26.26±0.45	100.74	1.72
	25.00	40.11±1.84	105.88	4.59	38.75±0.08	100.36	0.22
	50.00	66.50±2.40	105.72	3.61	68.09±1.86	108.85	2.73

[a] The value was obtained from the calculated glucose level by B-H Plot×Dilution Ratio (500). NA [b] means not available.

Supplementary table 4. The inter-day variations of plasma glucose detection by **Mc-CDBA** (B-H Plot, n = 3)

Glucose added (mM)	Glucose found (mM) [a]	Recovery (%)	RSD (%)
0.00	12.95±0.45	NA [b]	3.51
6.25	18.68±0.54	91.63	2.87
12.50	24.37±1.21	91.37	4.98
25.00	37.21±0.61	97.05	1.63
50.00	64.41±3.31	102.91	5.15

[a] The value was obtained from the calculated glucose level by B-H Plot×Dilution Ratio (500). NA [b] means not available.

Supplementary table 5. The results of glucose detection in sheep plasma by commercial glucose analysis kit and the probe **Ca-CDBA** (B-H Plot, n = 3)

Precision	Glucose added (mM)	Commercial analysis kit			**Ca-CDBA**		
		Glucose found (mM)[a]	Recovery (%)	RSD (%)	Glucose found (mM)	Recovery (%)	RSD (%)
Intra-assay	0.00	14.09±0.36	NA[b]	2.59	13.01±0.27	NA	2.08
	6.25	20.85±0.39	108.12	1.88	19.39±0.13	102.14	0.65
	12.50	28.09±0.35	111.99	1.25	25.67±0.31	101.28	1.20
	25.00	42.90±0.75	115.23	1.74	37.60±0.10	98.36	0.26
	50.00	66.11±3.40	104.03	5.14	64.15±0.39	102.28	0.61
Inter-assay	0.00	13.43±0.26	NA	1.96	13.32±0.11	NA	0.80
	6.25	20.95±0.61	120.31	2.92	19.57±0.24	99.98	1.25
	12.50	27.38±0.45	111.62	1.66	25.71±0.36	99.17	1.38
	25.00	41.13±0.96	110.79	2.34	38.00±0.27	98.72	0.72
	50.00	65.98±3.07	105.10	4.65	63.50±1.06	100.36	1.67

[a] The value was obtained from the calculated glucose level by B-H Plot×Dilution Ratio (500). NA [b] means not available.

Supplementary table 6. The inter-day variations of plasma glucose detection by **Ca-CDBA** (B-H Plot, n = 3)

Glucose added (mM)	Glucose found (mM) [a]	Recovery (%)	RSD (%)
0.00	13.50±0.31	NA [b]	2.30
6.25	19.82±0.48	101.17	2.41
12.50	25.98±0.98	99.80	3.78
25.00	37.73±0.54	96.93	1.43
50.00	62.11±2.00	97.21	3.23

[a] The value was obtained from the calculated glucose level by B-H Plot×Dilution Ratio (500). NA [b] means not available.

Supplementary table 7. Key information of the reported boronic acid-based glucose probes

Probe	Test sample (Solvent)	$K_{a\text{-}glucose}$ (M^{-1})	Linear Range (mM)/LOD (μM)	F/F_0 [a]	Author /Year
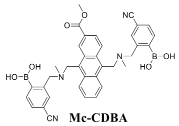 Mc-CDBA	Plasma, DMEM, Cell, Zebrafish	$7.1\times10^2\pm11$	0.0122–12.5/ **1.37**	**47.8** ↑ [b] [Glu] [c]= 0.1 M	This work
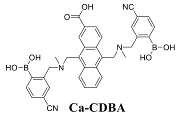 Ca-CDBA	Plasma, DMEM, Cell, Zebrafish	$4.5\times10^3\pm31$	0.0122–12.5/ **2.59**	**9.8** ↑ [Glu] = 0.1 M	This work
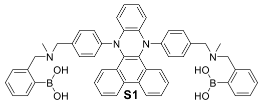 S1	80% MeOH/PBS	816	—/ 9.4	—	Javier, et al. **2021**[2]
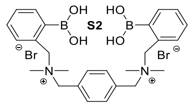 S2	PBS	1000	1–30/ —	—	Wang B, et al. **2019**[3]
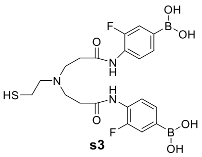 s3	4% MeOH/PBS	167	1–10/ —	—	Sharma B, et al. **2016**[4]
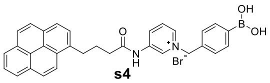 s4	2% MeOH/Carbonate	1378	—/ 10	—	Huang Y J, et al. **2013**[5]
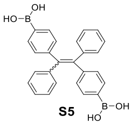 S5	Artificial urine	—	0.2–5/ 200	5.4 ↑ [Glu] = 0.005 M	Liu Y, et al. **2011**[6]
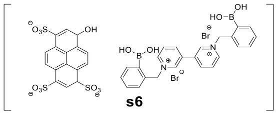 s6	PBS	1900	1–35/ —	1.8 ↑ [Glu] = 0.03 M	Schiller A, et al. **2007**[7]
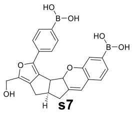 s7	30% MeOH/PBS	40000	—/ —	0.5 ↓[d] [Glu] = 0.1 M	Yang W, et al. **2001**[8]
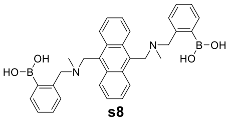 s8	33% MeOH/PBS	3981	0.3–1/ —	14.5 ↑ [Glu] = 0.1 M	James T D, et al. **1995**[9]

F/F_0 [a]: Maximum percentage of fluorescence increase; ↑ [b]: "Turn-on"; [Glu] [c] represents the concentration of glucose; ↓ [d]: "Turn-off".

References

1. Kawanishi, T.; Romey, M. A.; Zhu, P. C.; Holody, M. Z.; Shinkai, S. A study of boronic acid based fluorescent glucose sensors. *J. Fluoresc.* **2004**, *14*, 499–512.

2. Ramos-Soriano, J.; Benitez-Benitez, S. J.; Davis, A. P.; Galan, M. Carmen. A vibration-induced-emission-based fluorescent chemosensor for the selective and visual recognition of glucose. *Angew. Chem. Int. Edit.* **2021**, *60*, 16880–16884.

3. Wang, B.; Chou, K. H.; Queenan, B. N.; Pennathur, S.; Bazan, G. C. Molecular design of a new diboronic acid for the electrohydrodynamic monitoring of glucose. *Angew. Chem. Int. Edit.* **2019**, *58*, 10612–10615.

4. Sharma, B.; Bugga, P.; Madison, L. R.; Henry, A. I.; Blaber, M. G.; Greeneltch, N. G.; Chiang, N.; Mrksich, M.; Schatz, G. C.; Duyne R. P. V. Bisboronic acids for selective, physiologically relevant direct glucose sensing with surface-enhanced Raman spectroscopy. *J. Am. Chem. Soc.* **2016**, *138*, 13952–13959.

5. Huang, Y. J.; Ouyang, W. J.; Wu, X.; Li, Z.; Fossey, J. S.; James, T. D.; Jiang, Y. B. Glucose sensing via aggregation and the use of "knock-out" binding to improve selectivity. *J. Am. Chem. Soc.* **2013**, 135, 1700–1703.

6. Liu, Y.; Deng, C. M.; Tang, L.; Qin, A. J.; Hu, R. R.; Sun, J. Z.; Tang, B. Z. Specific detection of D-glucose by a tetraphenylethene-based fluorescent sensor. *J. Am. Chem. Soc.* **2011**, *133*, 660–663.

7. Schiller, A.; Wessling, R. A.; Singaram, B. A fluorescent sensor array for saccharides based on boronic acid appended bipyridinium salts. *Angew. Chem. Int. Edit.* **2007**, *46*, 6457–6459.

8. Yang, W.; He, H.; Drueckhammer, D. G. Computer-guided design in molecular recognition: design and synthesis of a glucopyranose receptor. *Angew. Chem. Int. Edit.* **2001**, *40*, 1714–1718.

9. James, T. D.; Sandanayake, K. R. A. S.; Iguchi, R.; Shinkai, S. Novel saccharide-photoinduced electron transfer sensors based on the interaction of boronic acid and amine. *J. Am. Chem. Soc.* **1995**, *117*, 8982–8987.
